# Altermagnetic Magnons in Twisted van der Waals Antiferromagnets

**DOI:** 10.1021/acs.nanolett.6c00198

**Published:** 2026-04-07

**Authors:** Qirui Cui, Xiaocheng Bai, Yuqing Ge, Alexander Edström, Cong Li, Yasmine Sassa, Cheng Song, Kaiyou Wang, Anna Delin

**Affiliations:** † Department of Applied Physics, School of Engineering Sciences, KTH Royal Institute of Technology, AlbaNova University Center, SE-10691 Stockholm, Sweden; ‡ Swedish e-Science Research Center, KTH Royal Institute of Technology, SE-10044 Stockholm, Sweden; § School of Science, Xi’an University of Posts and Telecommunications, Xi’an 710121, China; ∥ Key Laboratory of Advanced Materials (MOE), School of Materials Science and Engineering, Tsinghua University, Beijing 100190, China; ⊥ State Key Laboratory of Superlattices and Microstructures, Institute of Semiconductors, Chinese Academy of Sciences, Beijing 100083, China; # Wallenberg Initiative Materials Science for Sustainability (WISE), KTH Royal Institute of Technology, SE-10044 Stockholm, Sweden

**Keywords:** van der Waals antiferromagnet, twist engineering, altermagnetic magnon, symmetry-controlled splitting, magnonic spin current

## Abstract

Magnonics promises low-dissipation information processing, yet spin-polarized magnon transport requires magnetic fields or spin–orbit couplings. Altermagnets exhibit spin-polarized electronic states and zero net magnetization. However, achieving large magnon spin splitting and robust magnonic spin currents remains challenging. Here we show that twisted van der Waals antiferromagnets provide a symmetry-tunable platform for the altermagnetic magnons. Alternating intralayer exchange arises in twisted bilayers lacking inversion and horizontal mirror symmetries, rendering nonrelativistic magnon spin splitting. Breaking out-of-plane rotational symmetries of a constituent monolayer significantly enhances low-energy splittings. We illustrate general conclusions in twisted CrPS_4_ (*d*-wave) and CrI_3_ (*i*-wave) bilayers. Moreover, pronounced field-free spin currents, characterized by robust spin Seebeck and spin Nernst effects, emerge in CrPS_4_. Remarkably, the spin transport is efficiently tuned by twist angle and exceeds that of conventional altermagnets by orders of magnitude. Our work provides novel insights into controlling magnons, deepening our fundamental understanding of altermagnetic spintronics.

## Graphical abstract

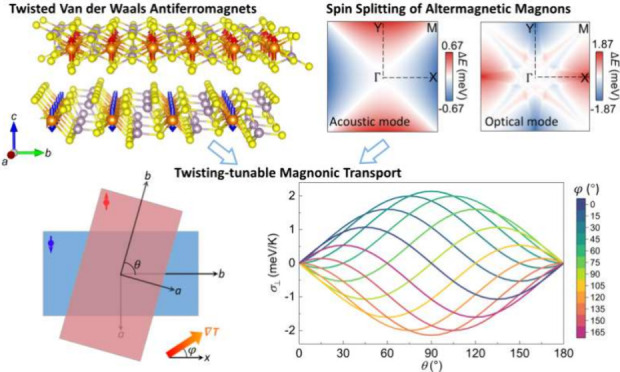

Magnons, quanta of spin waves, are promising information carriers in condensed matter, offering a potentially more energy-efficient alternative to electrons.^1–3^ The discovery of intrinsic magnetic order in two-dimensional crystals down to the monolayer limit has opened exciting opportunities for harnessing these collective spin excitations in ultrathin devices.^4–11^ The van der Waals (vdW) antiferromagnets, in particular, attract an extensive amount of attention due to their insulating features, rapid response times, and insensitivity to stray fields.^12–18^ However, the absence of net magnetization complicates the manipulation and detection of spin dynamics, typically requiring external magnetic fields^19,20^ and spin–orbit coupling.^21–23^ Realizing intrinsic spin-split magnons in vdW antiferromagnets thus remains a crucial objective, as it would minimize device complexity and power consumption.

Altermagnetic magnons exhibiting nonrelativistic spin splitting have recently been theoretically predicted in RuO_2_ ^24^ and experimentally confirmed in MnTe and CrSb.^25,26^ These magnons arise from alternating exchange interactions in fully compensated magnets with zero net magnetization, thus attracting a considerable amount of interest.^27–29^ Nevertheless, whether RuO_2_ exhibits magnetic or nonmagnetic order remains controversial,^30,31^ and under the nonrelativistic limit, a net spin current is forbidden in pristine MnTe and CrSb unless shear strain breaks the *g*-wave symmetry.^32^ These limitations underscore the pressing need for alternative hosts that support robust, altermagnetic magnons capable of generating strong field-free spin currents.

Notably, recent studies have shown that interlayer twisting in vdW antiferromagnets can induce nonrelativistic spin splitting in electronic bands.^33–37^ Moreover, advanced layer-transfer techniques now allow reliable fabrication of such bilayer with arbitrary interlayer twist angles.^55,56^ We thus infer that (i) interlayer twisting in vdW antiferromagnets may induce spin-split magnons analogous to electronic counterparts and (ii) if realized, these altermagnetic magnons would inherit distinctive vdW features, twist angle tunability and reduced intralayer symmetry, potentially exceeding those of conventional altermagnets. However, the interlayer twist-induced symmetry reduction immensely increases the complexity of the spin Hamiltonian, hindering a fundamental understanding of the spin dynamics, let alone verification of these intriguing possibilities.

In this study, by combining the symmetry analysis, variational approach, and first-principles modeling, we reveal the emergence of altermagnetic magnons arising from alternating intralayer spin interactions in twisted vdW antiferromagnets. We demonstrate that the twist-induced global symmetry breaking leads to intrinsic nonrelativistic spin splitting of magnon bands and highlight the critical role of reduced out-of-plane rotational symmetry in realizing spin splitting at low energies. More interestingly, such spin splitting can induce pronounced spin currents via the spin Seebeck and spin Nernst effects, which can be continuously tuned by the interlayer twist angle and exceed those in conventional altermagnets by orders of magnitude. Our findings thus provide an innovative pathway toward field-free, highly tunable spin caloritronics.

Without a loss of generality, a comprehensive symmetry analysis of spin arrangements and interactions is performed, specifically focusing on a bilayer system composed of two antiferromagnetically coupled ferromagnetic layers, i.e., A-type antiferromagnetism. **S**(**r**_*p*_, *z*) denotes the spin vector with position vector **r**_*p*_, and the coordinate *z* ∈ {−*a*, *a*} distinguishes two separate ferromagnetic layers. *J*(**r**_*p,q*_, *z*) denotes the intralayer Heisenberg exchange coupling between spin sites *p* and *q*, with in-plane translational vector **r**_*p,q*_. From the perspective of spin groups,^27,38^ we first consider the system to possess the $\left[C_{2}∥P\right]$ or $\left[C_{2}∥M_{z}\right]$ symmetry. Here, the $C_{2}$ operation denotes spin reversal, while P and $M_{z}$ denote the real-space inversion and horizontal mirror, respectively, interchanging atoms between opposite-spin sublattices. The symmetry constraints yield $S\left(r_{p},z\right)=\left[C_{2}∥P\right]S\left(r_{p},z\right)=-S\left(-r_{p},-z\right)$ or $S\left(r_{p},z\right)=\left[C_{2}∥M_{z}\right]S\left(r_{p},z\right)=-S\left(r_{p},-z\right)$ , guaranteeing that the magnetization from two layers fully compensates. Meanwhile, the invariance of the atomic structure under P or $M_{z}$ leads to $J\left(r_{p,q},z\right)=PJ\left(r_{p,q},z\right)=J\left(-r_{p,q},-z\right)$ or $J\left(r_{p,q},z\right)=M_{z}J\left(r_{p,q},z\right)=J\left(r_{p,q},-z\right)$ , indicating the equivalent intralayer exchange distribution for the bilayer. In contrast, twisting the bilayer can break both inversion and horizontal mirror symmetries of the atomic structure, thus allowing inequivalent intralayer exchange distributions for the two layers. Interestingly, a 2-fold rotational $C_{2∥}$ symmetry that lies in the plane of layers, with directional angle θ, interchanging atoms between opposite-spin sublattices, remains for any twist angle. Therefore, $S\left[r_{p}\left(\theta +\phi_{p}\right),z\right]=\left[C_{2}∥C_{2∥}\right]S\left[r_{p}\left(\theta +\phi_{p}\right),z\right]=-S\left[r_{p}\left(\theta -\phi_{p}\right),-z\right]$ holds for arbitrary angle ϕ_p_, where θ + ϕ_p_ is the directional angle of **r**_*p*_ and ϕ_*p*_ defines the direction difference between **r**_*p*_ and the $C_{2∥}$ axis. Despite the fact that both $\left[C_{2}∥P\right]$ and $\left[C_{2}∥M_{z}\right]$ symmetries are broken, $\left[C_{2}∥C_{2∥}\right]$ symmetry guarantees a zero net magnetization in the bilayer. Moreover, $J\left[r_{p,q}\left(\theta +\phi_{p,q}\right),z\right]=C_{2∥}J\left[r_{p,q}\left(\theta +\phi_{p,q}\right),z\right]=J\left[r_{p,q}\left(\theta -\phi_{p,q}\right),-z\right]$ holds for arbitrary angle ϕ_*p*,*q*_, where θ + ϕ_*p*,*q*_ is the directional angle of **r**_*p,q*_ and ϕ_*p*,*q*_ defines the direction difference between **r**_*p*,*q*_ and the $C_{2∥}$ axis. *J*[**r**_*p,q*_(θ + ϕ_*p,q*_),*z*] = *J*[**r**_*p,q*_(θ – ϕ_*p,q*_), – *z*] indicates that the intralayer exchange distributions possess a 2-fold symmetry across the $C_{2∥}$ axis.

Based on symmetry constraints, we further classify the crystal point groups of bilayers^35^ into three categories. In type I (*C*_*i*_, *C*_3*i*_, *C*_2*h*_, *C*_4*h*_, *C*_6*h*_, *D*_2*h*_, *D*_4*h*_, *D*_3*d*_, *D*_6*h*_, *C*_3*h*_, and *D*_3*h*_), $\left[C_{2}∥P\right]$ or $\left[C_{2}∥M_{z}\right]$ symmetry enforces fully compensated magnetic moments in bilayer antiferromagnets with point groups *C*_*i*_–*D*_6*h*_ or *C*_2*h*_–*D*_3*h*_, respectively. P or $M_{z}$ symmetry of atomic structure enforces equivalent exchange couplings in the two layers. In type II (*C*_2_, *D*_2_, *D*_3_, *D*_4_, *D*_6_, *D*_2*d*_, and *S*_4_), $\left[C_{2}∥C_{2∥}\right]$ or $\left[C_{2}∥S_{4z}\right]$ symmetry enforces fully compensated magnetization in bilayer antiferromagnets with point groups *C*_2_–*D*_2*d*_ or *D*_2*d*_, *S*_4_, respectively. The absence of P and $M_{z}$ symmetries of atomic structure renders the inequivalent intralayer exchange distributions of two sublattices. The intralayer exchange couplings are connected by $C_{2∥}$ or $S_{4z}$ . Interestingly, these point groups exactly match those responsible for altermagnetic electrons in bilayer systems.^34,35^ Although the influence of these point groups on spin arrangements and intralayer exchange is clear now, an accurate understanding of the spin dynamics requires resolution of the atomistic spin Hamiltonian of specific candidates. Based on the spin Hamiltonian, we find that besides in-plane rotational symmetry $C_{2∥}$ , out-of-plane rotational $C_{n⊥}\left(n>1\right)$ symmetry of constituent monolayer also plays a crucial role in shaping the magnon spectrum of the twisted bilayer. In type III (*C*_1_, *C*_*s*_, *C*_2*v*_, *C*_4_, *C*_4*v*_, *C*_3_, *C*_3*v*_, *C*_6_, and *C*_6*v*_), no symmetry (neither purely spatial nor combined with time reversal) connects the two opposite-spin sublattices, allowing non-zero net magnetization and inequivalent exchange distributions.

The atomistic spin Hamiltonian for vdW antiferromagnets, consisting of two ferromagnetic layers coupled antiferromagnetically, can be expressed as follows:

1 $$H=-\frac{1}{2}\underset{\gamma =1,2}{\sum }\overset{M}{\underset{p=1}{\sum }}H_{p}^{\gamma }-\overset{M}{\underset{p=1}{\sum }}H_{p}^{12}-K\underset{\gamma =1,2}{\sum }\overset{M}{\underset{p=1}{\sum }}\overset{N}{\underset{i=1}{\sum }}{\left(S_{i,p}^{\gamma ,z}\right)}^{2}$$

which includes intralayer exchange, interlayer exchange, and magnetic anisotropy contributions. γ denotes the layer index, with γ = 1 and 2 corresponding to the top and bottom layer, respectively. *p* denotes the spin sites within the unit cell, and *i* denotes the different unit cells. *H*_*p*_^γ^ = ∑_*i*_∑_*q*_ *J*_*p,q*_ **S**_*i,p*_^γ^·**S**_*i*+δ,*q*_^γ^, and *H*_*p*_^12^ = ∑_*i*_∑_*q*_ *J*_*p,q*_^*c*^ **S**_*i,p*_^1^·**S**_*i*+δ′,*q*_^2^, where *J*_*p*,*q*_ and *J*_*p,q*_^*c*^ represent intralayer and interlayer exchange, respectively. Note that the factor of 1/2 of the intralayer interactions in eq 1 accounts for double counting arising from summation over intralayer spin sites.

Before investigating specific materials, we gain general insights into magnons in twisted antiferromagnets from a variational approach, under the assumption of weak interlayer coupling, as appropriate in vdW materials. By introducing magnon creation operators α_**k**,*i*_^†^ = ∑_*m*_ *c*(**k**)_*i,m*_e^*iϕ*(**k**)_*i,m*_^ *a*_**k**,*m*_^†^ with band index *i*, spin site index *m*, real coefficient *c*(**k**)_*i,m*_, and phase ϕ­(**k**) into eq 1 and neglecting *H*_*p*_^12^, the variational approach yields

2 $$\left\langle 0\right|{\mathrm{\alpha ̂}}_{k,i}\left[\psi_{k}^{†}H_{k}\psi_{k}\right]{\mathrm{\alpha ̂}}_{k,i}^{†}\left|0\right\rangle =2K+\frac{1}{2}\underset{p\neq q}{\sum }J_{p,q}\left[c{\left(k\right)}_{i,p}^{2}+c{\left(k\right)}_{i,q}^{2}-2c{\left(k\right)}_{i,p}c{\left(k\right)}_{i,q}\;\cos\left(\Delta {\left(k\right)}_{p,q}^{i}\right)\right]$$

with the phase difference Δ­(**k**)_*p,q*_^*i*^ = **k**·**R**_*p,q*_ + ϕ_*i,q*_(**k**) – ϕ_*i,p*_(**k**), and **R**_*p,q*_ is the lattice vector connecting the unit cells of spin sites *p* and *q*. For the acoustic mode, the minimum eigenvalue of *H*_**k**_ requires minimizing ⟨0|α̂_**k**,*i*_[ψ_**k**_^†^ *H*_**k**_ψ_**k**_]­α̂_**k**,*i*_^†^|0⟩. This condition results in a weight factor that satisfies the relationship *c*(**k**)_*i,p*_^2^ ≈ *c*(**k**)_*i,q*_^2^ across the Brillouin zone (except at the boundary), and a minimized phase difference Δ­(**k**)_*p,q*_^*i*^. When lattice vectors **R**_*p,q*_ are non-zero, phase differences Δ­(**k**)_*p,q*_^*i*^ increase with the magnitude of **k** as its orientation is fixed. The acoustic magnon energy thus increases along the same momentum direction. Notably, the different exchange *J*_*p*,*q*_ values along different crystallographic directions can lead to anisotropic acoustic modes. Specifically, when the constituent monolayer possesses only $C_{2⊥}$ symmetry on the atomic structure, the anisotropic *J*_*p*,*q*_ along the main axes results in elliptical iso-energy contours. The twisting generates inequivalent intralayer exchange distributions, breaking the overlap of elliptical iso-energy contours from top and bottom layers and thus inducing strong spin splitting in the acoustic magnon spectra (Figure 1D). However, the higher orders of out-of-plane rotational symmetry, such as $C_{3⊥}$ , $C_{4⊥}$ , and $C_{6⊥}$ , enforce isotropic intralayer exchange couplings along main crystallographic axes. This isotropy leads to nearly circular iso-energy contours, resulting in negligible spin splitting effects in low-energy states. On the other hand, the magnon energy is not the minimal eigenvalue of *H*_**k**_ for optical modes. Weight factors *c*(**k**) do not equal each other for different spin sites, and phase differences Δ­(**k**)_*p,q*_^*i*^ can approach π even between neighboring sites. These factors introduce complexity into the optical branch structures. Therefore, even under high out-of-plane rotational symmetries, the twisting may induce observable spin splitting effects in the optical magnon branches. Within the type II category, the A-type antiferromagnets with point groups *C*_2_ and *D*_2_ lack higher out-of-plane rotational symmetries for spin sublattices and thus can exhibit sizable acoustic magnon splitting. In contrast, other point groups allow sizable spin splitting only in the optical modes. Next, we explicitly illustrate these general conclusions in two representative materials CrPS_4_ and CrI_3_, with 2- and 3-fold out-of-plane rotational symmetry, respectively.

**Figure 1:**
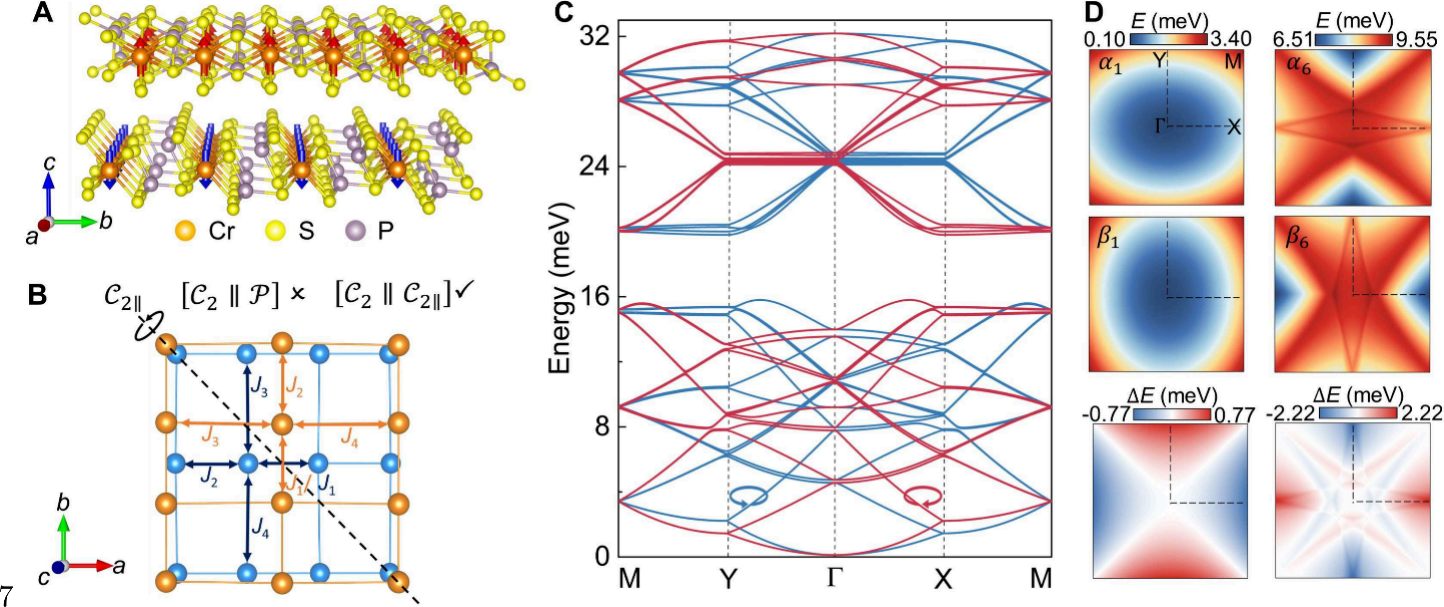
(A) Side view of the CrPS_4_ crystal structure. (B) Close-up of the antiferromagnetic sublattices, showing definitions of the interatomic Cr–Cr exchange couplings employed in our model. Blue and yellow atoms represent Cr atoms in the lower and upper layers, respectively. The spin group symmetries are also indicated, highlighting the breaking of $\left[C_{2}∥P\right]$ symmetry and the preservation of $\left[C_{2}∥C_{2∥}\right]$ symmetry, which determines the spin arrangements and spin interactions. (C) Spin-split magnon spectra of right-handed α (blue lines) and left-handed β (red lines) modes. (D) Energy dispersion mappings of α and β modes for branches 1 and 6, respectively (top and middle panels), and their energy difference *ΔE* (bottom panel), exhibiting the *d*-wave feature.

For resolving spin dynamics in realistic twisted candidates, we first use density functional theory calculations in the generalized gradient approximation^39^ with Hubbard correction. Exchange parameters are calculated from using the magnetic force theorem,^40–42^ fully determining the atomistic spin Hamiltonian (eq 1) of the supercell of twisted antiferromagnets, including both interlayer and intralayer couplings. More detailed computational approaches are given in section I of the Supporting Information (SI). After the Holstein–Primakoff transformation, the bosonic Hamiltonian in momentum space is given by *H* = ∑_**k**_ψ_**k**_^†^ *H*_**k**_ψ_**k**_, where bosonic operator ψ_**k**_ = [*a*_**k**_, *b*_**k**_^†^]^*T*^ is defined with *a*_**k**_^†^ ≡ [*a*_**k**,1_^†^, ..., *a*_**k**,*M*_^†^] and *b*_**k**_^†^ ≡ [*b*_**k**,1_^†^, ..., *b*_**k**,*M*_^†^]. Finally, this bosonic Hamiltonian is diagonalized by the Bogoliubov transformation, ψ_**k**_ = *T*_**k**_Ψ_**k**_ with Ψ_**k**_ = [α_**k**_, β_**k**_^†^]^*T*^, where key paraunitary matrix *T*_**k**_ is obtained using a Cholesky decomposition.^43^ The specific formats of the bosonic Hamiltonian and detailed diagonalization processes are given in sections II and III of the SI.

We first consider vdW material CrPS_4_ (point group *C*_2*h*_), exhibiting intralayer ferromagnetic and interlayer antiferromagnetic couplings, which has been identified as an ideal platform for long-distance magnon transport,^44,45^ owing to its robust magnetic ordering and insulating properties.^46–50^ The monolayer consists of quasi-one-dimensional chains of distorted CrS_6_ octahedra interconnected by P atoms. The local magnetic moment reaches 3.19 μ_B_ per Cr, consistent with the *S* = 3/2 state for Cr^3+^. The in-plane rectangular lattice constants satisfy *a*/*b* ≈ 3/2, which allows construction of a commensurate bilayer (2*a* ≈ 21.89 Å) by rotating one layer by 90° (Figure 1A). The point group of the twisted bilayer is reduced to *C*_2_ that allows the inequivalent intralayer exchange between bilayers.

The CrPS_4_ monolayer lacks out-of-plane rotational symmetry but possesses the combined $C_{2⊥}$ $M_{z}$ symmetry on atomic structure. Since $M_{z}$ does not affect the intralayer Heisenberg exchange couplings, the exchange couplings are solely governed by $C_{2⊥}$ symmetry, allowing anisotropic exchange along different main axes. Figure 1B shows an enlarged view of the spin–lattice in twisted bilayer CrPS_4_. Breaking the P and $M_{z}$ symmetry allows for an inequivalent distribution of intralayer exchange along different main axes for the two opposite-spin sublattices. Additionally, the exchange distribution for the two layers can be transformed by the preserved $C_{2∥}$ symmetry. Despite the twist-induced distinct local environments experienced by the Cr atoms in a single layer, the strongly anisotropic intralayer exchange pattern is well preserved in the twisted bilayer due to the weak vdW couplings. Specifically, for the Cr1-1 site in the top layer (Figure S1), the intralayer exchange constants along the *b* axis (*J*_1_ = 5.039 meV, and *J*_2_ = 3.555 meV) are an order of magnitude larger than those along the *a* axis (*J*_3_ = 0.486 meV, and *J*_4_ = 0.481 meV). This magnitude relationship is reversed in the bottom layer (Figure S3), confirming the inequivalent exchange distributions between two opposite-spin layers. The interlayer exchange interactions involve competing ferro- and antiferromagnetic couplings from different neighbors, but the overall outcome favors antiferromagnetism, such as *J*_1_^*c*^ = 0.004 meV, *J*_2_^*c*^ = −0.031 meV, *J*_3_^*c*^ = −0.003 meV, *J*_4_^*c*^ = −0.028 meV, and *J*_5_^*c*^ = 0.007 meV for Cr1-1 (Table S2). This feature is consistent with the situation in pristine CrPS_4_.^51^ Despite the dependence of individual interlayer couplings on *U*_eff_ in the DFT calculations, the total energy contribution to the spin Hamiltonian remains around −1.5 meV across *U*_eff_ = 1–4 eV (Figure S9), favoring robust interlayer antiferromagnetism. Importantly, the intralayer/interlayer exchange couplings obtained from first principles (Tables S1 and S2) for the top and bottom layers can be transformed into each other by the $C_{2∥}$ operation.

Figure 1C shows a clearly spin-split magnon spectrum in twisted CrPS_4_, featuring nondegenerate α and β magnon modes characterized by opposite *z*-component spin angular momenta (in units of ℏ), i.e., ⟨0|α̂_*k*_ *S* ^*z*^α̂_*k*_^†^|0⟩ = −1 and ⟨0|β̂_*k*_ *S* ^*z*^β̂_*k*_^†^|0⟩ = 1. Figure 1D elucidates that α and β magnon dispersions exhibit anisotropy along different main crystal axes; meanwhile, these two modes can be transformed into each other by the $\left[C_{2}∥C_{2∥}\right]$ operation. Subscripts 1 and 6 of α denote magnon branch index *n* obtained from the paraunitary diagonalization of the CrPS_4_ bosonic Hamiltonian. Specifically, *n* values of 1 and 6 indicate the lowest and sixth energy branches at Γ, respectively (see Figure 1C). Alternating energy difference *ΔE* highlights magnon nondegeneracy throughout the Brillouin zone, except along the Γ–M direction, corresponding to *d*-wave altermagnetsim. Spin splitting arises from the anisotropic intralayer exchange along distinct main axes in twisted structures and is insensitive to interlayer couplings (Figure S10). The anisotropic magnon spectrum indicates the presence of direction-dependent magnon velocities (Figure S11). For example, the maximum group velocities are *v*_*x*_^α^ ≈ 2640 m/s and *v*_*x*_^β^ ≈ 4260 m/s in the acoustic branch. The anisotropy is inverted between the α and β modes; i.e., the direction of larger group velocity in the α mode corresponds to the smaller one in the β mode, and vice versa. The magnon dispersions display a quadratic characteristic very close to the Γ point since magnetic anisotropy *K* is included in the spin Hamiltonian, in contrast to the linear dispersion observed when *K* is absent (Figure S16).

Next, we consider the CrI_3_ bilayer with a 21.79° twisted angle (Figure 2A) to confirm that, while altermagnetic optical magnons generally emerge in twisted vdW antiferromagnets, 3-fold or higher rotational symmetry of the monolayer precludes spin splitting of the acoustic magnon modes. The point group of the monolayer is *D*_3*d*_, and it is reduced to *D*_3_ in the twisted bilayer. As shown in Figure 2B, inequivalent intralayer exchange for the bilayer is related by the operation $C_{2∥}$ . The spin-split magnon spectrum shows nondegenerate α and β modes (Figure 2C–E), corresponding to *i*-wave altermagnetism. Different from that of CrPS_4_, the $C_{3⊥}$ symmetry of CrI_3_ indicates that the isotropic intralayer exchange interactions forbid an observable spin splitting of acoustic magnon modes, as predicted by the previous model analysis. Specifically, the maximum splitting of acoustic modes (∼0.002 meV) in CrI_3_ (Figure 2D) is 2 orders of magnitude smaller than that (∼0.77 meV) in CrPS_4_ (Figure 1D), and non-negligible alternating spin splitting emerges only in optical branches (Figure 2E).

**Figure 2:**
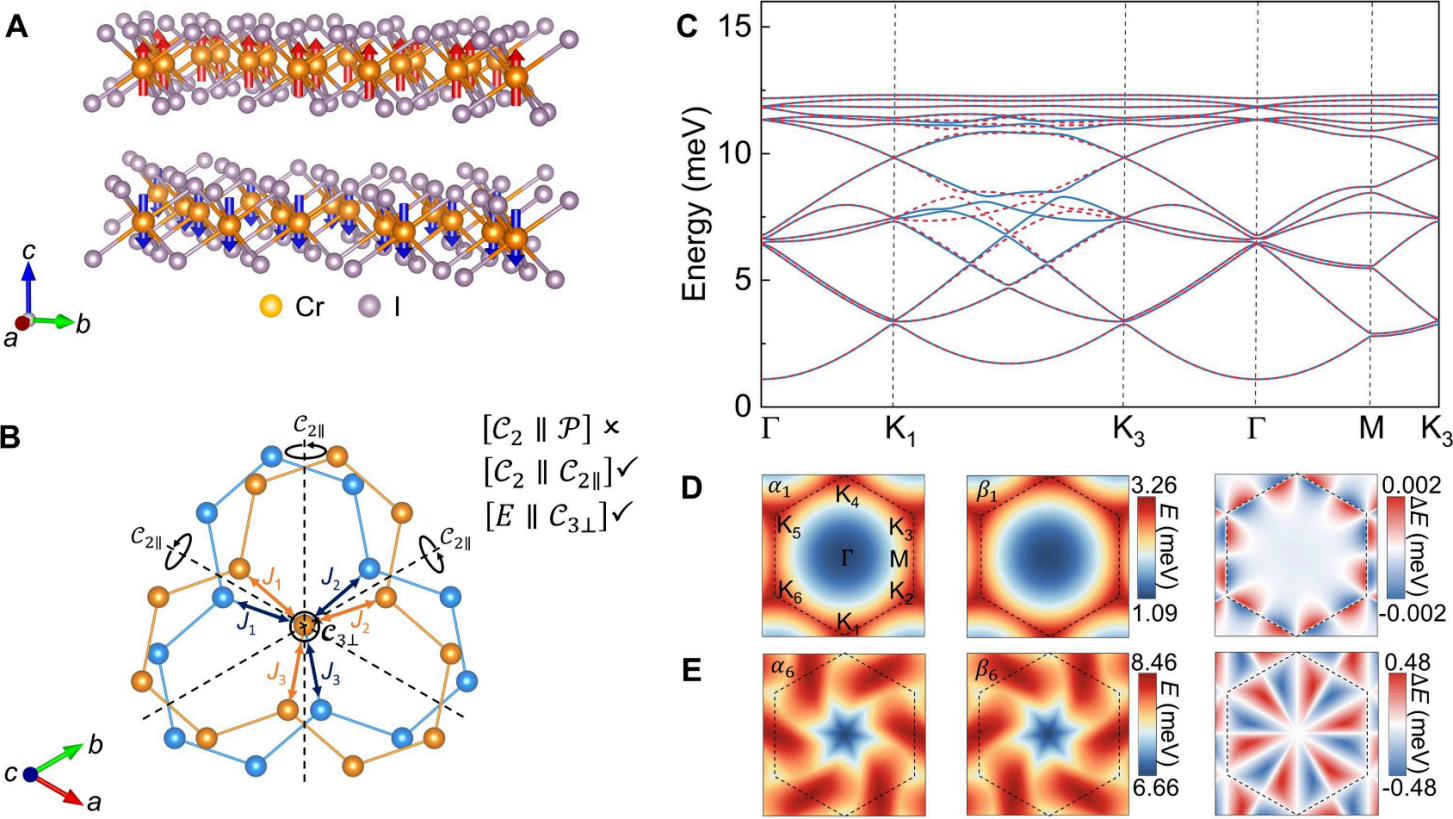
(A) Side view of the CrI_3_ crystal structure. (B) Close-up view of the antiferromagnetic sublattices, showing definitions of the interatomic Cr–Cr exchange couplings. The spin group symmetries are labeled, i.e., the breaking of $\left[C_{2}∥P\right]$ symmetry and the preservation of $\left[C_{2}∥C_{2∥}\right]$ and $\left[E∥C_{3⊥}\right]$ symmetries. Compared to CrPS_4_, the additional $C_{3⊥}$ structural symmetry ensures that intralayer exchange couplings remain quasi-isotropic along three crystallographic directions. (C) Spin-split magnon spectra of α (right-handed) and β (left-handed) modes, which are denoted by blue solid lines and red dashed lines, respectively. (D and E) Energy dispersion mappings of α and β modes for branches 1 and 6, respectively (left and middle panel), and their energy difference *ΔE* (right panel), exhibiting the typical *i*-wave feature.

Now we focus on the technologically relevant and experimentally observable transport phenomenon mediated by altermagnetic magnons. Spin conductivities are obtained using the Kubo formalism applied to the resolved magnonic eigenstates (section IV of the SI). For the *d*-wave magnons in CrPS_4_, we find the thermal spin conductivities along main axes σ_*xx*_^α^ = −2.53 meV/K and σ_*yy*_^α^ = −4.58 meV/K at 15 K, which corresponds to an anisotropy ratio of about 75%. Neglecting the sign of σ that indicates the polarization orientation of magnon currents, the relative magnitudes of σ_*xx*_ and σ_*yy*_ are reversed for the β mode; i.e., σ_*xx*_^β^ = 4.58 meV/K, and σ_*yy*_^β^ = 2.53 meV/K. In contrast, the relationship σ_*xx*_^α^ = σ_*yy*_^α^ = −σ_*xx*_^β^ = −σ_*yy*_^β^ is well preserved for the *i*-wave magnons in CrI_3_ (Figure S12), indicating isotropic magnon transport. Figure 3A shows that when a thermal gradient is applied along the main axes (φ = 0°, 90°, 180°, ...), the inequivalent magnon currents from the α and β modes produce a finite longitudinal spin conductivity σ_∥_ of 2.05 meV/K. This response is termed the spin Seebeck effect. Importantly, along the two orthogonal main axes, the spin polarization of the current is opposite due to the alternating features of the α and β modes. Once the thermal gradient deviates from the main axes, nonequivalent σ_*xx*_ and σ_*yy*_ renders not only longitudinal spin conductivity σ_∥_ but also transverse component σ_⊥_ (Figure 3A). When the gradient is along a diagonal direction (φ = 45°, 135°, 225°, ...), transverse spin conductivity σ_⊥_ reaches a maximum of 2.05 meV/K, indicating a spin Nernst effect. This record current strength is several orders of magnitude higher than the Hall response originating from the spin Berry curvature, greatly enhancing the experimental feasibility of spin current generation and detection.^21,52–54^ Importantly, there is no net heat current along the direction of this transverse spin flow because the Hall-like heat currents carried by the α and β modes cancel out and yield a pure spin current. Despite various spin transport phenomena in CrPS_4_ (Figure 3A), no spin signal is detected for *i*-wave magnon CrI_3_ (Figure 3B). This absence is attributed to the coexistence of $\left[E∥C_{3⊥}\right]$ and $\left[C_{2}∥C_{2∥}\right]$ symmetries, which enforce equal α and β contributions in all in-plane directions. We can artificially introduce scaling factor *g* into the top-layer exchange couplings while preserving the bottom-layer exchange couplings, to break the $\left[C_{2}∥C_{2∥}\right]$ symmetry. Figure 3B shows that this symmetry reduction yields an isotropic, *g*-dependent spin current with σ_∥_ = −0.17 (−0.48) meV/K at *g* = 1.2 (2).

**Figure 3:**
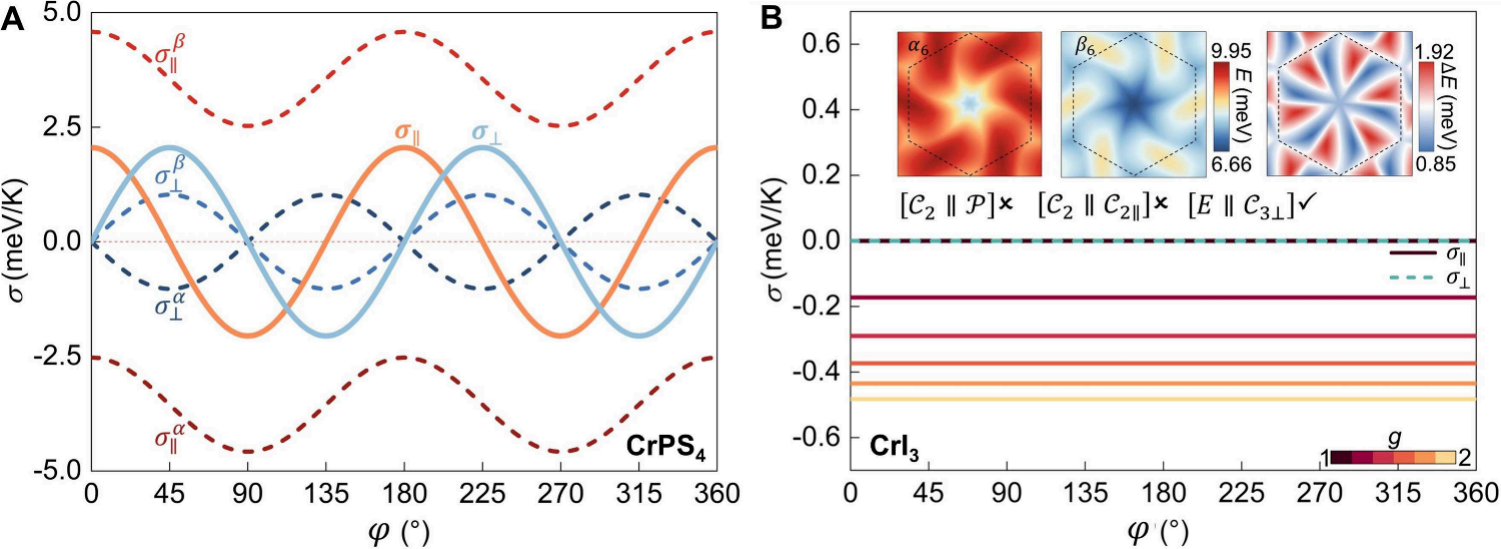
Spin conductivity of the thermally driven magnon current in the twisted bilayer at 15 K, shown as a function of the thermal gradient orientation. (A) For CrPS_4_, the dashed lines represent the longitudinal and transverse conductivity contributions from the α and β modes separately while the solid lines indicate the final combined signal. (B) For CrI_3_, the longitudinal (solid line) and transverse (dashed line) spin conductivities both vanish in the pristine state. The inset energy dispersion mapping shows that as *g* > 1 (*g* = 1.2) breaks the $\left[C_{2}∥C_{2∥}\right]$ symmetry, the α and β mode energy levels split. Consequently, an isotropic longitudinal spin current is generated, while the transverse component remains at zero.

The interlayer twist angle in 2D materials serves as an emergent degree of freedom, enabling precise control of physical phenomena. For single-layer CrPS_4_, we find the σ_*aa*_^↑^ = −σ_*aa*_^↓^ = 4.77 and σ_*bb*_^↑^ = −σ_*bb*_^↓^ = 2.63 meV/K. By neglecting weak interlayer couplings, we derive expressions for longitudinal and transverse spin conductivities for arbitrary twist angles θ and thermal gradient orientation φ (Figure S13A and section V of the SI): σ_∥_ = (σ_*aa*_^↑^ – σ_*bb*_^↑^) sin θ sin­(θ – 2φ) and $\sigma_{⊥}=\frac{\left(\sigma_{aa}^{↑}-\sigma_{\mathrm{bb}}^{↑}\right)}{2}\left[\sin\left(2\theta -2\varphi \right)+\sin2\varphi \right]$ . When θ = 90°, we confirmed excellent agreement between calculations including or neglecting interlayer exchange (Figure S13B). Notably, as shown in Figure 4, both σ_∥_ and σ_⊥_ are continuously modulated by the interlayer twisting angles across all thermal gradient orientations. When thermal gradient orientation φ = 0°, σ_∥_ reaches its maximum of 2.14 at a twist angle θ = 90° and then gradually decreases to 0 meV/K as θ approaches 0° or 180°. Upon tuning the thermal gradient away from the main axis such as φ = 30°, σ_∥_ initially attains its maximum spin-down conductivity of −0.54 at θ = 30° and subsequently transitions to its maximum spin-up conductivity of 1.60 meV/K at θ = 120°. In contrast, when φ = 0, σ_⊥_ reaches its maximum of 1.07 at θ = 45°, and when φ = 45°, σ_⊥_ reaches a higher maximum of 2.14 meV/K at θ = 90°. These observations highlight distinct optimal configurations. Peak σ_∥_ occurs at (θ = 90°, φ = 0°, 90°, 180°, ...), and peak σ_⊥_ is realized at (θ = 90°, φ = 45°, 135°, 225°, ...).

**Figure 4:**
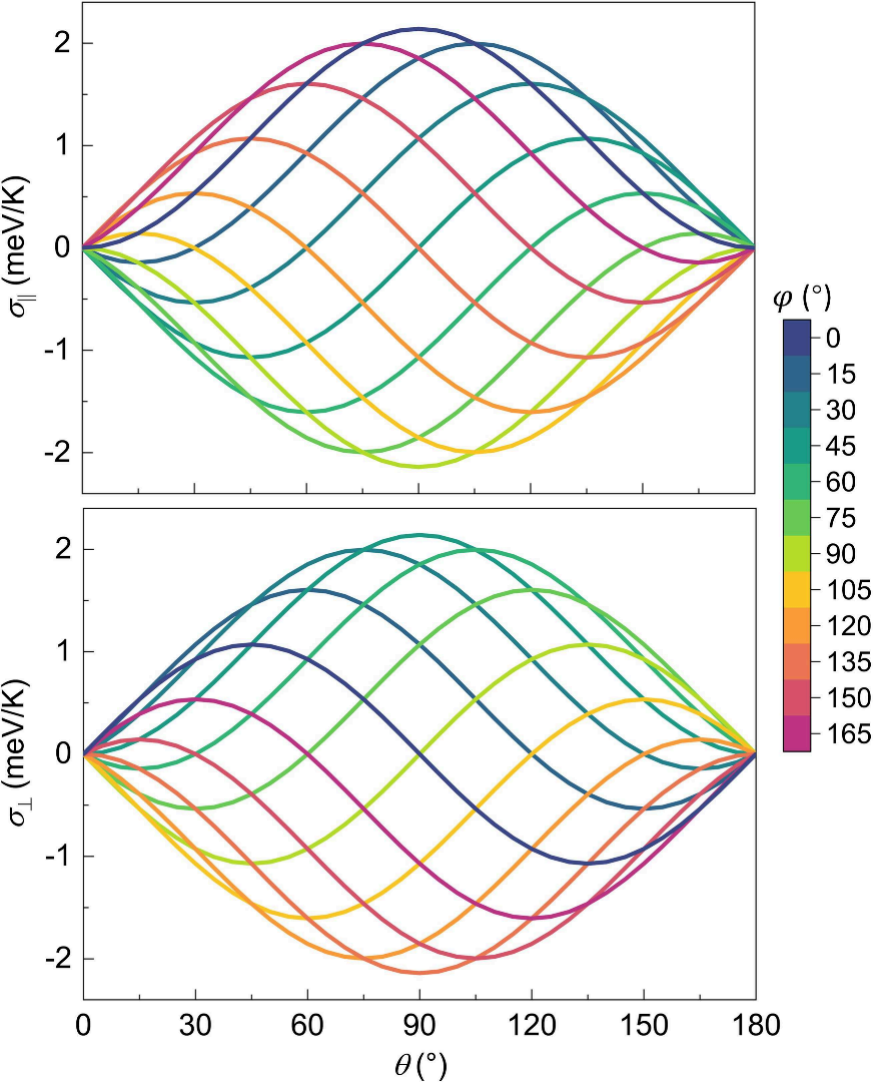
Longitudinal spin conductivity σ_∥_ and transverse spin conductivity σ_⊥_ as functions of interlayer twisting angle θ and thermal gradient orientation φ. The maximum magnitude of σ_∥_ and σ_⊥_ reaches 2.14 meV/K at optimal spin configurations, fairly consistent with the value of 2.05 meV/K derived from the explicit spin Hamiltonian of the twisted antiferromagnetic bilayer. This small magnitude discrepancy arises due to the neglect of interlayer couplings in calculations of θ-dependent magnonic transport.

We benchmark the spin signals of twisted antiferromagnets against nontwisted *d*-wave altermagnets, exemplified by recently reported Fe_2_Se_2_O and Mn_2_PTe monolayers^57,58^ (Figure S14 and section VI of the SI). The maximum magnitudes of spin conductivity are approximately 0.12 × 10^–3^ and 0.45 × 10^–3^ meV/K for Fe_2_Se_2_O and Mn_2_PTe, respectively (Figure S15). Even when the very large *K* is negelected to enhance magnonic occupation, the spin conductivity increases to 0.04 and 0.19 meV/K, remaining at least 1 order of magnitude lower than the value of 2.05 meV/K in twisted CrPS_4_. This substantially enhanced spin signal in twisted antiferromagnets arises from (i) the inherently weaker interlayer couplings in vdW magnets compared to intralayer interactions and (ii) the reduced intralayer crystal symmetry inducing magnonic anisotropy along different main axes in a single layer, resulting in the α and β modes exhibiting a large anisotropy at low energies.

In summary, we have established a symmetry-based theoretical framework for understanding spin dynamics in twisted bilayer antiferromagnets, finding that the breaking of twist-induced inversion and horizontal mirror symmetries results in inequivalent intralayer exchange couplings between the top and bottom layers, leading to a nonrelativistic spin splitting between the α and β magnon branches. Critically, an observable splitting of low-energy acoustic magnons arises only if the constituent monolayers lack out-of-plane rotational symmetry (except for $C_{2⊥}$ ), providing clear guidelines for efficiently harnessing the spin degree of freedom. The developed computational approach can be applied to accurately resolve magnon spectra in other twisted systems. In principle, altermagnetic spin dynamics can be realized in any twisted vdW antiferromagnet. The resulting *d*-wave altermagnetic magnons yield exceptionally robust spin Seebeck and spin Nernst effects. In contrast to nontwisted altermagnets, the unique nature of vdW magnets enables not only efficient tuning of magnonic transport via the interlayer twist angle but also net spin signal enhancement by orders of magnitude.

## Supplementary Material



## Notes

Methods for first-principles calculations (section I), explicit spin Hamiltonian of CrPS_4_ and CrI_3_ (section II), details of bosonic Hamiltonian and Bogoliubov transformation (section III), Kubo theory for magnonic transport (section IV), twist-tunable spin transport (section V), comparison with nontwisted altermagnets (section VI), band disperisons close to the Γ point (section VII), and additional supporting data, including the real-space distributions and magnitudes of exchange couplings in twisted CrPS_4_ and CrI_3_, interlayer exchange-dependent magnon dispersion, group velocities of acoustic modes, and temperature-dependent σ_*xx*_ and σ_*yy*_ and their anisotropy ratio (PDF)
