# Supplementary material for: Altermagnetic Magnons in Twisted van der Waals Antiferromagnets
Source: Nano Lett. 2026 Apr 7;26(15):5078–85. doi: 10.1021/acs.nanolett.6c00198 (PMC13107460; doi:10.1021/acs.nanolett.6c00198)
Supplement: Supplementary file 1 [file nl6c00198_si_001.pdf]

Supporting Information for "Altermagnetic Magnon in Twisted van der Waals Antiferromagnets"

Qirui Cui[1,2], Xiaocheng Bai[3*], Yuqing Ge[1], Alexander Edström[1,2,6], Cong Li[1], Yasmine Sassa[1], Cheng Song[4], Kaiyou Wang[5*], Anna Delin[1,2,6*]

[1]Department of Applied Physics, School of Engineering Sciences, KTH Royal Institute of Technology, AlbaNova University Center, SE-10691 Stockholm, Sweden.
[2]Swedish e-Science Research Center, KTH Royal Institute of Technology, SE-10044 Stockholm, Sweden.
[3]School of Science, Xi'an University of Posts and Telecommunications, Xi'an 710121, China.
[4]Key Laboratory of Advanced Materials (MOE), School of Materials Science and Engineering, Tsinghua University, Beijing, China.
[5]State Key Laboratory of Superlattices and Microstructures, Institute of Semiconductors, Chinese Academy of Sciences, Beijing 100083, China.
[6]Wallenberg Initiative Materials Science for Sustainability (WISE), KTH Royal Institute of Technology, SE-10044 Stockholm, Sweden.

*Corresponding author(s). E-mail(s): xcbai@mail.ustc.edu.cn; kywang@semi.ac.cn; annadel@kth.se;

I. *The first − principles calculations*—We use the magnetic force theorem, also called the Liechtenstein–Katsnelson–Antropov–Gubanov (LKAG) formalism derived from real-space Green's functions, to calculate the exchange couplings [1, 2]. Specifically, the LKAG expression for the Heisenberg exchange is given by $J_{ij} = -\frac{1}{4\pi}\int_{-\infty}^{E_f} \mathrm{Im}\Big\{\mathrm{Tr}\Big[\delta_{ii}\, G_{ij}(\varepsilon, \mathbf{R})\, \delta_{jj}\, G_{ij}(\varepsilon, -\mathbf{R})\Big]\Big\}\, d\varepsilon$, where $\mathbf{R}$ is the lattice vector linking spin sites, and δ denotes the difference between the spin-up and spin-down parts of the Hamiltonian matrix. The exchange coupling calculations were performed using the QuantumATK (QATK) package [3], which expands electronic states via a linear combination of atomic orbitals (LCAO). Because the LCAO method utilizes a finite, localized basis set, it requires notably fewer basis functions than plane-wave-based approaches [4, 5]. This distinction is particularly beneficial for our twisted systems, which involve large vacuum regions and many atoms. The unit cell of bilayer $CrPS_4$ with a twisted angle 90° contains 288 atoms, and the unit cell of bilayer CrI_3 with a twisted angle 21.79° contains 112 atoms. A spin-polarized Generalized Gradient Approximation (GGA)+U functional with $U_{\mathrm{eff}} = 3\,\mathrm{eV}$ on the Cr-3d orbitals was adopted, and additional tests with $U_{\mathrm{eff}} = 1, 2$ and $4\,\mathrm{eV}$ confirmed that our main conclusions remain robust against variations in U_{eff}. A cutoff energy of 130 Ha and a $4 \times 4 \times 1$ k-point mesh were employed, with an energy convergence criterion set to 10^{-7} eV to ensure high computational precision.

For a system containing more than one hundred atoms, incorporating spin–orbit coupling (SOC) effects is computationally challenging. Fortunately, owing to the weak van der Waals interactions in the monolayer, we can use the single-ion magnetic anisotropy determined from the monolayer as a proxy for that in the twisted bilayer system. The single-ion magnetic anisotropy K was then determined using the Vienna ab initio simulation package (VASP) [6–8] by comparing the self-consistent energies corresponding to magnetization along the x- and z-directions. We find that $K = 0.028$ and 0.339 meV for $CrPS_4$ and CrI_3, respectively, favoring the out-of-plane magnetization. We note that the altermagnetic spin-splitting is a nonrelativistic effect, and our general conclusions remain independent of SOC. The magnetic anisotropy primarily contributes to generate a gap at the Γ point of the acoustic magnon spectrum. Specifically, all crystal structures are fully relaxed until the Hellmann–Feynman forces on each atom were below 0.01 eV/Å before calculating the magnetic parameters. To accurately determine interlayer distances and capture possible moiré reconstructions, all atoms are allowed to move freely within the heterostructure geometry during relaxation. The vdW interactions are treated using Grimme's DFT-D3 method with the zero-damping function [9].

II. *Spin Hamiltonian of twisted bilayer* $CrPS_4$ *and* CrI_3—The spin Hamiltonian including the intralayer exchange couplings, interlayer exchange couplings and magnetic anisotropy can be written as:

$$H = -\frac{1}{2}\sum_{\gamma=1,2}\sum_{p=1}^{24} H_p^{\gamma} - \sum_{p=1}^{24} H_p^{12} - K\sum_{\gamma=1,2}\sum_{p=1}^{24}\sum_{i=1}^{N}(S_{i,p}^{\gamma,z})^2, \tag{1}$$

where γ denotes the layer index, with $\gamma = 1$ and 2 corresponding to the top and bottom layer respectively. Meanwhile, p denotes the spin sites within the unit cell, and i denotes the different unit cells. The dominant exchange couplings that determine the magnon spectra were selected to extend to at least the fourth-nearest neighbors (see Figures S1–S8), as comprehensively validated in Refs. [10–13]. Accordingly, we estimate and give the intralayer exchanges (with bond lengths below 7 Å) and the interlayer exchanges (with bond lengths below 8.5 Å) for twisted $CrPS_4$, and below 7 Å and 9 Å for twisted CrI_3, which are summarized in Tables S1-S4. In twisted bilayer $CrPS_4$, there are 48 Cr atoms in total. We use the Cr1-1 (see specific position in Fig. S1) as an illustrative example to deduce the magnon Hamiltonian. The identical approach is then applied for other Cr atoms. For Cr1-1, we consider the intralayer exchange couplings as follow

$$\begin{aligned} H_1^1 = \sum_i & J_{1,2}^1 \boldsymbol{S}_{i,1}^1 \cdot \boldsymbol{S}_{i,2}^1 + J_{1,4}^1 \boldsymbol{S}_{i,1}^1 \cdot \boldsymbol{S}_{i-a,4}^1 + J_{1,5}^1 \boldsymbol{S}_{i,1}^1 \cdot \boldsymbol{S}_{i,5}^1 + J_{1,21}^1 \boldsymbol{S}_{i,1}^1 \cdot \boldsymbol{S}_{i-b,21}^1 \\ &+ J_{1,6}^1 \boldsymbol{S}_{i,1}^1 \cdot \boldsymbol{S}_{i,6}^1 + J_{1,8}^1 \boldsymbol{S}_{i,1}^1 \cdot \boldsymbol{S}_{i-a,8}^1 + J_{1,24}^1 \boldsymbol{S}_{i,1}^1 \cdot \boldsymbol{S}_{i-a-b,24}^1 + J_{1,22}^1 \boldsymbol{S}_{i,1}^1 \cdot \boldsymbol{S}_{i-b,22}^1, \end{aligned} \tag{2}$$

where J represents the exchange couplings whose magnitudes are given in Table S1. $\boldsymbol{S}_i$ represents the spin vectors within unti lattice cell i. a and b label the basis vector along the x and y directions. For Cr1-1, we consider the interlayer exchange couplings as follow

$$\begin{aligned} H_1^{12} = \sum_i & J_{1,1}^{12} \boldsymbol{S}_{i,1}^1 \cdot \boldsymbol{S}_{i,1}^2 + J_{1,5}^{12} \boldsymbol{S}_{i,1}^1 \cdot \boldsymbol{S}_{i,5}^2 + J_{1,9}^{12} \boldsymbol{S}_{i,1}^1 \cdot \boldsymbol{S}_{i,9}^2 \\ &+ J_{1,2}^{12} \boldsymbol{S}_{i,1}^1 \cdot \boldsymbol{S}_{i,2}^2 + J_{1,6}^{12} \boldsymbol{S}_{i,1}^1 \cdot \boldsymbol{S}_{i,6}^2 + J_{1,10}^{12} \boldsymbol{S}_{i,1}^1 \cdot \boldsymbol{S}_{i,10}^2. \end{aligned} \tag{3}$$

Using the Holstein–Primakoff transformation $S_{i,p}^{p,z} = S - a_{i,p}^{\dagger} a_{i,p}$, $S_{i,p}^{1,+} = \sqrt{2S} a_{i,p}$, $S_{i,p}^{1,-} = \sqrt{2S} a_{i,p}^{\dagger}$, $S_{i,p}^{2,z} = b_{i,p}^{\dagger} b_{i,p} - S$, $S_{i,p}^{2,+} = \sqrt{2S} b_{i,p}^{\dagger}$, $S_{i,p}^{2,-} = \sqrt{2S} b_{i,p}$, and the Fourier transformation $a_{i,p} = \frac{1}{\sqrt{N}}\sum_{\mathbf{k}} e^{i\mathbf{k}\cdot\mathbf{R}_i} a_{\mathbf{k},p}$, $a_{i,p}^{\dagger} = \frac{1}{\sqrt{N}}\sum_{\mathbf{k}} e^{-i\mathbf{k}\cdot\mathbf{R}_i} a_{\mathbf{k},p}^{\dagger}$, $b_{i,p} = \frac{1}{\sqrt{N}}\sum_{\mathbf{k}} e^{-i\mathbf{k}\cdot\mathbf{R}_i} b_{\mathbf{k},p}$, $b_{i,p}^{\dagger} = \frac{1}{\sqrt{N}}\sum_{\mathbf{k}} e^{i\mathbf{k}\cdot\mathbf{R}_i} b_{\mathbf{k},p}^{\dagger}$, we obtain

$$H_1^1 \approx -\sum_{\mathbf{k}} \Big\{ S J_{1,2}^1 \left[a_{\mathbf{k},1}^{\dagger} a_{\mathbf{k},1} + a_{\mathbf{k},2}^{\dagger} a_{\mathbf{k},2} - a_{\mathbf{k},1}^{\dagger} a_{\mathbf{k},2} - a_{\mathbf{k},2}^{\dagger} a_{\mathbf{k},1} \right]$$

$$+ \ SJ^{1}_{1,5}\left[a^{\dagger}_{\mathbf{k},1}a_{\mathbf{k},1}+a^{\dagger}_{\mathbf{k},5}a_{\mathbf{k},5}-a^{\dagger}_{\mathbf{k},1}a_{\mathbf{k},5}-a^{\dagger}_{\mathbf{k},5}a_{\mathbf{k},1}\right]$$
$$+ \ SJ^{1}_{1,6}\left[a^{\dagger}_{\mathbf{k},1}a_{\mathbf{k},1}+a^{\dagger}_{\mathbf{k},6}a_{\mathbf{k},6}-a^{\dagger}_{\mathbf{k},1}a_{\mathbf{k},6}-a^{\dagger}_{\mathbf{k},6}a_{\mathbf{k},1}\right]$$
$$+ \ SJ^{1}_{1,4}\left[a^{\dagger}_{\mathbf{k},1}a_{\mathbf{k},1}+a^{\dagger}_{\mathbf{k},4}a_{\mathbf{k},4}-e^{i\mathbf{k}\cdot\delta_2}a^{\dagger}_{\mathbf{k},1}a_{\mathbf{k},4}-e^{-i\mathbf{k}\cdot\delta_2}a^{\dagger}_{\mathbf{k},4}a_{\mathbf{k},1}\right]$$
$$+ \ SJ^{1}_{1,8}\left[a^{\dagger}_{\mathbf{k},1}a_{\mathbf{k},1}+a^{\dagger}_{\mathbf{k},8}a_{\mathbf{k},8}-e^{i\mathbf{k}\cdot\delta_2}a^{\dagger}_{\mathbf{k},1}a_{\mathbf{k},8}-e^{-i\mathbf{k}\cdot\delta_2}a^{\dagger}_{\mathbf{k},8}a_{\mathbf{k},1}\right]$$
$$+ \ SJ^{1}_{1,21}\left[a^{\dagger}_{\mathbf{k},1}a_{\mathbf{k},1}+a^{\dagger}_{\mathbf{k},21}a_{\mathbf{k},21}-e^{i\mathbf{k}\cdot\delta_4}a^{\dagger}_{\mathbf{k},1}a_{\mathbf{k},21}-e^{-i\mathbf{k}\cdot\delta_4}a^{\dagger}_{\mathbf{k},21}a_{\mathbf{k},1}\right]$$
$$+ \ SJ^{1}_{1,22}\left[a^{\dagger}_{\mathbf{k},1}a_{\mathbf{k},1}+a^{\dagger}_{\mathbf{k},22}a_{\mathbf{k},22}-e^{i\mathbf{k}\cdot\delta_4}a^{\dagger}_{\mathbf{k},1}a_{\mathbf{k},22}-e^{-i\mathbf{k}\cdot\delta_4}a^{\dagger}_{\mathbf{k},22}a_{\mathbf{k},1}\right]$$
$$+ \ SJ^{1}_{1,24}\left[a^{\dagger}_{\mathbf{k},1}a_{\mathbf{k},1}+a^{\dagger}_{\mathbf{k},24}a_{\mathbf{k},24}-e^{i\mathbf{k}\cdot\delta_6}a^{\dagger}_{\mathbf{k},1}a_{\mathbf{k},24}-e^{-i\mathbf{k}\cdot\delta_6}a^{\dagger}_{\mathbf{k},24}a_{\mathbf{k},1}\right]\Big\} \tag{4}$$

and

$$H^{12}_{1} \approx -\sum_{\mathbf{k}}\Big\{SJ^{12}_{1,1}\left[-a^{\dagger}_{\mathbf{k},1}a_{\mathbf{k},1}-b^{\dagger}_{\mathbf{k},1}b_{\mathbf{k},1}-a_{\mathbf{k},1}b_{\mathbf{k},1}-b^{\dagger}_{\mathbf{k},1}a^{\dagger}_{\mathbf{k},1}\right]$$
$$+ \ SJ^{12}_{1,5}\left[-a^{\dagger}_{\mathbf{k},1}a_{\mathbf{k},1}-b^{\dagger}_{\mathbf{k},5}b_{\mathbf{k},5}-a_{\mathbf{k},1}b_{\mathbf{k},5}-b^{\dagger}_{\mathbf{k},5}a^{\dagger}_{\mathbf{k},1}\right]$$
$$+ \ SJ^{12}_{1,9}\left[-a^{\dagger}_{\mathbf{k},1}a_{\mathbf{k},1}-b^{\dagger}_{\mathbf{k},9}b_{\mathbf{k},9}-a_{\mathbf{k},1}b_{\mathbf{k},9}-b^{\dagger}_{\mathbf{k},9}a^{\dagger}_{\mathbf{k},1}\right]$$
$$+ \ SJ^{12}_{1,2}\left[-a^{\dagger}_{\mathbf{k},1}a_{\mathbf{k},1}-b^{\dagger}_{\mathbf{k},2}b_{\mathbf{k},2}-a_{\mathbf{k},1}b_{\mathbf{k},2}-b^{\dagger}_{\mathbf{k},2}a^{\dagger}_{\mathbf{k},1}\right]$$
$$+ \ SJ^{12}_{1,6}\left[-a^{\dagger}_{\mathbf{k},1}a_{\mathbf{k},1}-b^{\dagger}_{\mathbf{k},6}b_{\mathbf{k},6}-a_{\mathbf{k},1}b_{\mathbf{k},6}-b^{\dagger}_{\mathbf{k},6}a^{\dagger}_{\mathbf{k},1}\right]$$
$$+ \ SJ^{12}_{1,10}\left[-a^{\dagger}_{\mathbf{k},1}a_{\mathbf{k},1}-b^{\dagger}_{\mathbf{k},10}b_{\mathbf{k},10}-a_{\mathbf{k},1}b_{\mathbf{k},10}-b^{\dagger}_{\mathbf{k},10}a^{\dagger}_{\mathbf{k},1}\right]\Big\}, \tag{5}$$

where the constant and high order interaction terms have been neglected. For CrI_3, we also employ Cr1-1 as an example (see the specific positions in Fig. S5), and the Hamiltonian contributed by the intralyer exchange couplings reads

$$H^{1}_{1} = \sum_{i} J^{1}_{1,2}\boldsymbol{S}^{1}_{i,1}\cdot\boldsymbol{S}^{1}_{i,2} + J^{1}_{1,3}\boldsymbol{S}^{1}_{i,1}\cdot\boldsymbol{S}^{1}_{i-a,3} + J^{1}_{1,4}\boldsymbol{S}^{1}_{i,1}\cdot\boldsymbol{S}^{1}_{i-a+b,4} + J^{1}_{1,5}\boldsymbol{S}^{1}_{i,1}\cdot\boldsymbol{S}^{1}_{i,5} + J^{1}_{1,6}\boldsymbol{S}^{1}_{i,1}\cdot\boldsymbol{S}^{1}_{i,6}$$
$$+ \ J^{1}_{1,7}\boldsymbol{S}^{1}_{i,1}\cdot\boldsymbol{S}^{1}_{i-a,7} + J^{1}_{1,8}\boldsymbol{S}^{1}_{i,1}\cdot\boldsymbol{S}^{1}_{i,8} + J^{1}_{1,12}\boldsymbol{S}^{1}_{i,1}\cdot\boldsymbol{S}^{1}_{i,12} + J^{1}_{1,14}\boldsymbol{S}^{1}_{i,1}\cdot\boldsymbol{S}^{1}_{i-a,14}$$
$$\approx -\sum_{\mathbf{k}}\Big\{SJ^{1}_{1,2}\left[a^{\dagger}_{\mathbf{k},1}a_{\mathbf{k},1}+a^{\dagger}_{\mathbf{k},2}a_{\mathbf{k},2}-a^{\dagger}_{\mathbf{k},1}a_{\mathbf{k},2}-a^{\dagger}_{\mathbf{k},2}a_{\mathbf{k},1}\right]$$
$$+ \ SJ^{1}_{1,3}\left[a^{\dagger}_{\mathbf{k},1}a_{\mathbf{k},1}+a^{\dagger}_{\mathbf{k},3}a_{\mathbf{k},3}-e^{i\mathbf{k}\cdot\delta_2}a^{\dagger}_{\mathbf{k},1}a_{\mathbf{k},3}-e^{-i\mathbf{k}\cdot\delta_2}a^{\dagger}_{\mathbf{k},3}a_{\mathbf{k},1}\right]$$
$$+ \ SJ^{1}_{1,4}\left[a^{\dagger}_{\mathbf{k},1}a_{\mathbf{k},1}+a^{\dagger}_{\mathbf{k},4}a_{\mathbf{k},4}-e^{i\mathbf{k}\cdot\delta_8}a^{\dagger}_{\mathbf{k},1}a_{\mathbf{k},4}-e^{-i\mathbf{k}\cdot\delta_8}a^{\dagger}_{\mathbf{k},4}a_{\mathbf{k},1}\right]$$
$$+ \ SJ^{1}_{1,5}\left[a^{\dagger}_{\mathbf{k},1}a_{\mathbf{k},1}+a^{\dagger}_{\mathbf{k},5}a_{\mathbf{k},5}-a^{\dagger}_{\mathbf{k},1}a_{\mathbf{k},5}-a^{\dagger}_{\mathbf{k},5}a_{\mathbf{k},1}\right]$$
$$+ \ SJ^{1}_{1,6}\left[a^{\dagger}_{\mathbf{k},1}a_{\mathbf{k},1}+a^{\dagger}_{\mathbf{k},6}a_{\mathbf{k},6}-a^{\dagger}_{\mathbf{k},1}a_{\mathbf{k},6}-a^{\dagger}_{\mathbf{k},6}a_{\mathbf{k},1}\right]$$
$$+ \ SJ^{1}_{1,7}\left[a^{\dagger}_{\mathbf{k},1}a_{\mathbf{k},1}+a^{\dagger}_{\mathbf{k},7}a_{\mathbf{k},7}-e^{i\mathbf{k}\cdot\delta_2}a^{\dagger}_{\mathbf{k},1}a_{\mathbf{k},7}-e^{-i\mathbf{k}\cdot\delta_2}a^{\dagger}_{\mathbf{k},7}a_{\mathbf{k},1}\right]$$
$$+ \ SJ^{1}_{1,8}\left[a^{\dagger}_{\mathbf{k},1}a_{\mathbf{k},1}+a^{\dagger}_{\mathbf{k},8}a_{\mathbf{k},8}-a^{\dagger}_{\mathbf{k},1}a_{\mathbf{k},8}-a^{\dagger}_{\mathbf{k},8}a_{\mathbf{k},1}\right]$$

$$+ \; SJ^{1}_{1,12}\left[a^{\dagger}_{\mathbf{k},1}a_{\mathbf{k},1} + a^{\dagger}_{\mathbf{k},12}a_{\mathbf{k},12} - a^{\dagger}_{\mathbf{k},1}a_{\mathbf{k},12} - a^{\dagger}_{\mathbf{k},12}a_{\mathbf{k},1}\right]$$
$$+ \; SJ^{1}_{1,14}\left[a^{\dagger}_{\mathbf{k},1}a_{\mathbf{k},1} + a^{\dagger}_{\mathbf{k},14}a_{\mathbf{k},14} - e^{i\mathbf{k}\cdot\delta_2}a^{\dagger}_{\mathbf{k},1}a_{\mathbf{k},14} - e^{-i\mathbf{k}\cdot\delta_2}a^{\dagger}_{\mathbf{k},14}a_{\mathbf{k},1}\right]\Bigg\} \tag{6}$$

The Hamiltonian contributed by the interlayer exchange couplings reads

$$H^{12}_{1} = \sum_{i} J^{12}_{1,2}\boldsymbol{S}^{1}_{i,1}\cdot\boldsymbol{S}^{2}_{i,2} + J^{12}_{1,3}\boldsymbol{S}^{1}_{i,1}\cdot\boldsymbol{S}^{2}_{i,3} + J^{12}_{1,7}\boldsymbol{S}^{1}_{i,1}\cdot\boldsymbol{S}^{2}_{i-a,7} + J^{12}_{1,9}\boldsymbol{S}^{1}_{i,1}\cdot\boldsymbol{S}^{2}_{i,9} + J^{12}_{1,10}\boldsymbol{S}^{1}_{i,1}\cdot\boldsymbol{S}^{2}_{i,10}$$
$$\approx -\sum_{\mathbf{k}}\Bigg\{SJ^{12}_{1,2}\left[-a^{\dagger}_{\mathbf{k},1}a_{\mathbf{k},1} - b^{\dagger}_{\mathbf{k},2}b_{\mathbf{k},2} - a_{\mathbf{k},1}b_{\mathbf{k},2} - b^{\dagger}_{\mathbf{k},2}a^{\dagger}_{\mathbf{k},1}\right]$$
$$+ \; SJ^{12}_{1,3}\left[-a^{\dagger}_{\mathbf{k},1}a_{\mathbf{k},1} - b^{\dagger}_{\mathbf{k},3}b_{\mathbf{k},3} - a_{\mathbf{k},1}b_{\mathbf{k},3} - b^{\dagger}_{\mathbf{k},3}a^{\dagger}_{\mathbf{k},1}\right]$$
$$+ \; SJ^{12}_{1,7}\left[-a^{\dagger}_{\mathbf{k},1}a_{\mathbf{k},1} - b^{\dagger}_{\mathbf{k},7}b_{\mathbf{k},7} - e^{-i\mathbf{k}\cdot\delta_1}a_{\mathbf{k},1}b_{\mathbf{k},7} - e^{i\mathbf{k}\cdot\delta_1}b^{\dagger}_{\mathbf{k},7}a^{\dagger}_{\mathbf{k},1}\right]$$
$$+ \; SJ^{12}_{1,9}\left[-a^{\dagger}_{\mathbf{k},1}a_{\mathbf{k},1} - b^{\dagger}_{\mathbf{k},9}b_{\mathbf{k},9} - a_{\mathbf{k},1}b_{\mathbf{k},9} - b^{\dagger}_{\mathbf{k},9}a^{\dagger}_{\mathbf{k},1}\right]$$
$$+ \; SJ^{12}_{1,10}\left[-a^{\dagger}_{\mathbf{k},1}a_{\mathbf{k},1} - b^{\dagger}_{\mathbf{k},10}b_{\mathbf{k},10} - a_{\mathbf{k},1}b_{\mathbf{k},10} - b^{\dagger}_{\mathbf{k},10}a^{\dagger}_{\mathbf{k},1}\right]\Bigg\} \tag{7}$$

By applying the aforementioned methods to other magnetic atoms, one can derive a comprehensive spin Hamiltonian in reciprocal space that fully characterizes the magnetic interactions within the entire twisted antiferromagnetic system. The same procedure, involving the Holstein–Primakoff transformation and the Fourier transform, is applied to handle the magnetic anisotropy term

$$K\sum_{\alpha=1,2}\sum_{p=1}^{24}\sum_{i=1}^{N}(S^{\alpha,z}_{i,p})^2 = K\sum_{p=1}^{24}\sum_{i=1}^{N}\left[(S - a^{\dagger}_{i,p}a_{i,p})^2 + (b^{\dagger}_{i,p}b_{i,p} - S)^2\right]$$
$$\approx -2KS\sum_{l=1}^{24}\sum_{i=1}^{N}\left[a^{\dagger}_{i,p}a_{i,p} + b^{\dagger}_{i,p}b_{i,p}\right]$$
$$= -2KS\sum_{l=1}^{24}\sum_{\mathbf{k}}\left[a^{\dagger}_{\mathbf{k},p}a_{\mathbf{k},p} + b^{\dagger}_{\mathbf{k},p}b_{\mathbf{k},p}\right] \tag{8}$$

III. *Magnonic dispersions*—The bosonic Hamiltonian can be written as:

$$H = \sum_{\mathbf{k}} \left[a_{\mathbf{k}}^{\dagger} \; b_{\mathbf{k}} \right] \cdot H_{\mathbf{k}} \cdot \begin{bmatrix} a_{\mathbf{k}} \\ b_{\mathbf{k}}^{\dagger} \end{bmatrix}, \tag{9}$$

where $a_{\mathbf{k}}^{\dagger} \equiv \left[a_{\mathbf{k},1}^{\dagger}, \cdots, a_{\mathbf{k},M}^{\dagger}\right]$ and $b_{\mathbf{k}}^{\dagger} \equiv \left[b_{\mathbf{k},1}^{\dagger}, \cdots, b_{\mathbf{k},M}^{\dagger}\right]^{T}$ represent the magnonic creation operators, and $H_{\mathbf{k}}$ is a $2M \times 2M$ Hermitian matrix with $M = 24$ and $M = 14$ for twisted bilayer $CrPS_4$ and CrI_3 respectively. All the nonzero elements, obtained by methods introduced in section II, are given in the *Appendix A* and *B*. The Hermitian matrix $H_{\mathbf{k}}$ can be written as:

$$H_{\mathbf{k}} = S \begin{bmatrix} A_{\mathbf{k}} & C_{\mathbf{k}} \\ C_{\mathbf{k}}^{\dagger} & B_{\mathbf{k}} \end{bmatrix}, \tag{10}$$

where $S = \frac{3}{2}$ is the spin quantum value for Cr atoms. The non-zero elements of $M \times M$ matrix $A_{\mathbf{k}}$, matrix $B_{\mathbf{k}}$ and matrix $C_{\mathbf{k}}$ are deduced from the DFT-resolved spin Hamiltonian. The bosonic Hamiltonian $H_{\mathbf{k}}$ is then diagonalized by a paraunitary matrix $T_{\mathbf{k}}$ rather than a unitary matrix [14],

$$T_{\mathbf{k}}^{\dagger} H_{\mathbf{k}} T_{\mathbf{k}} = E_{\mathbf{k}} = diag\{E_{\mathbf{k}}(1), \ldots, E_{\mathbf{k}}(2M)\} \tag{11}$$

with $\left[\alpha_{\mathbf{k}}^{\dagger}, \beta_{\mathbf{k}}\right] T_{\mathbf{k}}^{\dagger} = \left[a_{\mathbf{k}}^{\dagger}, b_{\mathbf{k}}\right]$, and $E_{\mathbf{k}}$ is a positive-definite $2M \times 2M$ diagonal matrix. The diagonal element of $E_{\mathbf{k}}$ gives the magnonic dispersions. For guaranteeing that $\alpha_{\mathbf{k}}^{\dagger}$ and $\beta_{\mathbf{k}}$ keep the boson statistics, the paraunitary matrix $T_{\mathbf{k}}$ should satisfy $T_{\mathbf{k}}^{\dagger} \tau_3 T_{\mathbf{k}} = \tau_3$, where τ_3 is a $2M \times 2M$ diagonal matrix with $[\tau_3]_{jj} = 1$ for $j = 1, \ldots, M$ and $[\tau_3]_{jj} = -1$ for $j = M+1, \ldots, 2M$. Given that a $2M$-square Hermitian matrix $H_{\mathbf{k}}$ is positive definite, it can be diagonalized by a paraunitary transformation into a diagonal matrix with all positive elements. Furthermore, the paraunitary matrix required for this diagonalization can be obtained through the application of the Cholesky decomposition [14]. For finding $T_{\mathbf{k}}$, we first apply the Cholesky decomposition, $H_{\mathbf{k}} = \Gamma_{\mathbf{k}}^{\dagger} \Gamma_{\mathbf{k}}$, where $\Gamma_{\mathbf{k}}$ is a non-singular upper triangle matrix. We next diagonalizes the hermitian matrix $Q_{\mathbf{k}} = \Gamma_{\mathbf{k}} \tau_3 \Gamma_{\mathbf{k}}^{\dagger}$ by using a unitary matrix $U_{\mathbf{k}}$,

$$U_{\mathbf{k}}^{\dagger} Q_{\mathbf{k}} U_{\mathbf{k}} = \Lambda_{\mathbf{k}} = diag\{E_{\mathbf{k}}(1), \ldots, E_{\mathbf{k}}(M), -E_{\mathbf{k}}(M+1), \ldots, -E_{\mathbf{k}}(2M)\} \tag{12}$$

, and $E_{\mathbf{k}} \equiv \tau_3 \Lambda_{\mathbf{k}}$. Finally, we obtain the paraunitary matrix[14], i.e., $T_{\mathbf{k}} = \Gamma_{\mathbf{k}}^{-1} U_{\mathbf{k}} E_{\mathbf{k}}^{1/2}$.

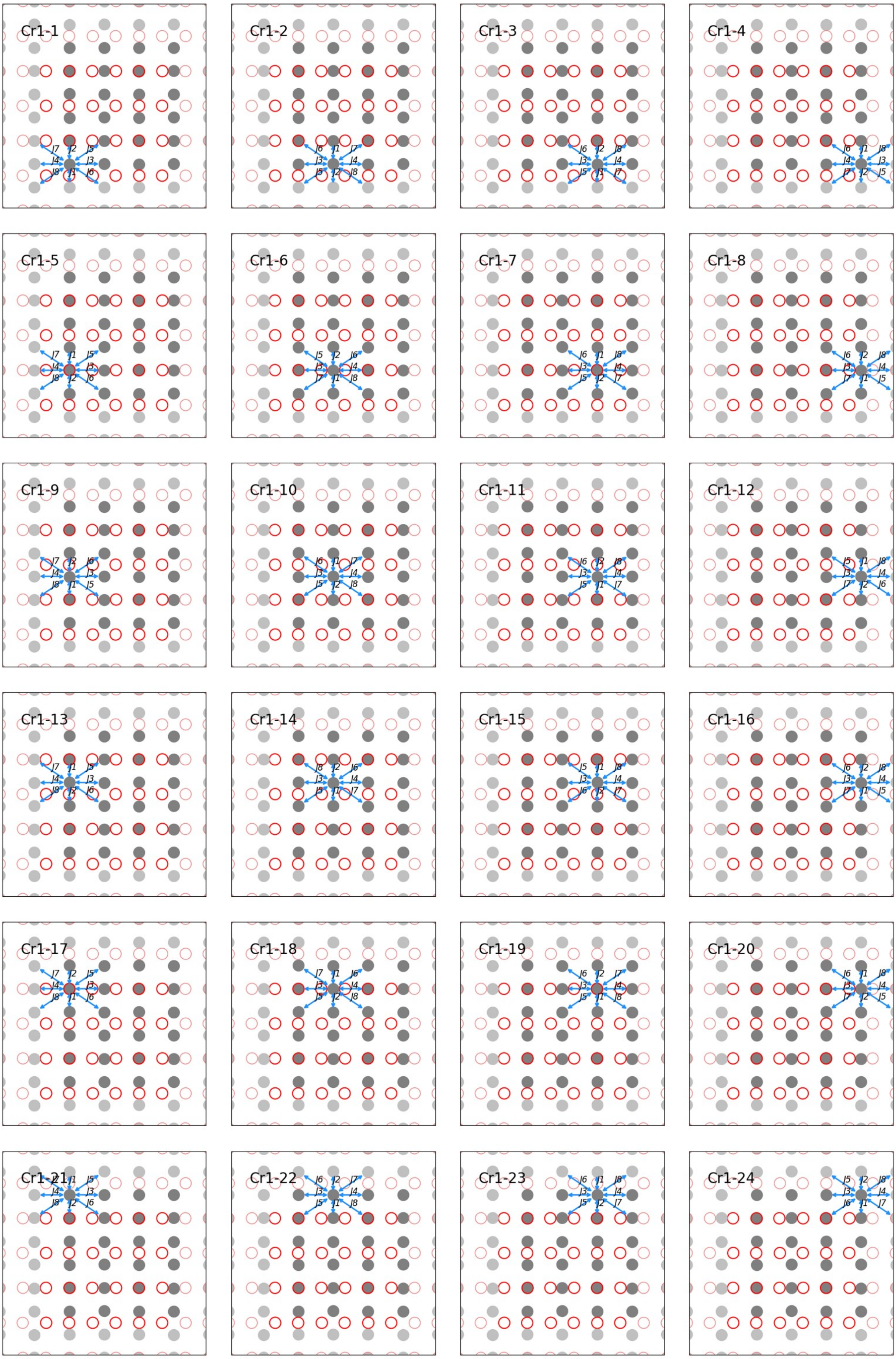

Figure S1. Atom-resolved intralayer Heisenberg exchange couplings for the top layer in orthogonally twisted bilayer $CrPS_4$.

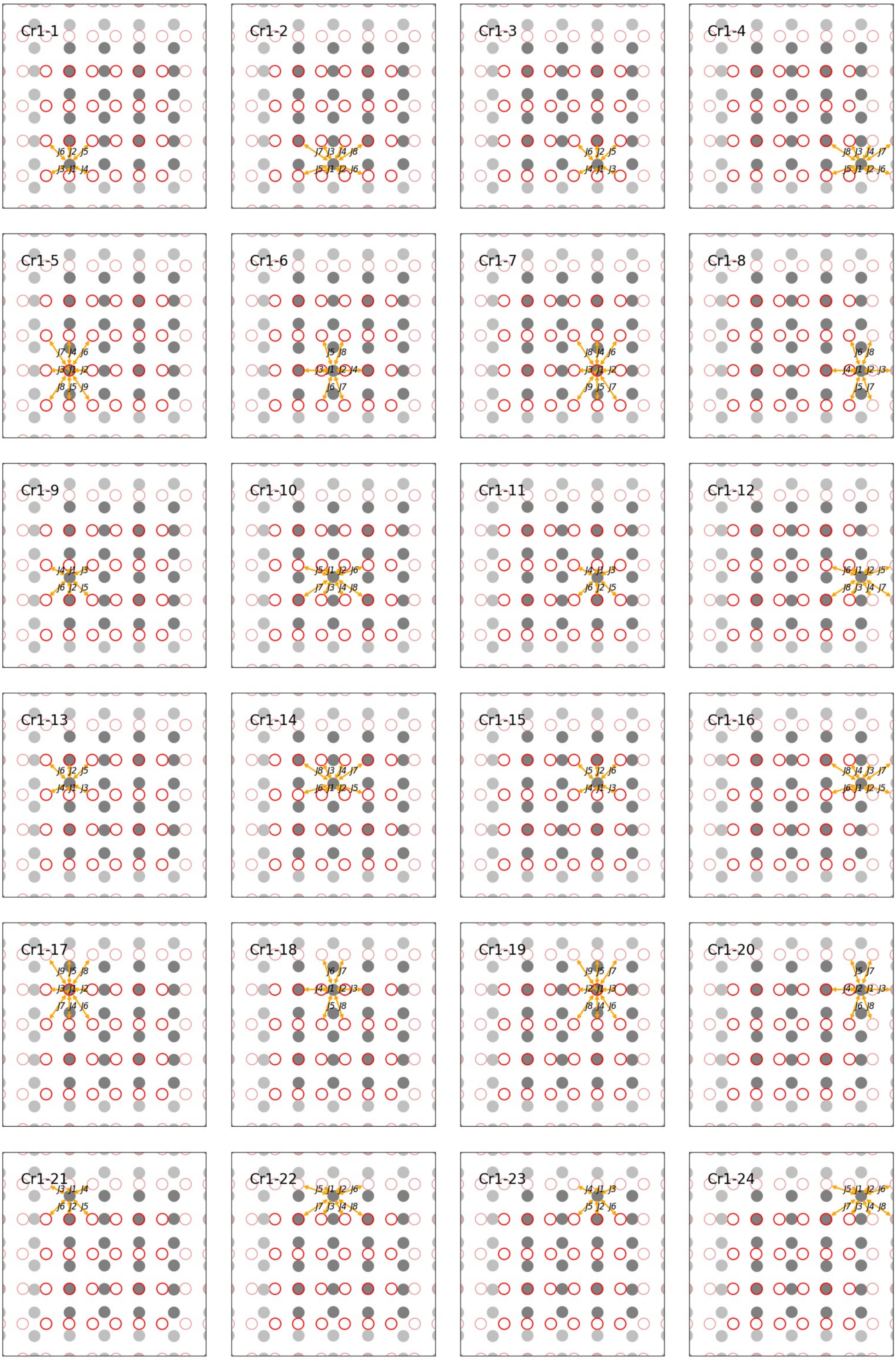

Figure S2. Atom-resolved interlayer Heisenberg exchange couplings for the top layer in orthogonally twisted bilayer $CrPS_4$.

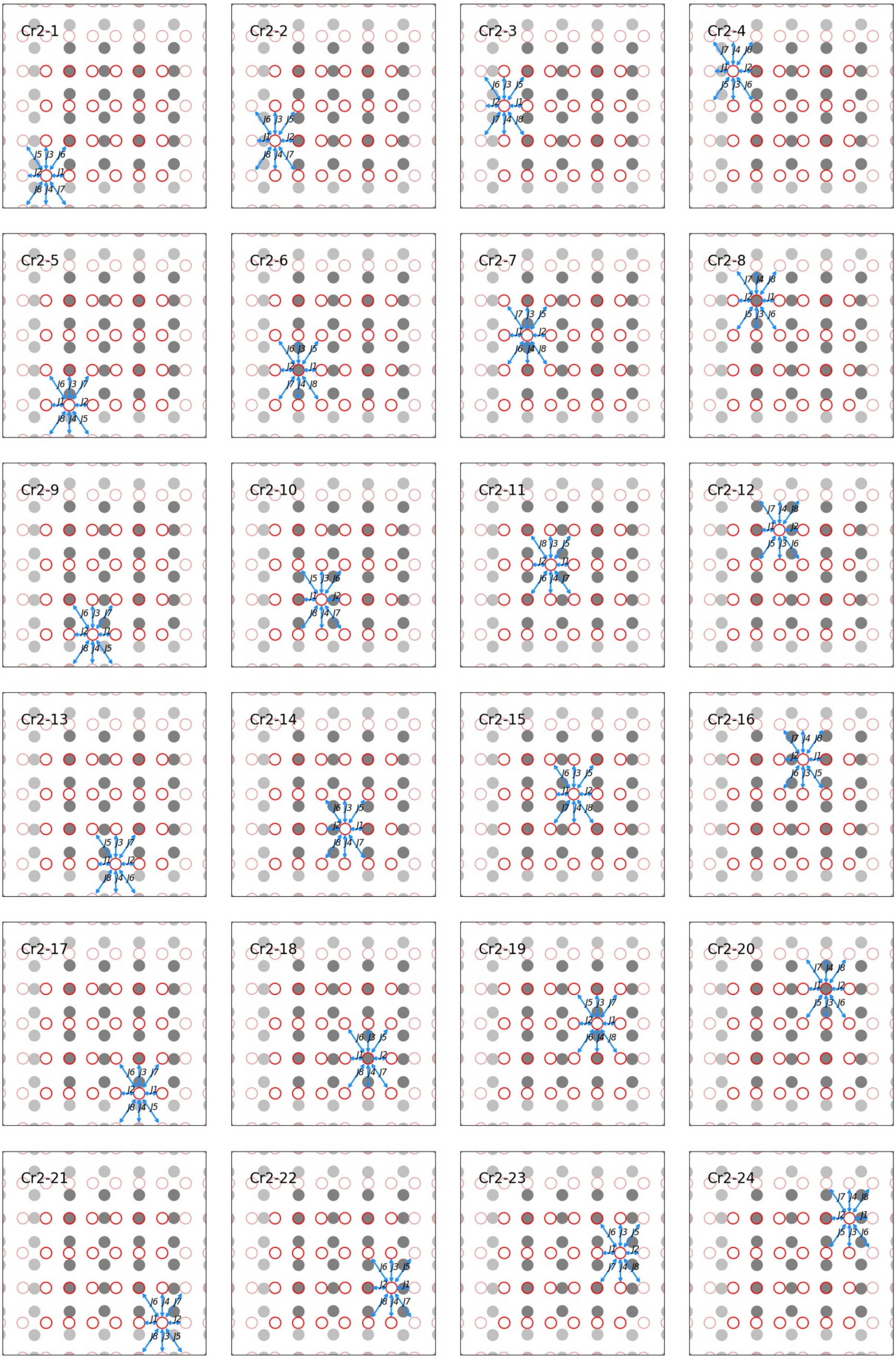

Figure S3. Atom-resolved intralayer Heisenberg exchange couplings for the bottom layer in orthogonally twisted bilayer $CrPS_4$.

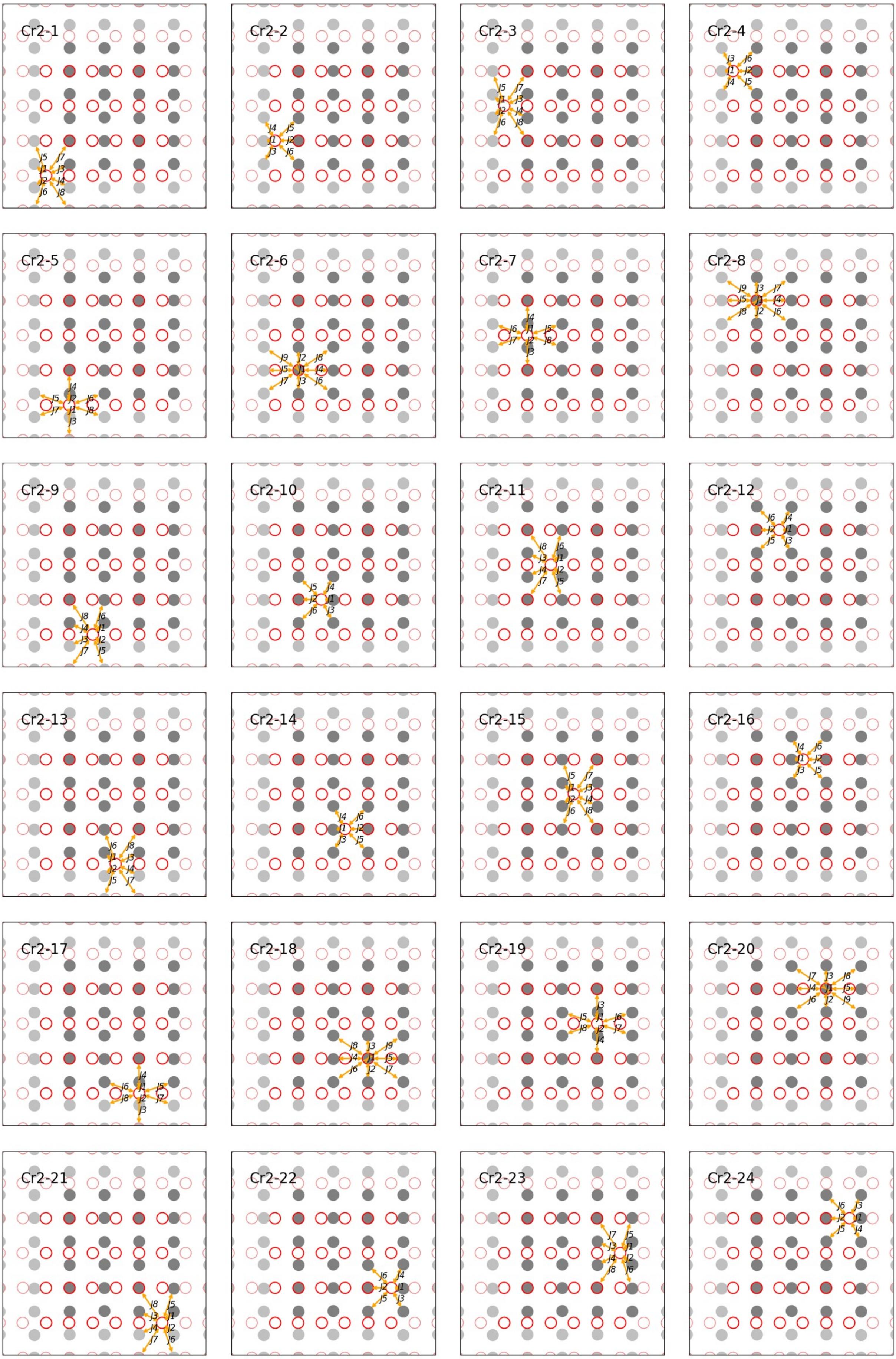

Figure S4. Atom-resolved interlayer Heisenberg exchange couplings for the bottom layer in orthogonally twisted bilayer $CrPS_4$.

Table S1 The DFT-resolved magnitudes (meV) of atomistic intralayer exchange couplings in twisted bilayer $CrPS_4$.

Cr	J_1	J_2	J_3	J_4	J_5	J_6	J_7	J_8
1-1	5.0393	3.5552	0.4863	0.4809	0.4974	0.4920	0.4838	0.6503
1-2	5.0380	3.6183	0.4863	0.4932	0.5093	0.4593	0.4706	0.4268
1-3	5.0094	3.5265	0.4932	0.4548	0.6286	0.5216	0.5097	0.3901
1-4	5.0498	3.5999	0.4809	0.4548	0.4334	0.5406	0.5029	0.4933
1-5	5.1436	3.5552	0.4751	0.5004	0.5144	0.4593	0.6112	0.4933
1-6	5.0380	3.5764	0.4751	0.4732	0.4677	0.4991	0.4974	0.5216
1-7	5.1851	3.5265	0.4732	0.4906	0.4706	0.6534	0.5406	0.5133
1-8	5.0498	3.5689	0.4906	0.5004	0.4838	0.4996	0.3901	0.5717
1-9	5.1436	3.4981	0.5119	0.5233	0.4677	0.5709	0.5086	0.5717
1-10	5.1371	3.5764	0.5119	0.5398	0.5144	0.4635	0.4907	0.6534
1-11	5.1851	3.4627	0.5398	0.4857	0.4991	0.4967	0.4996	0.5338
1-12	5.1098	3.5689	0.4857	0.5233	0.4497	0.6112	0.5133	0.4845
1-13	5.1262	3.4981	0.4605	0.4796	0.4889	0.4635	0.4750	0.4845
1-14	5.1371	3.5342	0.4605	0.4789	0.5709	0.4047	0.4967	0.5090
1-15	5.1243	3.4627	0.4789	0.4706	0.5488	0.4907	0.4497	0.4814
1-16	5.1098	3.5489	0.4706	0.4796	0.5086	0.4756	0.5338	0.4531
1-17	5.1262	3.6299	0.4905	0.4739	0.5453	0.5090	0.4796	0.4531
1-18	5.0586	3.5342	0.4905	0.5126	0.4889	0.5004	0.5127	0.5488
1-19	5.1243	3.6407	0.5126	0.4634	0.4047	0.5484	0.4423	0.4756
1-20	5.0259	3.5489	0.4634	0.4739	0.4750	0.6268	0.4814	0.5450
1-21	5.0393	3.6299	0.4927	0.5471	0.5093	0.5127	0.4334	0.5450
1-22	5.0586	3.6183	0.4927	0.5415	0.5453	0.4920	0.6286	0.5484
1-23	5.0094	3.6407	0.5415	0.5043	0.5004	0.4268	0.6268	0.5029
1-24	5.0259	3.5999	0.5043	0.5471	0.5097	0.4423	0.4796	0.6503
2-1	5.0259	3.5999	0.5043	0.5470	0.5098	0.4423	0.4796	0.6503
2-2	5.0094	3.6407	0.5414	0.5043	0.5004	0.4269	0.6268	0.5029
2-3	5.0586	3.6183	0.4927	0.5414	0.5453	0.4920	0.6286	0.5484
2-4	5.0393	3.6299	0.4927	0.5470	0.5092	0.5127	0.4335	0.5450
2-5	5.0259	3.5489	0.4634	0.4739	0.4750	0.6268	0.4814	0.5450
2-6	5.1243	3.6407	0.5126	0.4634	0.4047	0.5484	0.4423	0.4757
2-7	5.0586	3.5342	0.4904	0.5126	0.4889	0.5004	0.5127	0.5488
2-8	5.1262	3.6299	0.4904	0.4739	0.5453	0.5091	0.4796	0.4531
2-9	5.1099	3.5489	0.4706	0.4795	0.5087	0.4757	0.5338	0.4531
2-10	5.1243	3.4627	0.4789	0.4706	0.5488	0.4907	0.4497	0.4814
2-11	5.1371	3.5342	0.4605	0.4789	0.5709	0.4047	0.4967	0.5091
2-12	5.1262	3.4981	0.4605	0.4795	0.4889	0.4635	0.4750	0.4845
2-13	5.1099	3.5689	0.4857	0.5233	0.4497	0.6112	0.5132	0.4845
2-14	5.1850	3.4627	0.5398	0.4857	0.4991	0.4967	0.4996	0.5338
2-15	5.1371	3.5763	0.5118	0.5398	0.5144	0.4635	0.4907	0.6535
2-16	5.1436	3.4981	0.5118	0.5233	0.4677	0.5709	0.5087	0.5716
2-17	5.0498	3.5689	0.4906	0.5003	0.4838	0.4996	0.3901	0.5716
2-18	5.1850	3.5265	0.4731	0.4906	0.4706	0.6535	0.5406	0.5132
2-19	5.0379	3.5763	0.4751	0.4731	0.4677	0.4991	0.4974	0.5216
2-20	5.1436	3.5552	0.4751	0.5003	0.5144	0.4593	0.6112	0.4933
2-21	5.0498	3.5999	0.4809	0.4548	0.4335	0.5406	0.5029	0.4933
2-22	5.0094	3.5265	0.4932	0.4548	0.6286	0.5216	0.5098	0.3901
2-23	5.0379	3.6183	0.4863	0.4932	0.5092	0.4593	0.4706	0.4269
2-24	5.0393	3.5552	0.4863	0.4809	0.4974	0.4920	0.4838	0.6503

Table S2 The DFT-resolved magnitudes (meV) of atomistic interlayer exchange couplings in twisted bilayer $CrPS_4$.

Cr	J_1^c	J_2^c	J_3^c	J_4^c	J_5^c	J_6^c	J_7^c	J_8^c	J_9^c
1-1	0.0035	−0.0307	−0.0031	−0.0279	0.0071	−0.0003			
1-2	−0.0046	−0.0053	−0.0036	0.0000	−0.0014	−0.0021	−0.0002	−0.0024	
1-3	0.0039	−0.0387	−0.0076	0.0025	0.0044	−0.0024			
1-4	−0.0044	−0.0035	−0.0076	−0.0308	−0.0030	−0.0004	−0.0023	−0.0005	
1-5	−0.0234	−0.0559	0.0004	0.0038	−0.0027	−0.0009	−0.0013	−0.0015	−0.0007
1-6	0.0017	−0.0984	−0.0002	0.0081	0.0054	0.0087	0.0006	−0.0023	
1-7	−0.0222	−0.0387	0.0023	0.0081	−0.0009	−0.0024	−0.0005	−0.0021	−0.0013
1-8	0.0039	−0.0189	0.0018	−0.0009	−0.0030	−0.0021	0.0008	−0.0015	
1-9	−0.0608	0.0030	0.0222	0.0037	−0.0015	−0.0045			
1-10	−0.0173	0.1176	−0.0004	0.0005	0.0001	−0.0023	−0.0007	−0.0021	
1-11	−0.0984	0.0023	0.0000	0.0005	−0.0024	−0.0026			
1-12	−0.0053	0.0623	0.0025	−0.0008	0.0043	0.0006	0.0047	−0.0013	
1-13	−0.0077	−0.1015	−0.0229	−0.0013	0.0190	−0.0020			
1-14	−0.0035	−0.0173	−0.0229	0.0222	0.0054	0.0054	−0.0009	−0.0005	
1-15	0.0017	−0.0559	−0.0036	−0.0004	−0.0015	0.0071			
1-16	−0.0046	−0.0066	0.0278	−0.0279	0.0030	0.0087	0.0009	−0.0007	
1-17	0.0278	−0.1015	−0.0019	−0.0060	0.0002	−0.0005	−0.0091	0.0009	0.0021
1-18	−0.0077	−0.0608	0.0038	−0.0060	0.0054	0.0030	0.0043	0.0001	
1-19	−0.0234	0.0030	−0.0307	−0.0002	0.0018	−0.0002	−0.0023	−0.0007	0.0047
1-20	−0.0376	0.0035	0.0002	−0.0027	−0.0004	−0.0014	0.0015	0.0014	
1-21	−0.0376	−0.0020	0.0031	0.0278	−0.0020	0.0053			
1-22	−0.0066	0.0623	−0.0013	0.0037	0.0014	−0.0015	−0.0091	−0.0013	
1-23	−0.0189	0.0004	−0.0308	−0.0008	−0.0045	−0.0003			
1-24	−0.0035	0.0593	−0.0031	0.0031	0.0008	0.0015	−0.0015	0.0021	
2-1	−0.0035	0.0593	−0.0031	0.0031	0.0008	0.0015	−0.0015	0.0021	
2-2	−0.0189	0.0004	−0.0308	−0.0008	−0.0045	−0.0003			
2-3	−0.0066	0.0623	−0.0013	0.0037	0.0014	−0.0015	−0.0091	−0.0013	
2-4	−0.0376	−0.0020	0.0031	0.0278	−0.0020	0.0053			
2-5	−0.0376	0.0035	0.0002	−0.0027	−0.0004	−0.0014	0.0015	0.0014	
2-6	−0.0234	0.0030	−0.0307	−0.0002	0.0018	−0.0002	−0.0023	−0.0007	0.0047
2-7	−0.0077	−0.0608	0.0038	−0.0060	0.0054	0.0030	0.0043	0.0001	
2-8	0.0278	−0.1015	−0.0020	−0.0060	0.0002	−0.0005	−0.0091	0.0009	0.0021
2-9	−0.0046	−0.0066	0.0278	−0.0279	0.0030	0.0087	0.0009	−0.0007	
2-10	0.0017	−0.0559	−0.0036	−0.0004	−0.0015	0.0071			
2-11	−0.0035	−0.0173	−0.0229	0.0222	0.0054	0.0054	−0.0009	−0.0005	
2-12	−0.0077	−0.1015	−0.0229	−0.0013	0.0190	−0.0020			
2-13	−0.0053	0.0623	0.0025	−0.0008	0.0043	0.0006	0.0047	−0.0013	
2-14	−0.0984	0.0023	0.0000	0.0005	−0.0024	−0.0026			
2-15	−0.0173	0.1176	−0.0004	0.0005	0.0001	−0.0023	−0.0007	−0.0021	
2-16	−0.0608	0.0030	0.0222	0.0037	−0.0015	−0.0045			
2-17	0.0039	−0.0189	0.0018	−0.0009	−0.0030	−0.0021	0.0008	−0.0015	
2-18	−0.0222	−0.0387	0.0023	0.0081	−0.0009	−0.0024	−0.0005	−0.0021	−0.0013
2-19	0.0017	−0.0984	−0.0002	0.0081	0.0054	0.0087	0.0006	−0.0023	
2-20	−0.0234	−0.0559	0.0004	0.0038	−0.0027	−0.0009	−0.0013	−0.0015	−0.0007
2-21	−0.0044	−0.0035	−0.0076	−0.0308	−0.0030	−0.0004	−0.0023	−0.0005	
2-22	0.0039	−0.0387	−0.0076	0.0025	0.0044	−0.0024			
2-23	−0.0046	−0.0053	−0.0036	0.0000	−0.0014	−0.0021	−0.0002	−0.0024	
2-24	0.0035	−0.0307	−0.0031	−0.0279	0.0071	−0.0003			

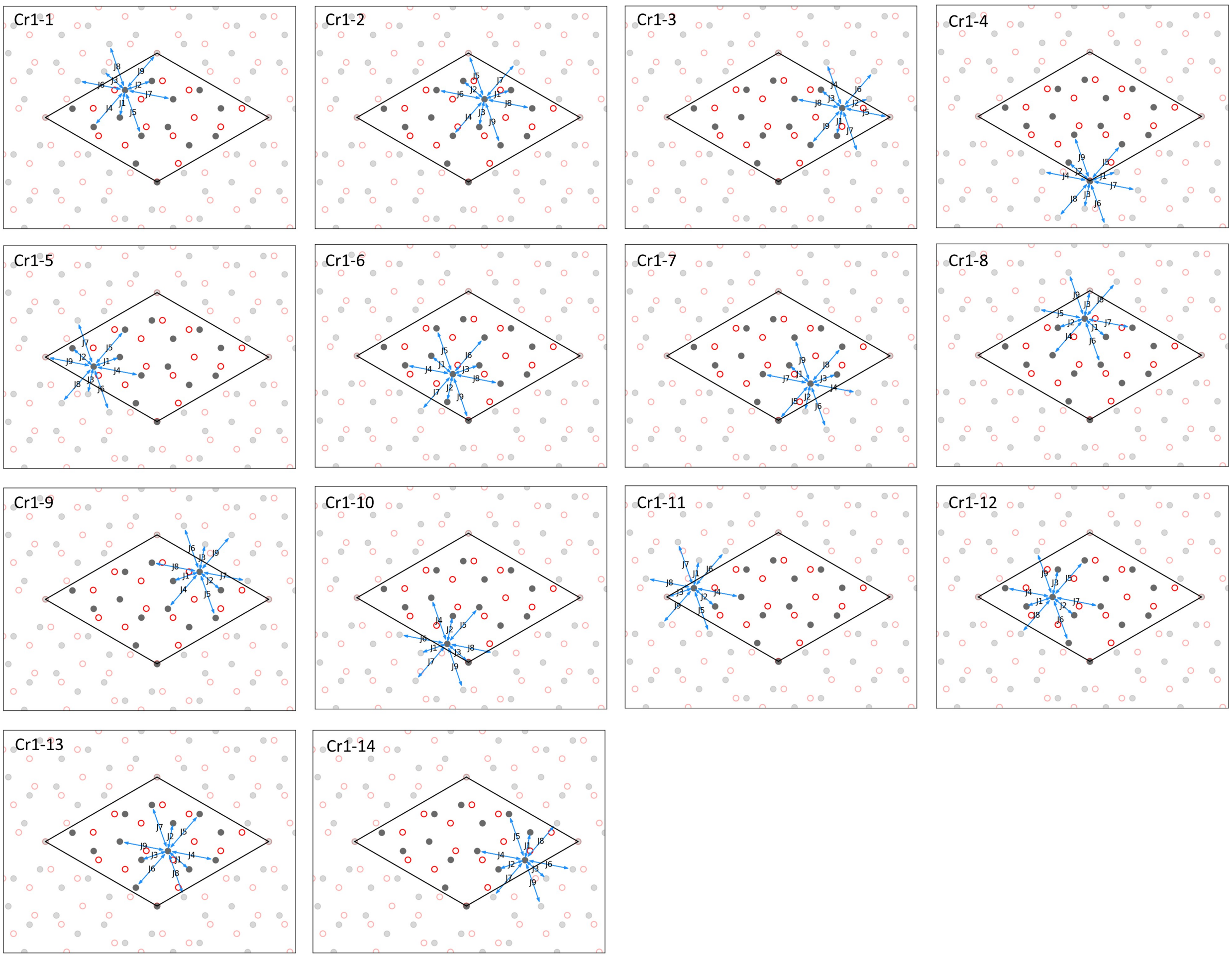

Figure S5. Atom-resolved intralayer Heisenberg exchange couplings for the top layer in twisted bilayer CrI_3.

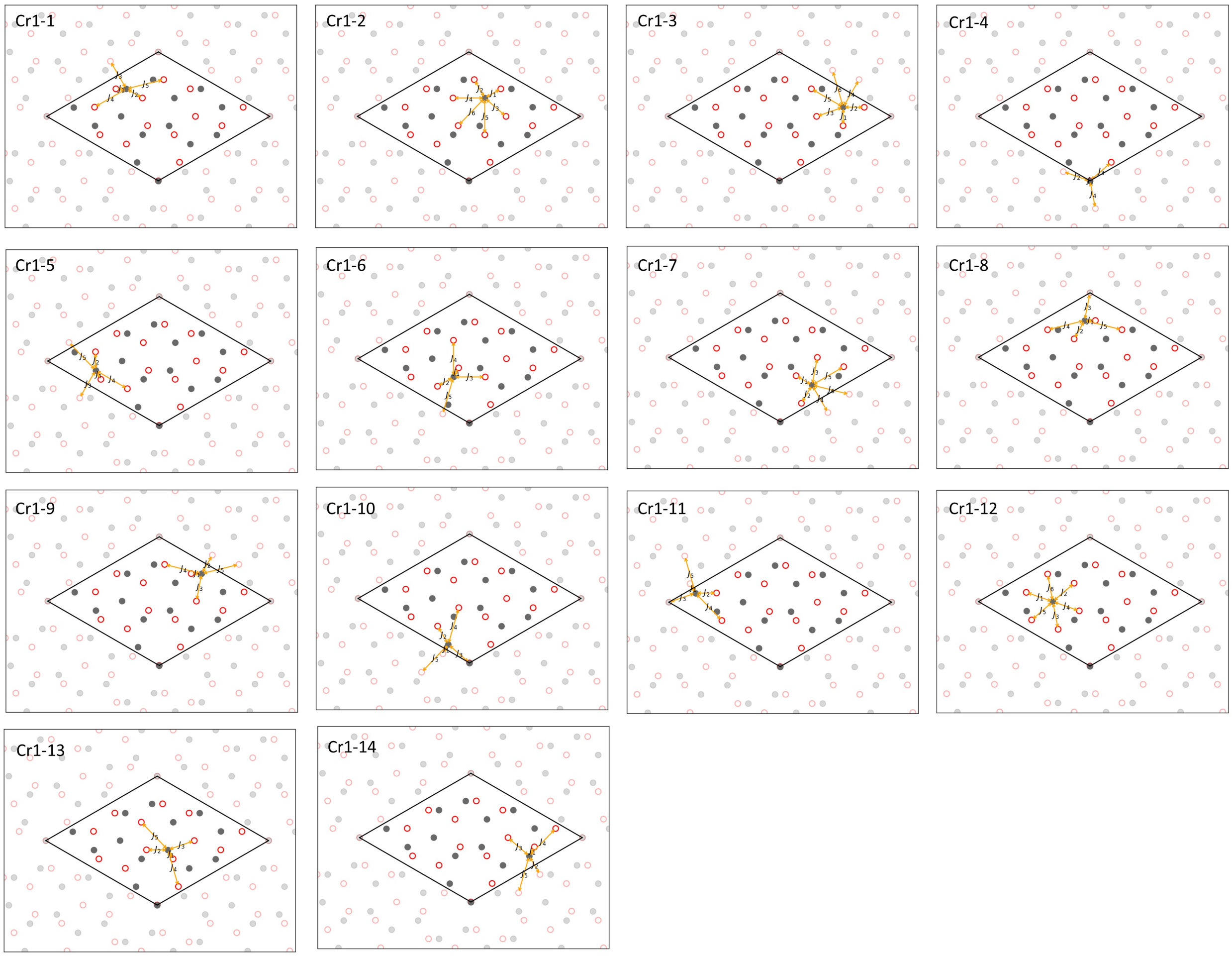

Figure S6. Atom-resolved interlayer Heisenberg exchange couplings for the top layer in twisted bilayer CrI_3.

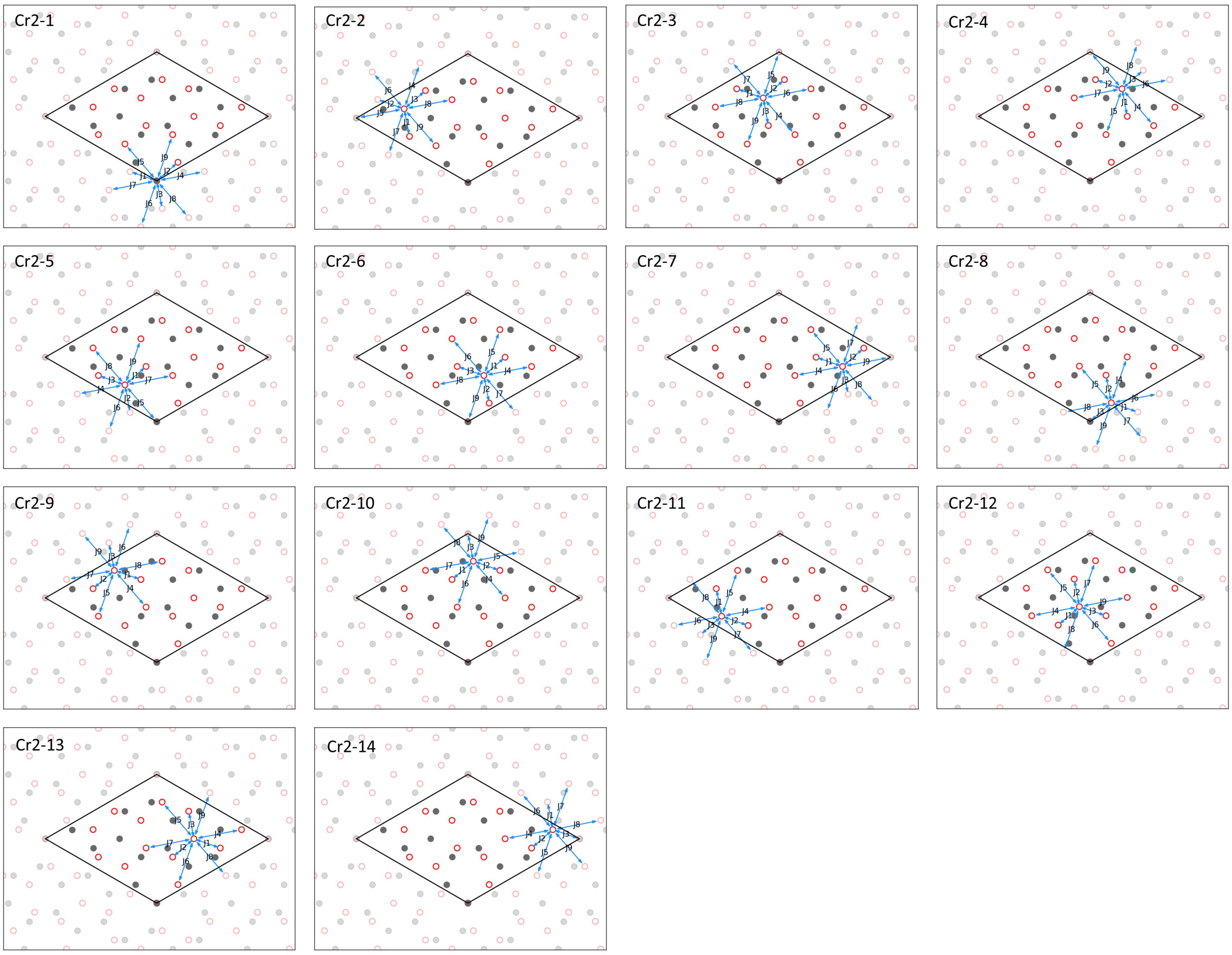

Figure S7. Atom-resolved intralayer Heisenberg exchange couplings for the bottom layer in twisted bilayer CrI_3.

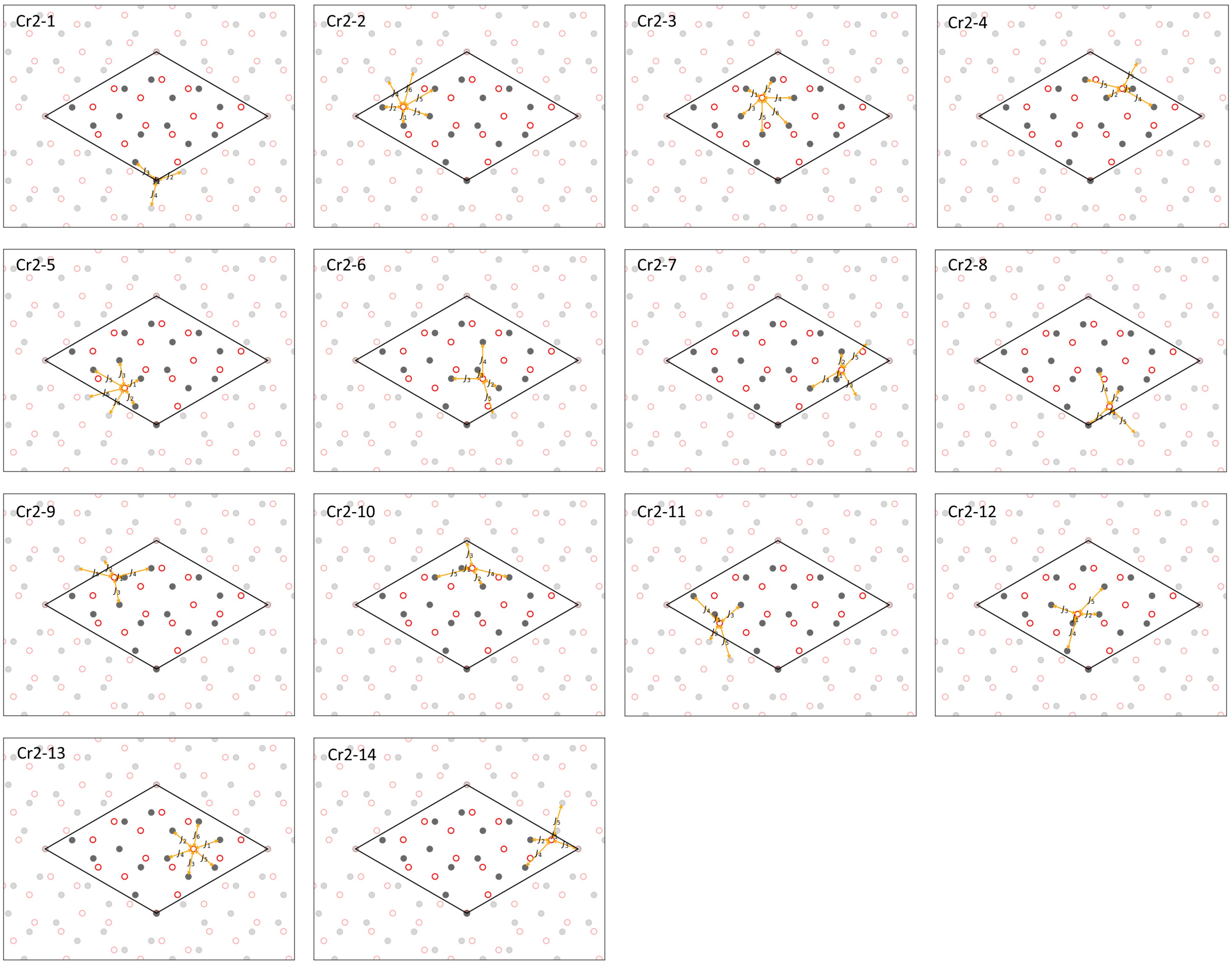

Figure S8. Atom-resolved interlayer Heisenberg exchange couplings for the bottom layer in twisted bilayer CrI_3.

Table S3 The DFT-resolved magnitudes (meV) of intralayer exchange couplings in twisted bilayer CrI_3.

Cr	J_1	J_2	J_3	J_4	J_5	J_6	J_7	J_8	J_9
1-1	1.2307	1.2690	1.1731	0.2525	0.2524	0.2547	0.2343	0.2519	0.2541
1-2	1.1729	1.1580	1.1443	0.2548	0.2550	0.2343	0.2519	0.2500	0.2500
1-3	1.1736	1.1575	1.1441	0.2548	0.2550	0.2344	0.2519	0.2500	0.2501
1-4	1.2101	1.2105	1.2106	0.2550	0.2551	0.2550	0.2540	0.2541	0.2541
1-5	1.2302	1.2695	1.1731	0.2524	0.2525	0.2548	0.2344	0.2519	0.2540
1-6	1.2307	1.2695	1.1725	0.2524	0.2524	0.2548	0.2344	0.2518	0.2541
1-7	1.1735	1.1582	1.1438	0.2547	0.2551	0.2344	0.2518	0.2501	0.2500
1-8	1.1580	1.2690	1.2106	0.2540	0.2569	0.2534	0.2548	0.2309	0.2308
1-9	1.1729	1.1441	1.1731	0.2565	0.2564	0.2570	0.2533	0.2548	0.2446
1-10	1.1575	1.2695	1.2105	0.2539	0.2570	0.2533	0.2549	0.2308	0.2308
1-11	1.1582	1.2695	1.2101	0.2538	0.2570	0.2534	0.2548	0.2308	0.2309
1-12	1.2302	1.2307	1.2307	0.2538	0.2540	0.2539	0.2445	0.2446	0.2446
1-13	1.1735	1.1443	1.1725	0.2563	0.2565	0.2570	0.2534	0.2548	0.2445
1-14	1.1736	1.1438	1.1731	0.2563	0.2564	0.2569	0.2534	0.2549	0.2446
2-1	1.2101	1.2103	1.2107	0.2550	0.2551	0.2550	0.2541	0.2541	0.2540
2-2	1.1737	1.1576	1.1440	0.2547	0.2550	0.2344	0.2518	0.2500	0.2501
2-3	1.1730	1.1579	1.1443	0.2548	0.2550	0.2344	0.2519	0.2500	0.2500
2-4	1.2309	1.2690	1.1729	0.2525	0.2524	0.2547	0.2344	0.2518	0.2541
2-5	1.1733	1.1582	1.1438	0.2547	0.2551	0.2343	0.2518	0.2501	0.2500
2-6	1.2305	1.2696	1.1726	0.2524	0.2524	0.2548	0.2344	0.2518	0.2540
2-7	1.2302	1.2693	1.1733	0.2524	0.2525	0.2547	0.2343	0.2519	0.2541
2-8	1.1576	1.2696	1.2103	0.2539	0.2570	0.2533	0.2549	0.2308	0.2308
2-9	1.1730	1.1440	1.1733	0.2565	0.2563	0.2569	0.2533	0.2548	0.2447
2-10	1.1579	1.2690	1.2107	0.2540	0.2570	0.2533	0.2548	0.2309	0.2308
2-11	1.1737	1.1438	1.1729	0.2564	0.2563	0.2570	0.2534	0.2549	0.2445
2-12	1.1733	1.1443	1.1726	0.2564	0.2565	0.2570	0.2533	0.2548	0.2446
2-13	1.2302	1.2305	1.2309	0.2539	0.2540	0.2539	0.2446	0.2447	0.2445
2-14	1.1582	1.2693	1.2101	0.2539	0.2569	0.2534	0.2548	0.2308	0.2309

Table S4 The DFT-resolved magnitudes (meV) of interlayer exchange couplings in twisted bilayer CrI_3.

Cr	J_1^c	J_2^c	J_3^c	J_4^c	J_5^c	J_6^c
1-1	0.0082	−0.1044	−0.0109	0.0025	0.0203	
1-2	−0.1044	−0.0897	−0.0260	0.1087	0.0025	0.0613
1-3	−0.1045	−0.0897	−0.0261	0.1086	0.0025	0.0613
1-4	0.0595	0.0127	0.0127	0.0127		
1-5	0.0081	−0.1045	−0.0109	0.0025	0.0204	
1-6	0.0082	−0.1044	−0.0110	0.0025	0.0204	
1-7	−0.1045	−0.0897	−0.0261	0.1086	0.0025	0.0613
1-8	−0.1249	−0.0897	0.0127	−0.0164	0.0203	
1-9	0.0082	0.1370	−0.0752	−0.0164	0.0613	
1-10	−0.1249	−0.0897	0.0127	−0.0164	0.0204	
1-11	−0.1249	−0.0897	0.0127	−0.0164	0.0204	
1-12	−0.0261	−0.0260	−0.0261	−0.0752	−0.0752	−0.0752
1-13	0.0081	0.1371	−0.0752	−0.0164	0.0613	
1-14	0.0081	0.1370	−0.0752	−0.0164	0.0613	
2-1	0.0595	0.0127	0.0127	0.0127		
2-2	−0.1045	−0.0897	−0.0261	0.1086	0.0025	0.0613
2-3	−0.1044	−0.0897	−0.0260	0.1087	0.0025	0.0613
2-4	0.0082	−0.1044	−0.0109	0.0025	0.0203	
2-5	−0.1044	−0.0897	−0.0261	0.1086	0.0025	0.0613
2-6	0.0081	−0.1045	−0.0110	0.0025	0.0204	
2-7	0.0081	−0.1045	−0.0109	0.0025	0.0204	
2-8	−0.1249	−0.0897	0.0127	−0.0164	0.0204	
2-9	0.0082	0.1370	−0.0752	−0.0164	0.0613	
2-10	−0.1249	−0.0897	0.0127	−0.0164	0.0203	
2-11	0.0081	0.1370	−0.0752	−0.0164	0.0613	
2-12	0.0082	0.1371	−0.0752	−0.0164	0.0613	
2-13	−0.0261	−0.0260	−0.0261	−0.0752	−0.0752	−0.0752
2-14	−0.1249	−0.0897	0.0127	−0.0164	0.0204	

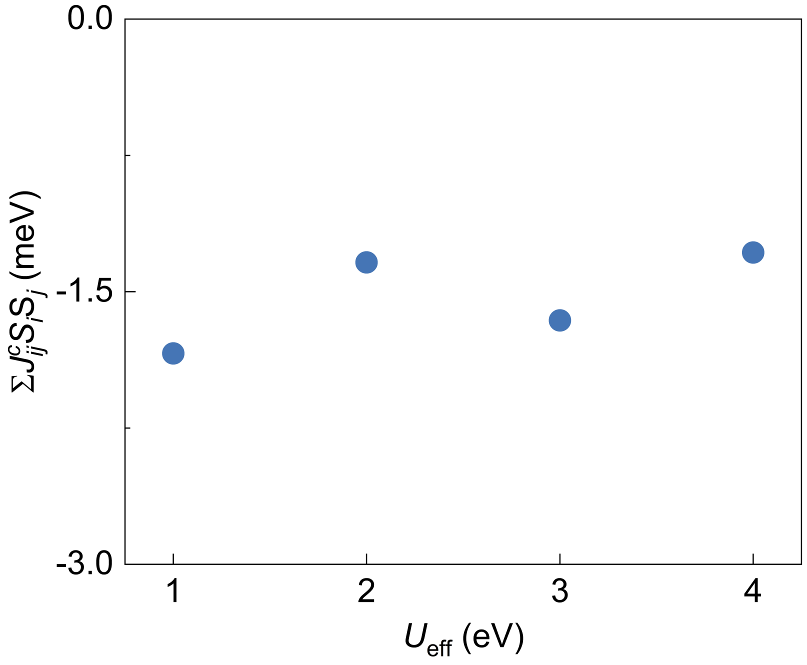

Figure S9. The sum of interlayer coupling contributions, represented by $\sum J^{c}_{ij}S_iS_j$, to the interlayer antiferromagnetic coupling as a function of the effective Hubbard parameter U_{eff}

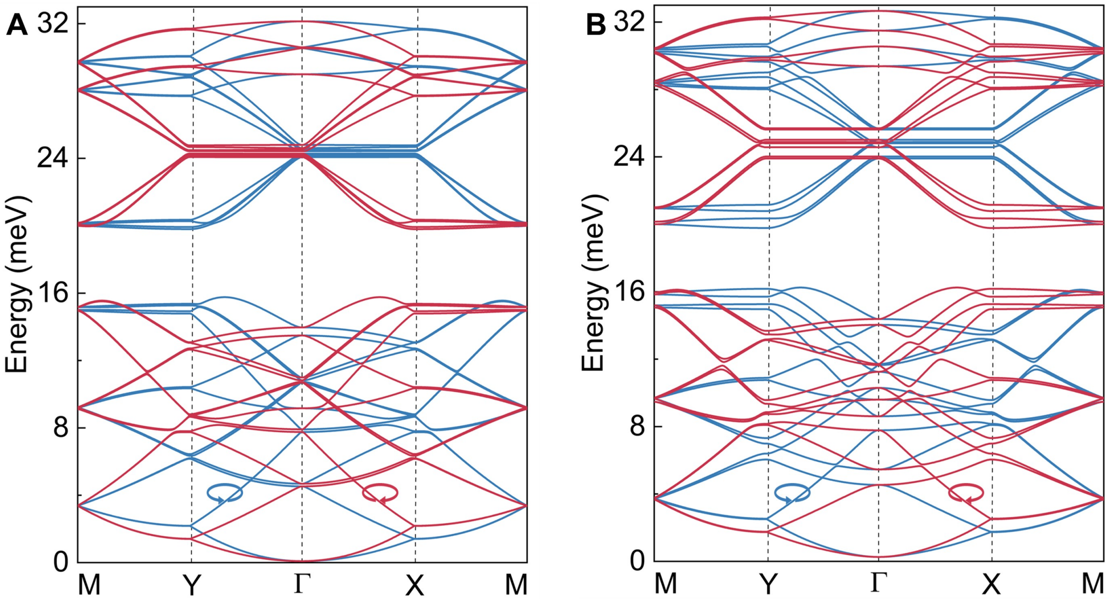

Figure S10. The spin-split magnon spectrum of α (right-handed) and β (left-handed) modes, with scaling factor of interlayer couplings **A** $g = 0.1$ and **B** $g = 10$. By setting g=0.1, the interlayer couplings energy contribution is reduced to approximately from -1.5 to -0.15 meV, becoming orders of magnitude smaller than that from intralayer couplings. Despite this drastic reduction, clear magnon spin splitting persists. Furthermore, we increase g to 10, strongly enhancing interlayer interactions; again, significant spin splitting is clearly observed, although the overall magnon dispersion is correspondingly scaled up in energy.

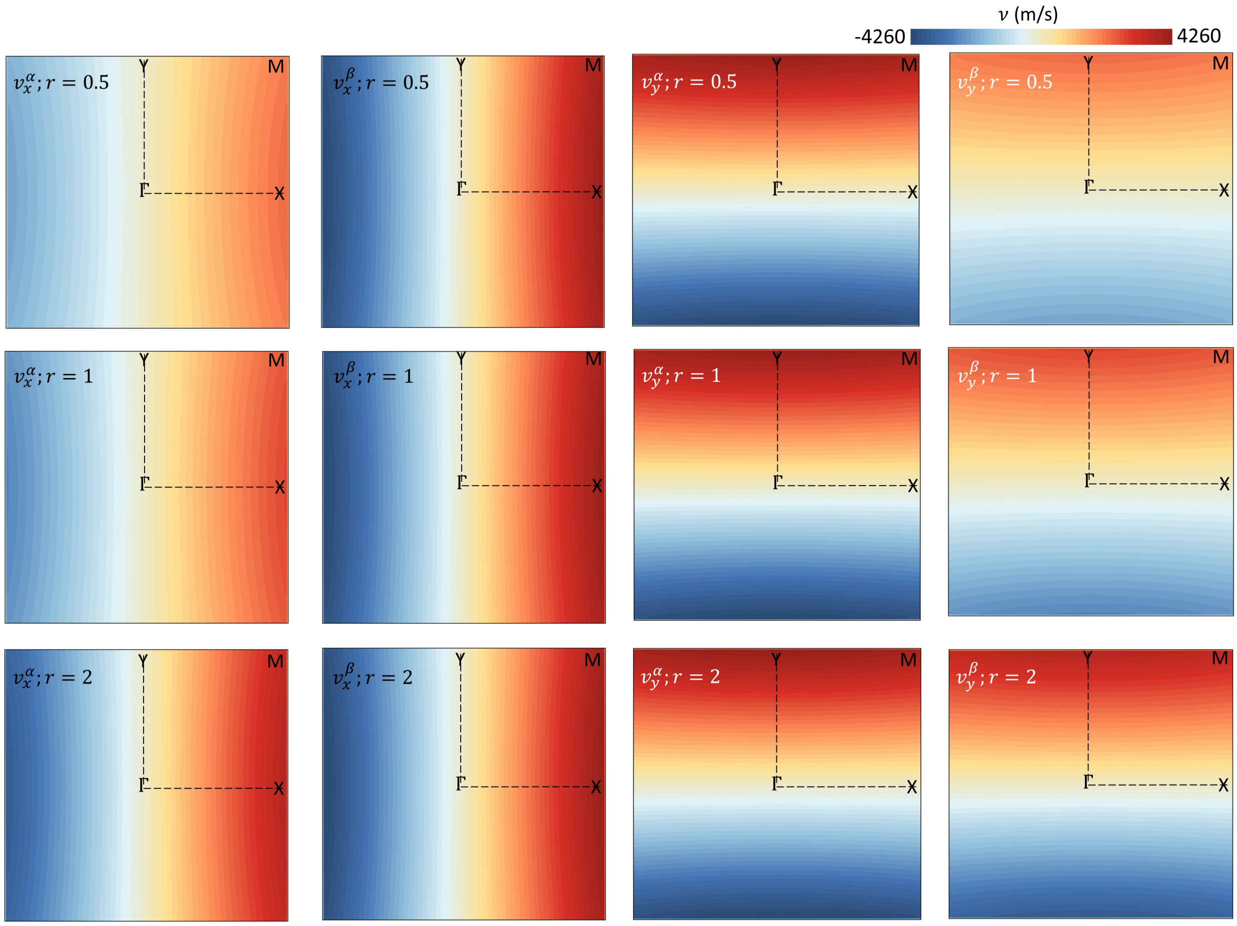

Figure S11. The group velocity v of the ground acoustic magnon mode in twisted bilayer $CrPS_4$. For the α and β modes, the velocity is defined as $v_n^\alpha = (1/\hbar)(T_{\mathbf{k}}^\dagger(\partial_{k_n}\hat{H}_{\mathbf{k}})T_{\mathbf{k}})_{\alpha\alpha}$ and $v_n^\beta = (1/\hbar)(T_{\mathbf{k}}^\dagger(\partial_{k_n}\hat{H}_{\mathbf{k}})T_{\mathbf{k}})_{\beta\beta}$. One can see that v exhibits a significant anisotropy along main crystal axis. The factor r, when multiplied by the J_3 and J_4 of each Cr atom, directly modulates the anisotropic ratio of altermagnetic magnons.

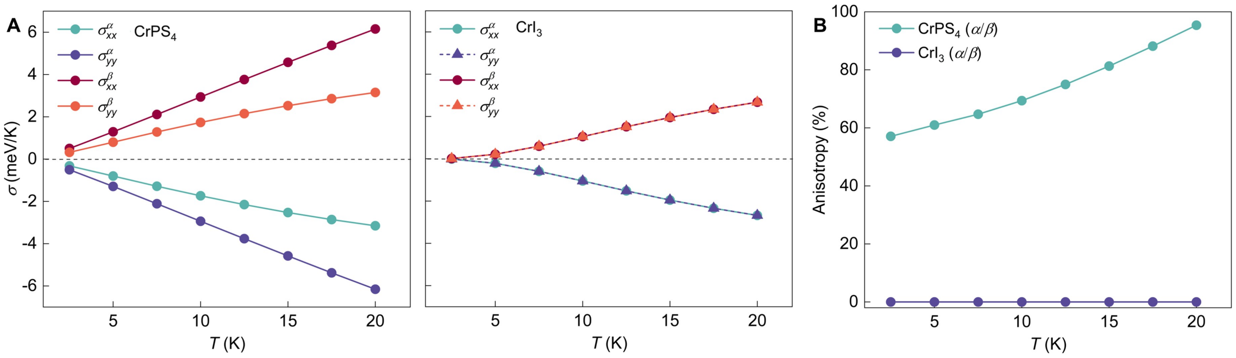

Figure S12. **A** The spin conductivities of the α and β modes under thermal gradients along the main axes are presented as functions of temperature. The left panel corresponds to the twisted bilayer $CrPS_4$, and the right panel corresponds to the twisted bilayer CrI_3. It is clearly observed that very lager anisotropic magnon transport emerges in $CrPS_4$, whereas the magnon transport remains to be degenerate along different main axes in CrI_3. **B** The anisotropic ratio of magnon transport along different main axes is defined as $(|\sigma_{yy}^{\alpha(\beta)}| - |\sigma_{xx}^{\alpha(\beta)}|)/|\sigma_{xx}^{\alpha(\beta)}|$.

IV. *Magnonic Transports*—Within the linear response regime, the spin current density induced by a temperature gradient ∇T is given by:

$$\begin{pmatrix} j_x^z \\ j_y^z \end{pmatrix} = \begin{pmatrix} \sigma_{xx} & 0 \\ 0 & \sigma_{yy} \end{pmatrix} \begin{pmatrix} (-\partial_n T)\cos\varphi \\ (-\partial_n T)\sin\varphi \end{pmatrix} \tag{13}$$

Based on the Kubo theory [15], the spin current conductivity $\sigma_{mn} = \sigma_{mn}^{\alpha} + \sigma_{mn}^{\beta}$ with $\sigma_{mn}^{\eta} = \frac{\gamma^{\eta}\tau_0}{AT}\sum_i \sum_{\mathbf{k}} (v_m^i)_{\eta\eta} (v_n^i)_{\eta\eta} \omega_{\mathbf{k},\eta}^i (-\partial_\varepsilon \rho)$, where $\eta = \alpha, \beta$ distinguishes the two modes, and $\gamma^{\alpha(\beta)} = 1(-1)$ accounts for their opposite spin angular momentum. τ_0, A, T, i and ω represents magnon lifetime, sample area, temperature, band index and eigenfrequency, respectively. The effective group velocity is $v_m^i = T_{\mathbf{k}}^{\dagger} (\partial_{k_m} H_{\mathbf{k}}) T_{\mathbf{k}}$. $\tau_0 = 0.05$ ns is chosen following state-of-the-art time-resolved reflectivity measurements of magnons in few-layer vdW antiferromagnets [16]. The combination of magnonic currents from the α and β modes ultimately determines the longitudinal and transverse spin currents. Specifically, we have $\sigma_{\parallel} = \sigma_{\parallel}^{\alpha} + \sigma_{\parallel}^{\beta}$ and $\sigma_{\perp} = \sigma_{\perp}^{\beta} - \sigma_{\perp}^{\alpha}$, where $\sigma_{\parallel}^{\alpha} = \sigma_{xx}^{\alpha}\cos^2\varphi + \sigma_{yy}^{\alpha}\sin^2\varphi, \sigma_{\parallel}^{\beta} = \sigma_{xx}^{\beta}\cos^2\varphi + \sigma_{yy}^{\beta}\sin^2\varphi, \sigma_{\perp}^{\alpha} = \left(\sigma_{yy}^{\alpha} - \sigma_{xx}^{\alpha}\right)\cos\varphi\sin\varphi, \sigma_{\perp}^{\beta} = \left(\sigma_{xx}^{\beta} - \sigma_{yy}^{\beta}\right)\cos\varphi\sin\varphi$.

V. *Twist–tunable spin transport*—The twist angle between adjacent layers in 2D materials introduces a novel degree of freedom, giving rise to remarkable physical phenomena that define the emerging field of twistronics. Advanced layer-transfer techniques now enable precise fabrication of multilayers with arbitrary interlayer twist angles [17, 18]. For $CrPS_4$ single layer, we find the $\sigma^{\uparrow}_{aa}$=-$\sigma^{\downarrow}_{aa}$= 4.77 and $\sigma^{\uparrow}_{bb}$=-$\sigma^{\downarrow}_{bb}$= 2.63 meV/K. The magnon-mediated spin transport can be written as:

$$\sigma^{\downarrow}_{\parallel} = \sigma^{\downarrow}_{bb}\cos^2\varphi + \sigma^{\downarrow}_{aa}\sin^2\varphi; \sigma^{\downarrow}_{\perp} = (\sigma^{\downarrow}_{aa} - \sigma^{\downarrow}_{bb})\sin\varphi\cos\varphi,$$
$$\sigma^{\uparrow}_{\parallel} = \sigma^{\uparrow}_{bb}\cos^2(\theta-\varphi) + \sigma^{\uparrow}_{aa}\sin^2(\theta-\varphi); \sigma^{\uparrow}_{\perp} = (\sigma^{\uparrow}_{aa} - \sigma^{\uparrow}_{bb})\sin(\theta-\varphi)\cos(\theta-\varphi),$$
$$\sigma_{\parallel} = \sigma^{\uparrow}_{\parallel} + \sigma^{\downarrow}_{\parallel}; \sigma_{\perp} = \sigma^{\uparrow}_{\perp} - \sigma^{\downarrow}_{\perp}$$

Since the top and bottom layer are identical materials, we have the relationship: $\sigma^{\downarrow}_{bb} = -\sigma^{\uparrow}_{bb}$ and $\sigma^{\downarrow}_{aa} = -\sigma^{\uparrow}_{aa}$. Consequently, we have $\sigma_{\parallel} = \sigma^{\uparrow}_{bb}[\cos^2(\theta-\varphi) - \cos^2\varphi] + \sigma^{\uparrow}_{aa}[\sin^2(\theta-\varphi) - \sin^2\varphi] = (\sigma^{\uparrow}_{aa} - \sigma^{\uparrow}_{bb})\sin\theta\sin(\theta-2\varphi)$ and $\sigma_{\perp} = (\sigma^{\uparrow}_{aa} - \sigma^{\uparrow}_{bb})[\sin(\theta-\varphi)\cos(\theta-\varphi) + \sin\varphi\cos\varphi] = \frac{\sigma^{\uparrow}_{aa} - \sigma^{\uparrow}_{bb}}{2}[\sin(2\theta-2\varphi) + \sin(2\varphi)]$. By solving the equations $\partial\sigma_{\parallel(\perp)}/\partial\theta = 0$ and $\partial\sigma_{\parallel(\perp)}/\partial\varphi = 0$, we can obtain the optimal angle configurations that the largest $\sigma_{\parallel}$ is achieved at (θ=90°, φ= 0°/90°/180°...), while the largest $\sigma_{\perp}$ is realized at (θ=90°, φ= 45°/135°/225°...). At these optimal angle configurations, the maximum magnitude of spin conductivity reaches up to 2.14 meV/K, fairly consistent with 2.05 meV/K derived from the explicit spin Hamiltonian of twisted antiferromagnetic bilayer. This small magnitude discrepancy arises due to the neglect of interlayer couplings in calculations of θ-dependent magnonic transport.

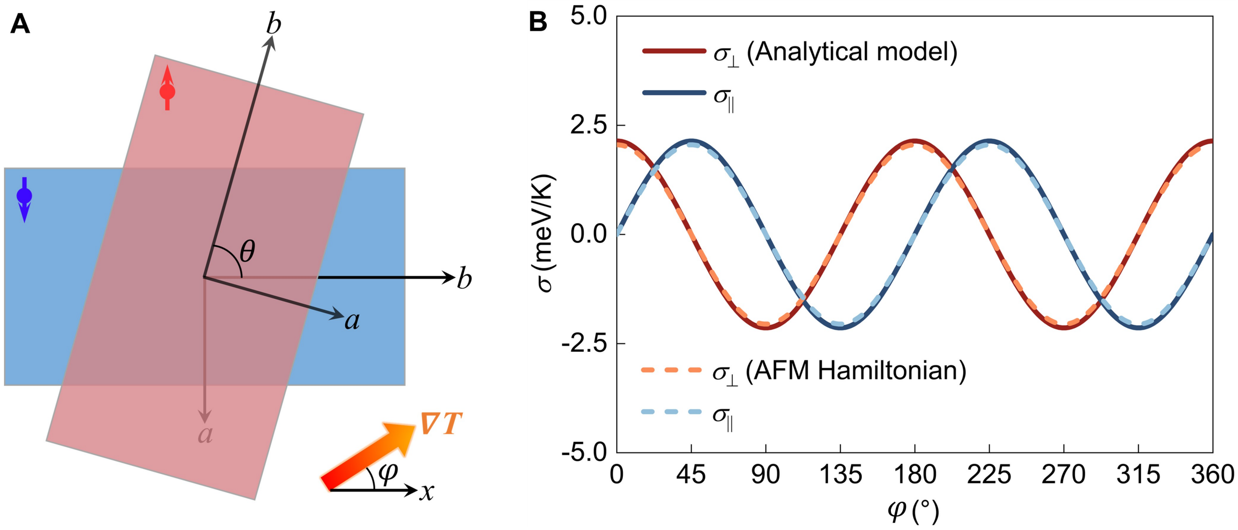

Figure S13. **A** Schematic of a twisted vdW antiferromagnet. The ferromagnetic order is retained within each layer, while adjacent layers are coupled antiferromagnetically. The interlayer twist angle is denoted by θ, and φ indicates the orientation of the applied thermal gradient. **B** Comparison of spin conductivity derived from the analytical model without interlayer exchange and the antiferromagnetic spin Hamiltonian incorporating interlayer exchange.

VI. *Comparison with nontwisted 2D altermagnets*—Figure S14 shows the crystal structures of 2D altermagnets Fe_2Se_2O and Mn_2PTe [19, 20]. The $[\mathcal{C}_2\|\mathcal{P}]$ symmetry breaking requires the nearest-neighboring spin sites in each sublattice being connected anisotropically in a and b directions. Consequently, the bridging electronic states give rise to anisotropic superexchange coupling, i.e., $J_2 \neq J_2'$. Meanwhile, $[\mathcal{C}_2\|\mathcal{M}_\phi]$ symmetry requires spin-connected types of sublattices "a" and "b" being exactly swapped in a and b, which results in the switched anisotropic exchange for two sublattices. The symmetry-determined spin Hamiltonian reads: $H = -J_1 \sum_{<i,j>} \boldsymbol{S}_{ai} \cdot \boldsymbol{S}_{bj} - J_2 \sum_{<i_x,j_x>} \boldsymbol{S}_{ai} \cdot \boldsymbol{S}_{aj} - J_2' \sum_{<i_y,j_y>} \boldsymbol{S}_{ai} \cdot \boldsymbol{S}_{aj} - K \sum_i (S_{ai}^r)^2 - J_2' \sum_{<i_x,j_x>} \boldsymbol{S}_{bi} \cdot \boldsymbol{S}_{bj} - J_2 \sum_{<i_y,j_y>} \boldsymbol{S}_{bi} \cdot \boldsymbol{S}_{bj} - K \sum_i (S_{bi}^r)^2$. Using the Holstein-Primakoff transformation, the spin Hamiltonian in the basis of magnon operators in momentum space $\psi_{\mathbf{k}} = \left(a_{\mathbf{k}}, b_{\mathbf{k}}^\dagger\right)^T$ is written as: $H = \sum_{\mathbf{k}} \psi_{\mathbf{k}}^\dagger \hat{H}_{\mathbf{k}} \psi_{\mathbf{k}}$ with

$$\hat{H}_{\mathbf{k}} = S \begin{pmatrix} J^* - 2J_2\gamma_1 - 2J_2'\gamma_2 & -4J_1\gamma_3 \\ -4J_1\gamma_3 & J^* - 2J_2'\gamma_1 - 2J_2\gamma_2 \end{pmatrix}, \tag{14}$$

where on-site energy $J^* = -4J_1 + 2J_2 + 2J_2' + 2K$, and structure factors $\gamma_1 = \cos(k_x a)$, $\gamma_2 = \cos(k_y a)$, $\gamma_3 = \cos(k_x a/2)\cos(k_y a/2)$. For Fe_2Se_2O, $J_1 = -13.9$, $J_2' = -1.9$, $J_2 = -3.8$, $K = 0.28$ meV, and for Mn_2PTe, $J_1 = -5.9$, $J_2' = 3.7$, $J_2 = -1.1$, $K = 0.39$ meV. The spin Hamiltonian can be diagonalized by the Bogoliubov transformation $\psi_{\mathbf{k}} = T_{\mathbf{k}}\Psi_{\mathbf{k}}$. The Hamiltonian containing α and β modes in the basis of magnon operators $\Psi_{\mathbf{k}} = (\alpha_{\mathbf{k}}, \beta_{\mathbf{k}}^\dagger)^T$ is rewritten as:

$$H_{\mathbf{k}} = \Psi_{\mathbf{k}}^\dagger \begin{pmatrix} E_{\mathbf{k}}^\alpha & 0 \\ 0 & E_{\mathbf{k}}^\beta \end{pmatrix} \Psi_{\mathbf{k}}, \tag{15}$$

with eigenvalues: $E_{\mathbf{k}}^\alpha = S\left[J_2' - J_2\right)(\gamma_1 - \gamma_2) + \rho_{\mathbf{k}}]$; $E_{\mathbf{k}}^\beta = S\left[J_2 - J_2'\right)(\gamma_1 - \gamma_2) + \rho_{\mathbf{k}}\Big]$, where $\rho_{\mathbf{k}} = \sqrt{\left[4J_1 - 2K - (J_2 + J_2')(2 - \gamma_1 - \gamma_2)\right]^2 - (4J_1\gamma_3)^2}$. Following the Kubo theory (see section IV), the magnon-mediated spin conductivity is then resolved. We obtain $\sigma_{xx}^\beta = -\sigma_{yy}^\alpha = 1.47 \times 10^{-3}$, $\sigma_{yy}^\beta = -\sigma_{xx}^\alpha = 1.35 \times 10^{-3}$ (Fe_2Se_2O), and $\sigma_{xx}^\beta = -\sigma_{yy}^\alpha = 1.35 \times 10^{-3}$, $\sigma_{yy}^\beta = -\sigma_{xx}^\alpha = 0.90 \times 10^{-3}$ meV/K (Mn_2PTe), at $T = 15$ K. The corresponding maximum magnitude of $\sigma_\parallel(\sigma_\perp)$ reaches to 0.12×10^{-3} and 0.45×10^{-3} meV/K for these two candidates, respectively (Figure S15A). These values are several orders of magnitude smaller than the spin conductivity observed in twisted bilayer $CrPS_4$. Fe_2Se_2O and Mn_2PTe have large magnetic anisotropy and inter-sublattice couplings, directly increasing the magnonic excitation energy (see eigenvalue expressions where the magnonic energy at Γ point reads:

$S\sqrt{4K^2 - 16J_1K}$). The resulting magnon occupation is therefore substantially lower than that in twisted $CrPS_4$ at finite temperatures, leading to a greatly reduced spin conductivity.

Since magnetic anisotropy can vary among different twisted or non-twisted altermagnets, we further artificially set the K to zero to exclude its effects. We obtain $\sigma^{\beta}_{xx} = -\sigma^{\alpha}_{yy} = 2.17$, $\sigma^{\beta}_{yy} = -\sigma^{\alpha}_{xx} = 2.13$ (Fe_2Se_2O), and $\sigma^{\beta}_{xx} = -\sigma^{\alpha}_{yy} = 2.25$, $\sigma^{\beta}_{yy} = -\sigma^{\alpha}_{xx} = 2.06$ meV/K (Mn_2PTe). The spin conductivity of each mode is significantly increased. Despite the maximum of $\sigma_{\parallel}(\sigma_{\perp})$ increases to 0.04 and 0.19 meV/K (Figure S15B), which is still at least one order smaller compared to twisted $CrPS_4$. The eigenvalue expressions indicate that the distinction between the α and β modes originate from differences in the intra-sublattice exchange couplings along different main axes, whereas the inter-sublattice couplings contribute equally to both modes. This explains why the twisted bilayer exhibits significantly enhanced spin Seebeck and spin Nernst effects. Specifically, (i) the inherently weaker interlayer coupling vdW antiferromagnets compared to intralayer interactions, and (ii) the reduced in-plane symmetry imparting anisotropy along different main axes to magnons in a single layer, results in that α and β modes exhibits a large anisotropy at low energies. Although altermagnetic magnons have been experimentally observed in $g-$wave MnTe and CrSb, the coexistence of $[E\|\mathcal{C}_{3\perp}]$ and $[\mathcal{C}_2\|\mathcal{C}_{6\perp}t]$ symmetries ensures that the thermal spin currents generated by the α and β modes cancel each other, resulting in no observable net spin signal.

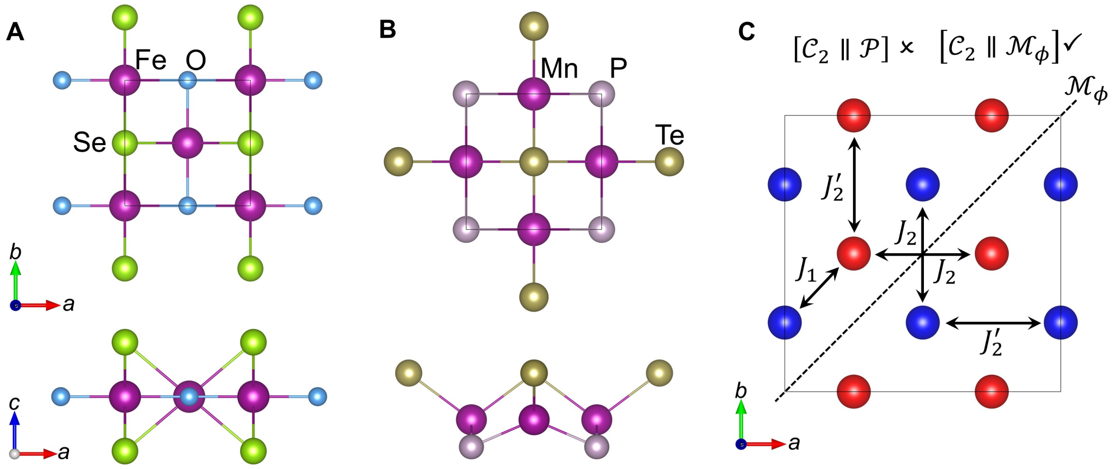

Figure S14. The top (upper panel) and side (down panel) views of **A** Fe_2Se_2O and **B** Mn_2PTe monolayers. **C** The corresponding spin lattices in these two altermagnets. The broken $[\mathcal{C}_2\|\mathcal{P}]$ symmetry and preserved $[\mathcal{C}_2\|\mathcal{M}_\phi]$ symmetry leads to the alternating distribution of anisotropic intralayer exchange couplings in two opposite-spin sublattices.

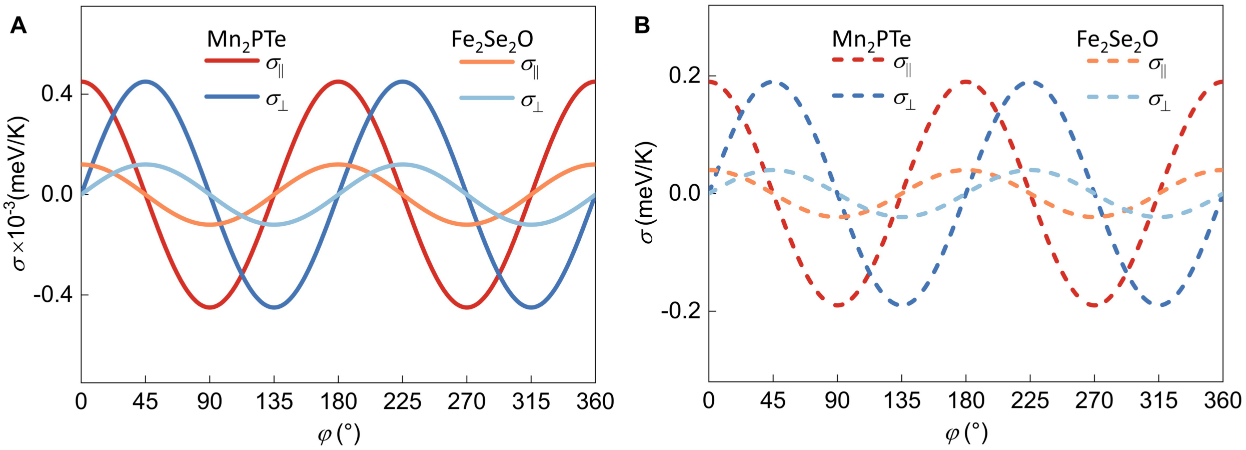

Figure S15. The thermal gradient orientation-dependent longitudinal(transverse) spin conductivity $\sigma_\parallel(\sigma_\perp)$ at $T = 15$ K, in Fe_2Se_2O and MnPTe monolayers. **A** and **B** denote the spin conductivity calculated with and without magnetic anisotropy, respectively.

VII. *Band dispersions around* Γ *point*—We now investigate magnon eigenvalues with the limitation $\mathbf{k} \to 0$, based on the general altermagnetic model (see section VI). The Taylor expansion is then utilized for estimation purposes. The first part of eigenvalue $(J_2' - J_2)(\gamma_1 - \gamma_2)$ can be approximated as $(J_2' - J_2)(k_y^2 - k_x^2)a^2/2$, and the second part $\rho_\mathbf{k} \approx \sqrt{[2J_1^2 - (4J_1 - 2K)(J_2 + J_2')]k^2a^2 + 4K^2 - 16J_1K}$ is approximated as $\sqrt{4K^2 - 16J_1K} + \frac{J_1^2-(2J_1-K)(J_2+J_2')}{\sqrt{4K^2-16J_1K}}k^2a^2$. If the magnetic anisotropy K is neglected, the leading order term in the Taylor expansion of $\rho_\mathbf{k}$ is $\sqrt{2J_1^2 - 4J_1(J_2 + J_2')}|\mathbf{k}|a$.

The first contribution determined solely by intra-sublattice couplings exhibits a quadratic dependence on $\mathbf{k}$, and the second contribution, including the inter-sublattice couplings, is also quadratic in $\mathbf{k}$. Interestingly, the second contribution exhibit a linear dependence on $\mathbf{k}$ once the K is neglected. The above analysis indicates that for altermagnets, (i) magnon dispersions exhibit quadratic behavior near the Γ point when the atomic spin Hamiltonian includes a nonzero K, and (ii) linear dispersions emerge upon neglecting K. This is consistent with known theory of antiferromagnetic magnons [21].

These theoretical prediction matches very well the explicit magnon dispersions obtained for twisted bilayer $CrPS_4$. Figure S16**A** and **B** show the magnon dispersions near the Γ point with and without K. This effect may have been overlooked in some earlier reports of altermagnetic magnons, as K was not considered in the spin Hamiltonian [22], because they focus on non-relativistic effects.

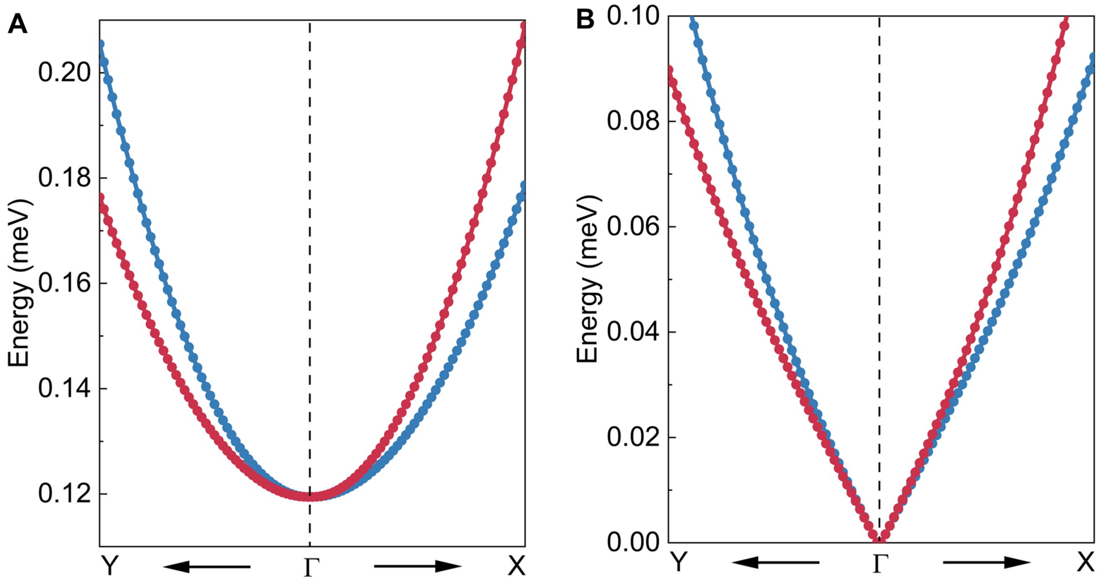

Figure S16. **A** and **B** Altermagnetic magnon dispersions around the Γ point $(0.1 \times \frac{2\pi}{a})$ in twisted bilayer $CrPS_4$ with and without considering K.

$$
\begin{aligned}
(A_{\mathbf{k}})_{1,1} &= J^1_{1,2} + J^1_{1,4} + J^1_{1,5} + J^1_{1,6} + J^1_{1,8} + J^1_{1,21} + J^1_{1,22} + J^1_{1,24} \\
&- J^{12}_{1,1} - J^{12}_{1,5} - J^{12}_{1,9} - J^{12}_{1,2} - J^{12}_{1,6} - J^{12}_{1,10} + 2K \\
(A_{\mathbf{k}})_{1,2} &= \Big[(A_{\mathbf{k}})_{2,1}\Big]^* = -J^1_{1,2},\ (A_{\mathbf{k}})_{1,4} = \Big[(A_{\mathbf{k}})_{4,1}\Big]^* = -J^1_{1,4}e^{i\mathbf{k}\cdot\delta_2},\ (A_{\mathbf{k}})_{1,5} = \Big[(A_{\mathbf{k}})_{5,1}\Big]^* = -J^1_{1,5} \\
(A_{\mathbf{k}})_{1,6} &= \Big[(A_{\mathbf{k}})_{6,1}\Big]^* = -J^1_{1,6},\ (A_{\mathbf{k}})_{1,8} = \Big[(A_{\mathbf{k}})_{8,1}\Big]^* = -J^1_{1,8}e^{i\mathbf{k}\cdot\delta_2}, (A_{\mathbf{k}})_{1,21} = \Big[(A_{\mathbf{k}})_{21,1}\Big]^* = -J^1_{1,21}e^{i\mathbf{k}\cdot\delta_4} \\
(A_{\mathbf{k}})_{1,22} &= \Big[(A_{\mathbf{k}})_{22,1}\Big]^* = -J^1_{1,22}e^{i\mathbf{k}\cdot\delta_4},\ (A_{\mathbf{k}})_{1,24} = \Big[(A_{\mathbf{k}})_{24,1}\Big]^* = -J^1_{1,24}e^{i\mathbf{k}\cdot\delta_6} \\
(A_{\mathbf{k}})_{2,2} &= J^1_{2,1} + J^1_{2,3} + J^1_{2,5} + J^1_{2,6} + J^1_{2,7} + J^1_{2,21} + J^1_{2,22} + J^1_{2,23} \\
&- J^{12}_{2,9} - J^{12}_{2,10} - J^{12}_{2,13} - J^{12}_{2,14} - J^{12}_{2,5} - J^{12}_{2,6} - J^{12}_{2,17} - J^{12}_{2,18} + 2K \\
(A_{\mathbf{k}})_{2,3} &= \Big[(A_{\mathbf{k}})_{3,2}\Big]^* = -J^1_{2,3},\ (A_{\mathbf{k}})_{2,5} = \Big[(A_{\mathbf{k}})_{5,2}\Big]^* = -J^1_{2,5},\ (A_{\mathbf{k}})_{2,6} = \Big[(A_{\mathbf{k}})_{6,2}\Big]^* = -J^1_{2,6} \\
(A_{\mathbf{k}})_{2,7} &= \Big[(A_{\mathbf{k}})_{7,2}\Big]^* = -J^1_{2,7},\ (A_{\mathbf{k}})_{2,21} = \Big[(A_{\mathbf{k}})_{21,2}\Big]^* = -J^1_{2,21}e^{i\mathbf{k}\cdot\delta_4} \\
(A_{\mathbf{k}})_{2,22} &= \Big[(A_{\mathbf{k}})_{22,2}\Big]^* = -J^1_{2,22}e^{i\mathbf{k}\cdot\delta_4},\ (A_{\mathbf{k}})_{2,23} = \Big[(A_{\mathbf{k}})_{23,2}\Big]^* = -J^1_{2,23}e^{i\mathbf{k}\cdot\delta_4} \\
(A_{\mathbf{k}})_{3,3} &= J^1_{3,2} + J^1_{3,4} + J^1_{3,6} + J^1_{3,7} + J^1_{3,8} + J^1_{3,22} + J^1_{3,23} + J^1_{3,24} \\
&- J^{12}_{3,13} - J^{12}_{3,14} - J^{12}_{3,17} - J^{12}_{3,18} - J^{12}_{3,21} - J^{12}_{3,22} + 2K \\
(A_{\mathbf{k}})_{3,4} &= \Big[(A_{\mathbf{k}})_{4,3}\Big]^* = -J^1_{3,4},\ (A_{\mathbf{k}})_{3,6} = \Big[(A_{\mathbf{k}})_{6,3}\Big]^* = -J^1_{3,6},\ (A_{\mathbf{k}})_{3,7} = \Big[(A_{\mathbf{k}})_{7,3}\Big]^* = -J^1_{3,7} \\
(A_{\mathbf{k}})_{3,8} &= \Big[(A_{\mathbf{k}})_{8,3}\Big]^* = -J^1_{3,8},\ (A_{\mathbf{k}})_{3,22} = \Big[(A_{\mathbf{k}})_{22,3}\Big]^* = -J^1_{3,22}e^{i\mathbf{k}\cdot\delta_4} \\
(A_{\mathbf{k}})_{3,23} &= \Big[(A_{\mathbf{k}})_{23,3}\Big]^* = -J^1_{3,23}e^{i\mathbf{k}\cdot\delta_4},\ (A_{\mathbf{k}})_{3,24} = \Big[(A_{\mathbf{k}})_{24,3}\Big]^* = -J^1_{3,24}e^{i\mathbf{k}\cdot\delta_4} \\
(A_{\mathbf{k}})_{4,4} &= J^1_{4,1} + J^1_{4,3} + J^1_{4,5} + J^1_{4,7} + J^1_{4,8} + J^1_{4,21} + J^1_{4,23} + J^1_{4,24} \\
&- J^{12}_{4,21} - J^{12}_{4,22} - J^{12}_{4,17} - J^{12}_{4,18} - J^{12}_{4,1} - J^{12}_{4,2} - J^{12}_{4,5} - J^{12}_{4,6} + 2K \\
(A_{\mathbf{k}})_{4,5} &= \Big[(A_{\mathbf{k}})_{5,4}\Big]^* = -J^1_{4,5}e^{i\mathbf{k}\cdot\delta_1},\ (A_{\mathbf{k}})_{4,7} = \Big[(A_{\mathbf{k}})_{7,4}\Big]^* = -J^1_{4,7} \\
(A_{\mathbf{k}})_{4,8} &= \Big[(A_{\mathbf{k}})_{8,4}\Big]^* = -J^1_{4,8},\ (A_{\mathbf{k}})_{4,21} = \Big[(A_{\mathbf{k}})_{21,4}\Big]^* = -J^1_{4,21}e^{i\mathbf{k}\cdot\delta_7} \\
(A_{\mathbf{k}})_{4,23} &= \Big[(A_{\mathbf{k}})_{23,4}\Big]^* = -J^1_{4,23}e^{i\mathbf{k}\cdot\delta_4},\ (A_{\mathbf{k}})_{4,24} = \Big[(A_{\mathbf{k}})_{24,4}\Big]^* = -J^1_{4,24}e^{i\mathbf{k}\cdot\delta_4} \\
(A_{\mathbf{k}})_{5,5} &= J^1_{5,1} + J^1_{5,2} + J^1_{5,4} + J^1_{5,6} + J^1_{5,8} + J^1_{5,9} + J^1_{5,10} + J^1_{5,12} \\
&- J^{12}_{5,1} - J^{12}_{5,2} - J^{12}_{5,3} - J^{12}_{5,5} - J^{12}_{5,6} - J^{12}_{5,7} - J^{12}_{5,9} - J^{12}_{5,10} - J^{12}_{5,11} + 2K \\
(A_{\mathbf{k}})_{5,6} &= \Big[(A_{\mathbf{k}})_{6,5}\Big]^* = -J^1_{5,6},\ (A_{\mathbf{k}})_{5,8} = \Big[(A_{\mathbf{k}})_{8,5}\Big]^* = -J^1_{5,8}e^{i\mathbf{k}\cdot\delta_2},\ (A_{\mathbf{k}})_{5,9} = \Big[(A_{\mathbf{k}})_{9,5}\Big]^* = -J^1_{5,9} \\
(A_{\mathbf{k}})_{5,10} &= \Big[(A_{\mathbf{k}})_{10,5}\Big]^* = -J^1_{5,10},\ (A_{\mathbf{k}})_{5,12} = \Big[(A_{\mathbf{k}})_{12,5}\Big]^* = -J^1_{5,12}e^{i\mathbf{k}\cdot\delta_2} \\
(A_{\mathbf{k}})_{6,6} &= J^1_{6,1} + J^1_{6,2} + J^1_{6,3} + J^1_{6,5} + J^1_{6,7} + J^1_{6,9} + J^1_{6,10} + J^1_{6,11} \\
&- J^{12}_{6,6} - J^{12}_{6,9} - J^{12}_{6,10} - J^{12}_{6,11} - J^{12}_{6,13} - J^{12}_{6,14} - J^{12}_{6,15} - J^{12}_{6,18} + 2K \\
(A_{\mathbf{k}})_{6,7} &= \Big[(A_{\mathbf{k}})_{7,6}\Big]^* = -J^1_{6,7},\ (A_{\mathbf{k}})_{6,9} = \Big[(A_{\mathbf{k}})_{9,6}\Big]^* = -J^1_{6,9} \\
(A_{\mathbf{k}})_{6,10} &= \Big[(A_{\mathbf{k}})_{10,6}\Big]^* = -J^1_{6,10},\ (A_{\mathbf{k}})_{6,11} = \Big[(A_{\mathbf{k}})_{11,6}\Big]^* = -J^1_{6,11}
\end{aligned}
$$

$$
\begin{aligned}
(A_{\mathbf{k}})_{7,7} &= J^1_{7,2} + J^1_{7,3} + J^1_{7,4} + J^1_{7,6} + J^1_{7,8} + J^1_{7,10} + J^1_{7,11} + J^1_{7,12} \\
&- J^{12}_{7,13} - J^{12}_{7,14} - J^{12}_{7,15} - J^{12}_{7,17} - J^{12}_{7,18} - J^{12}_{7,19} - J^{12}_{7,21} - J^{12}_{7,22} - J^{12}_{7,23} + 2K \\
(A_{\mathbf{k}})_{7,8} &= \Big[(A_{\mathbf{k}})_{8,7}\Big]^* = -J^1_{7,8},\ (A_{\mathbf{k}})_{7,10} = \Big[(A_{\mathbf{k}})_{10,7}\Big]^* = -J^1_{7,10} \\
(A_{\mathbf{k}})_{7,11} &= \Big[(A_{\mathbf{k}})_{11,7}\Big]^* = -J^1_{7,11},\ (A_{\mathbf{k}})_{7,12} = \Big[(A_{\mathbf{k}})_{12,7}\Big]^* = -J^1_{7,12} \\
(A_{\mathbf{k}})_{8,8} &= J^1_{8,1} + J^1_{8,3} + J^1_{8,4} + J^1_{8,5} + J^1_{8,7} + J^1_{8,9} + J^1_{8,11} + J^1_{8,12} \\
&- J^{12}_{8,1} - J^{12}_{8,2} - J^{12}_{8,3} - J^{12}_{8,6} - J^{12}_{8,18} - J^{12}_{8,21} - J^{12}_{8,22} - J^{12}_{8,23} + 2K \\
(A_{\mathbf{k}})_{8,9} &= \Big[(A_{\mathbf{k}})_{9,8}\Big]^* = -J^1_{8,9}e^{i\mathbf{k}\cdot\delta_1},\ (A_{\mathbf{k}})_{8,11} = \Big[(A_{\mathbf{k}})_{11,8}\Big]^* = -J^1_{8,11},\ (A_{\mathbf{k}})_{8,12} = \Big[(A_{\mathbf{k}})_{12,8}\Big]^* = -J^1_{8,12} \\
(A_{\mathbf{k}})_{9,9} &= J^1_{9,5} + J^1_{9,6} + J^1_{9,8} + J^1_{9,10} + J^1_{9,12} + J^1_{9,13} + J^1_{9,14} + J^1_{9,16} \\
&- J^{12}_{9,2} - J^{12}_{9,3} - J^{12}_{9,6} - J^{12}_{9,7} - J^{12}_{9,10} - J^{12}_{9,11} + 2K \\
(A_{\mathbf{k}})_{9,10} &= \Big[(A_{\mathbf{k}})_{10,9}\Big]^* = -J^1_{9,10},\ (A_{\mathbf{k}})_{9,12} = \Big[(A_{\mathbf{k}})_{12,9}\Big]^* = -J^1_{9,12}e^{i\mathbf{k}\cdot\delta_2},\ (A_{\mathbf{k}})_{9,13} = \Big[(A_{\mathbf{k}})_{13,9}\Big]^* = -J^1_{9,13} \\
(A_{\mathbf{k}})_{9,14} &= \Big[(A_{\mathbf{k}})_{14,9}\Big]^* = -J^1_{9,14},\ (A_{\mathbf{k}})_{9,16} = \Big[(A_{\mathbf{k}})_{16,9}\Big]^* = -J^1_{9,16}e^{i\mathbf{k}\cdot\delta_2} \\
(A_{\mathbf{k}})_{10,10} &= J^1_{10,5} + J^1_{10,6} + J^1_{10,7} + J^1_{10,9} + J^1_{10,11} + J^1_{10,13} + J^1_{10,14} + J^1_{10,15} \\
&- J^{12}_{10,6} - J^{12}_{10,7} - J^{12}_{10,10} - J^{12}_{10,11} - J^{12}_{10,14} - J^{12}_{10,15} - J^{12}_{10,18} - J^{12}_{10,19} + 2K \\
(A_{\mathbf{k}})_{10,11} &= \Big[(A_{\mathbf{k}})_{11,10}\Big]^* = -J^1_{10,11},\ (A_{\mathbf{k}})_{10,13} = \Big[(A_{\mathbf{k}})_{13,10}\Big]^* = -J^1_{10,13} \\
(A_{\mathbf{k}})_{10,14} &= \Big[(A_{\mathbf{k}})_{14,10}\Big]^* = -J^1_{10,14},\ (A_{\mathbf{k}})_{10,15} = \Big[(A_{\mathbf{k}})_{15,10}\Big]^* = -J^1_{10,15} \\
(A_{\mathbf{k}})_{11,11} &= J^1_{11,6} + J^1_{11,7} + J^1_{11,8} + J^1_{11,10} + J^1_{11,12} + J^1_{11,14} + J^1_{11,15} + J^1_{11,16} \\
&- J^{12}_{11,14} - J^{12}_{11,15} - J^{12}_{11,18} - J^{12}_{11,19} - J^{12}_{11,22} - J^{12}_{11,23} + 2K \\
(A_{\mathbf{k}})_{11,12} &= \Big[(A_{\mathbf{k}})_{12,11}\Big]^* = -J^1_{11,12},\ (A_{\mathbf{k}})_{11,14} = \Big[(A_{\mathbf{k}})_{14,11}\Big]^* = -J^1_{11,14} \\
(A_{\mathbf{k}})_{11,15} &= \Big[(A_{\mathbf{k}})_{15,11}\Big]^* = -J^1_{11,15},\ (A_{\mathbf{k}})_{11,16} = \Big[(A_{\mathbf{k}})_{16,11}\Big]^* = -J^1_{11,16} \\
(A_{\mathbf{k}})_{12,12} &= J^1_{12,5} + J^1_{12,7} + J^1_{12,8} + J^1_{12,9} + J^1_{12,11} + J^1_{12,13} + J^1_{12,15} + J^1_{12,16} \\
&- J^{12}_{12,2} - J^{12}_{12,3} - J^{12}_{12,6} - J^{12}_{12,7} - J^{12}_{12,18} - J^{12}_{12,19} - J^{12}_{12,22} - J^{12}_{12,23} + 2K \\
(A_{\mathbf{k}})_{12,13} &= \Big[(A_{\mathbf{k}})_{13,12}\Big]^* = -J^1_{12,13}e^{i\mathbf{k}\cdot\delta_1},\ (A_{\mathbf{k}})_{12,15} = \Big[(A_{\mathbf{k}})_{15,12}\Big]^* = -J^1_{12,15} \\
(A_{\mathbf{k}})_{12,16} &= \Big[(A_{\mathbf{k}})_{16,12}\Big]^* = -J^1_{12,16} \\
(A_{\mathbf{k}})_{13,13} &= J^1_{13,9} + J^1_{13,10} + J^1_{13,12} + J^1_{13,14} + J^1_{13,16} + J^1_{13,17} + J^1_{13,18} + J^1_{13,20} \\
&- J^{12}_{13,3} - J^{12}_{13,4} - J^{12}_{13,7} - J^{12}_{13,8} - J^{12}_{13,11} - J^{12}_{13,12} + 2K \\
(A_{\mathbf{k}})_{13,14} &= \Big[(A_{\mathbf{k}})_{14,13}\Big]^* = -J^1_{13,14},\ (A_{\mathbf{k}})_{13,16} = \Big[(A_{\mathbf{k}})_{16,13}\Big]^* = -J^1_{13,16}e^{i\mathbf{k}\cdot\delta_2} \\
(A_{\mathbf{k}})_{13,17} &= \Big[(A_{\mathbf{k}})_{17,13}\Big]^* = -J^1_{13,17},\ (A_{\mathbf{k}})_{13,18} = \Big[(A_{\mathbf{k}})_{18,13}\Big]^* = -J^1_{13,18} \\
(A_{\mathbf{k}})_{13,20} &= \Big[(A_{\mathbf{k}})_{20,13}\Big]^* = -J^1_{13,20}e^{i\mathbf{k}\cdot\delta_2} \\
(A_{\mathbf{k}})_{14,14} &= J^1_{14,9} + J^1_{14,10} + J^1_{14,11} + J^1_{14,13} + J^1_{14,15} + J^1_{14,17} + J^1_{14,18} + J^1_{14,19} \\
&- J^{12}_{14,7} - J^{12}_{14,8} - J^{12}_{14,11} - J^{12}_{14,12} - J^{12}_{14,15} - J^{12}_{14,16} - J^{12}_{14,19} - J^{12}_{14,20} + 2K
\end{aligned}
$$

$$
\begin{aligned}
(A_{\mathbf{k}})_{14,15} &= \Big[(A_{\mathbf{k}})_{15,14}\Big]^* = -J^1_{14,15},\ (A_{\mathbf{k}})_{14,17} = \Big[(A_{\mathbf{k}})_{17,14}\Big]^* = -J^1_{14,17} \\
(A_{\mathbf{k}})_{14,18} &= \Big[(A_{\mathbf{k}})_{18,14}\Big]^* = -J^1_{14,18},\ (A_{\mathbf{k}})_{14,19} = \Big[(A_{\mathbf{k}})_{19,14}\Big]^* = -J^1_{14,19} \\
(A_{\mathbf{k}})_{15,15} &= J^1_{15,10} + J^1_{15,11} + J^1_{15,12} + J^1_{15,14} + J^1_{15,16} + J^1_{15,18} + J^1_{15,19} + J^1_{15,20} \\
&- J^{12}_{15,15} - J^{12}_{15,16} - J^{12}_{15,19} - J^{12}_{15,20} - J^{12}_{15,23} - J^{12}_{15,24} + 2K \\
(A_{\mathbf{k}})_{15,16} &= \Big[(A_{\mathbf{k}})_{16,15}\Big]^* = -J^1_{15,16},\ (A_{\mathbf{k}})_{15,18} = \Big[(A_{\mathbf{k}})_{18,15}\Big]^* = -J^1_{15,18} \\
(A_{\mathbf{k}})_{15,19} &= \Big[(A_{\mathbf{k}})_{19,15}\Big]^* = -J^1_{15,19},\ (A_{\mathbf{k}})_{15,20} = \Big[(A_{\mathbf{k}})_{20,15}\Big]^* = -J^1_{15,20} \\
(A_{\mathbf{k}})_{16,16} &= J^1_{16,9} + J^1_{16,11} + J^1_{16,12} + J^1_{16,13} + J^1_{16,15} + J^1_{16,17} + J^1_{16,19} + J^1_{16,20} \\
&- J^{12}_{16,3} - J^{12}_{16,4} - J^{12}_{16,7} - J^{12}_{16,8} - J^{12}_{16,19} - J^{12}_{16,20} - J^{12}_{16,23} - J^{12}_{16,24} + 2K \\
(A_{\mathbf{k}})_{16,17} &= \Big[(A_{\mathbf{k}})_{17,16}\Big]^* = -J^1_{16,17}e^{i\mathbf{k}\cdot\delta_1},\ (A_{\mathbf{k}})_{16,19} = \Big[(A_{\mathbf{k}})_{19,16}\Big]^* = -J^1_{16,19} \\
(A_{\mathbf{k}})_{16,20} &= \Big[(A_{\mathbf{k}})_{20,16}\Big]^* = -J^1_{16,20} \\
(A_{\mathbf{k}})_{17,17} &= J^1_{17,13} + J^1_{17,14} + J^1_{17,16} + J^1_{17,18} + J^1_{17,20} + J^1_{17,21} + J^1_{17,22} + J^1_{17,24} \\
&- J^{12}_{17,1} - J^{12}_{17,3} - J^{12}_{17,4} - J^{12}_{17,5} - J^{12}_{17,7} - J^{12}_{17,8} - J^{12}_{17,9} - J^{12}_{17,11} - J^{12}_{17,12} + 2K \\
(A_{\mathbf{k}})_{17,18} &= \Big[(A_{\mathbf{k}})_{18,17}\Big]^* = -J^1_{17,18},\ (A_{\mathbf{k}})_{17,20} = \Big[(A_{\mathbf{k}})_{20,17}\Big]^* = -J^1_{17,20}e^{i\mathbf{k}\cdot\delta_2} \\
(A_{\mathbf{k}})_{17,21} &= \Big[(A_{\mathbf{k}})_{21,17}\Big]^* = -J^1_{17,21},\ (A_{\mathbf{k}})_{17,22} = \Big[(A_{\mathbf{k}})_{22,17}\Big]^* = -J^1_{17,22} \\
(A_{\mathbf{k}})_{17,24} &= \Big[(A_{\mathbf{k}})_{24,17}\Big]^* = -J^1_{17,24}e^{i\mathbf{k}\cdot\delta_2} \\
(A_{\mathbf{k}})_{18,18} &= J^1_{18,13} + J^1_{18,14} + J^1_{18,15} + J^1_{18,17} + J^1_{18,19} + J^1_{18,21} + J^1_{18,22} + J^1_{18,23} \\
&- J^{12}_{18,8} - J^{12}_{18,9} - J^{12}_{18,11} - J^{12}_{18,12} - J^{12}_{18,13} - J^{12}_{18,15} - J^{12}_{18,16} - J^{12}_{18,20} + 2K \\
(A_{\mathbf{k}})_{18,19} &= \Big[(A_{\mathbf{k}})_{19,18}\Big]^* = -J^1_{18,19},\ (A_{\mathbf{k}})_{18,21} = \Big[(A_{\mathbf{k}})_{21,18}\Big]^* = -J^1_{18,21} \\
(A_{\mathbf{k}})_{18,22} &= \Big[(A_{\mathbf{k}})_{22,18}\Big]^* = -J^1_{18,22},\ (A_{\mathbf{k}})_{18,23} = \Big[(A_{\mathbf{k}})_{23,18}\Big]^* = -J^1_{18,23} \\
(A_{\mathbf{k}})_{19,19} &= J^1_{19,14} + J^1_{19,15} + J^1_{19,16} + J^1_{19,18} + J^1_{19,20} + J^1_{19,22} + J^1_{19,23} + J^1_{19,24} \\
&- J^{12}_{19,13} - J^{12}_{19,15} - J^{12}_{19,16} - J^{12}_{19,17} - J^{12}_{19,19} - J^{12}_{19,20} - J^{12}_{19,21} - J^{12}_{19,23} - J^{12}_{19,24} + 2K \\
(A_{\mathbf{k}})_{19,20} &= \Big[(A_{\mathbf{k}})_{20,19}\Big]^* = -J^1_{19,20},\ (A_{\mathbf{k}})_{19,22} = \Big[(A_{\mathbf{k}})_{22,19}\Big]^* = -J^1_{19,22} \\
(A_{\mathbf{k}})_{19,23} &= \Big[(A_{\mathbf{k}})_{23,19}\Big]^* = -J^1_{19,23},\ (A_{\mathbf{k}})_{19,24} = \Big[(A_{\mathbf{k}})_{24,19}\Big]^* = -J^1_{19,24} \\
(A_{\mathbf{k}})_{20,20} &= J^1_{20,13} + J^1_{20,15} + J^1_{20,16} + J^1_{20,17} + J^1_{20,19} + J^1_{20,21} + J^1_{20,23} + J^1_{20,24} \\
&- J^{12}_{20,1} - J^{12}_{20,3} - J^{12}_{20,4} - J^{12}_{20,8} - J^{12}_{20,20} - J^{12}_{20,21} - J^{12}_{20,23} - J^{12}_{20,24} + 2K \\
(A_{\mathbf{k}})_{20,21} &= \Big[(A_{\mathbf{k}})_{21,20}\Big]^* = -J^1_{20,21}e^{i\mathbf{k}\cdot\delta_1},\ (A_{\mathbf{k}})_{20,23} = \Big[(A_{\mathbf{k}})_{23,20}\Big]^* = -J^1_{20,23} \\
(A_{\mathbf{k}})_{20,24} &= \Big[(A_{\mathbf{k}})_{24,20}\Big]^* = -J^1_{20,24} \\
(A_{\mathbf{k}})_{21,21} &= J^1_{21,1} + J^1_{21,2} + J^1_{21,4} + J^1_{21,17} + J^1_{21,18} + J^1_{21,20} + J^1_{21,22} + J^1_{21,24} \\
&- J^{12}_{21,1} - J^{12}_{21,4} - J^{12}_{21,5} - J^{12}_{21,8} - J^{12}_{21,9} - J^{12}_{21,12} + 2K
\end{aligned}
$$

$$
\begin{aligned}
(A_{\mathbf{k}})_{21,22} &= \left[(A_{\mathbf{k}})_{22,21}\right]^* = -J^1_{21,22},\ (A_{\mathbf{k}})_{21,24} = \left[(A_{\mathbf{k}})_{24,21}\right]^* = -J^1_{21,24}e^{i\mathbf{k}\cdot\delta_2} \\
(A_{\mathbf{k}})_{22,22} &= J^1_{22,1} + J^1_{22,2} + J^1_{22,3} + J^1_{22,17} + J^1_{22,18} + J^1_{22,19} + J^1_{22,21} + J^1_{22,23} \\
&- J^{12}_{22,5} - J^{12}_{22,8} - J^{12}_{22,9} - J^{12}_{22,12} - J^{12}_{22,13} - J^{12}_{22,16} - J^{12}_{22,17} - J^{12}_{22,20} + 2K \\
(A_{\mathbf{k}})_{22,23} &= \left[(A_{\mathbf{k}})_{23,22}\right]^* = -J^1_{22,23} \\
(A_{\mathbf{k}})_{23,23} &= J^1_{23,2} + J^1_{23,3} + J^1_{23,4} + J^1_{23,18} + J^1_{23,19} + J^1_{23,20} + J^1_{23,22} + J^1_{23,24} \\
&- J^{12}_{23,13} - J^{12}_{23,16} - J^{12}_{23,17} - J^{12}_{23,20} - J^{12}_{23,21} - J^{12}_{23,24} + 2K \\
(A_{\mathbf{k}})_{23,24} &= \left[(A_{\mathbf{k}})_{24,23}\right]^* = -J^1_{23,24} \\
(A_{\mathbf{k}})_{24,24} &= J^1_{24,1} + J^1_{24,3} + J^1_{24,4} + J^1_{24,17} + J^1_{24,19} + J^1_{24,20} + J^1_{24,21} + J^1_{24,23} \\
&- J^{12}_{24,1} - J^{12}_{24,4} - J^{12}_{24,5} - J^{12}_{24,8} - J^{12}_{24,17} - J^{12}_{24,20} - J^{12}_{24,21} - J^{12}_{24,24} + 2K \\
(B_{\mathbf{k}})_{1,1} &= J^2_{1,2} + J^2_{1,4} + J^2_{1,5} + J^2_{1,6} + J^2_{1,8} + J^2_{1,21} + J^2_{1,22} + J^2_{1,24} \\
&- J^{12}_{1,1} - J^{12}_{4,1} - J^{12}_{5,1} - J^{12}_{8,1} - J^{12}_{17,1} - J^{12}_{20,1} - J^{12}_{21,1} - J^{12}_{24,1} + 2K \\
(B_{\mathbf{k}})_{1,2} &= \left[(B_{\mathbf{k}})_{2,1}\right]^* = -J^2_{1,2},\ (B_{\mathbf{k}})_{1,4} = \left[(B_{\mathbf{k}})_{4,1}\right]^* = -J^2_{1,4}e^{i\mathbf{k}\cdot\delta_4},\ (B_{\mathbf{k}})_{1,5} = \left[(B_{\mathbf{k}})_{5,1}\right]^* = -J^2_{1,5} \\
(B_{\mathbf{k}})_{1,6} &= \left[(B_{\mathbf{k}})_{6,1}\right]^* = -J^2_{1,6},\ (B_{\mathbf{k}})_{1,8} = \left[(B_{\mathbf{k}})_{8,1}\right]^* = -J^2_{1,8}e^{i\mathbf{k}\cdot\delta_4}, (B_{\mathbf{k}})_{1,21} = \left[(B_{\mathbf{k}})_{21,1}\right]^* = -J^2_{1,21}e^{i\mathbf{k}\cdot\delta_2} \\
(B_{\mathbf{k}})_{1,22} &= \left[(B_{\mathbf{k}})_{22,1}\right]^* = -J^2_{1,22}e^{i\mathbf{k}\cdot\delta_2},\ (B_{\mathbf{k}})_{1,24} = \left[(B_{\mathbf{k}})_{24,1}\right]^* = -J^2_{1,24}e^{i\mathbf{k}\cdot\delta_6} \\
(B_{\mathbf{k}})_{2,2} &= J^2_{2,1} + J^2_{2,3} + J^2_{2,5} + J^2_{2,6} + J^2_{2,7} + J^2_{2,21} + J^2_{2,22} + J^2_{2,23} \\
&- J^{12}_{1,2} - J^{12}_{4,2} - J^{12}_{5,2} - J^{12}_{8,2} - J^{12}_{9,2} - J^{12}_{12,2} + 2K \\
(B_{\mathbf{k}})_{2,3} &= \left[(B_{\mathbf{k}})_{3,2}\right]^* = -J^2_{2,3},\ (B_{\mathbf{k}})_{2,5} = \left[(B_{\mathbf{k}})_{5,2}\right]^* = -J^2_{2,5},\ (B_{\mathbf{k}})_{2,6} = \left[(B_{\mathbf{k}})_{6,2}\right]^* = -J^2_{2,6} \\
(B_{\mathbf{k}})_{2,7} &= \left[(B_{\mathbf{k}})_{7,2}\right]^* = -J^2_{2,7},\ (B_{\mathbf{k}})_{2,21} = \left[(B_{\mathbf{k}})_{21,2}\right]^* = -J^2_{2,21}e^{i\mathbf{k}\cdot\delta_2} \\
(B_{\mathbf{k}})_{2,22} &= \left[(B_{\mathbf{k}})_{22,2}\right]^* = -J^2_{2,22}e^{i\mathbf{k}\cdot\delta_2},\ (B_{\mathbf{k}})_{2,23} = \left[(B_{\mathbf{k}})_{23,2}\right]^* = -J^2_{2,23}e^{i\mathbf{k}\cdot\delta_2} \\
(B_{\mathbf{k}})_{3,3} &= J^2_{3,2} + J^2_{3,4} + J^2_{3,6} + J^2_{3,7} + J^2_{3,8} + J^2_{3,22} + J^2_{3,23} + J^2_{3,24} \\
&- J^{12}_{5,3} - J^{12}_{8,3} - J^{12}_{9,3} - J^{12}_{12,3} - J^{12}_{13,3} - J^{12}_{16,3} - J^{12}_{17,3} - J^{12}_{20,3} + 2K \\
(B_{\mathbf{k}})_{3,4} &= \left[(B_{\mathbf{k}})_{4,3}\right]^* = -J^2_{3,4},\ (B_{\mathbf{k}})_{3,6} = \left[(B_{\mathbf{k}})_{6,3}\right]^* = -J^2_{3,6},\ (B_{\mathbf{k}})_{3,7} = \left[(B_{\mathbf{k}})_{7,3}\right]^* = -J^2_{3,7} \\
(B_{\mathbf{k}})_{3,8} &= \left[(B_{\mathbf{k}})_{8,3}\right]^* = -J^2_{3,8},\ (B_{\mathbf{k}})_{3,22} = \left[(B_{\mathbf{k}})_{22,3}\right]^* = -J^2_{3,22}e^{i\mathbf{k}\cdot\delta_2} \\
(B_{\mathbf{k}})_{3,23} &= \left[(B_{\mathbf{k}})_{23,3}\right]^* = -J^2_{3,23}e^{i\mathbf{k}\cdot\delta_2},\ (B_{\mathbf{k}})_{3,24} = \left[(B_{\mathbf{k}})_{24,3}\right]^* = -J^2_{3,24}e^{i\mathbf{k}\cdot\delta_2} \\
(B_{\mathbf{k}})_{4,4} &= J^2_{4,1} + J^2_{4,3} + J^2_{4,5} + J^2_{4,7} + J^2_{4,8} + J^2_{4,21} + J^2_{4,23} + J^2_{4,24} \\
&- J^{12}_{13,4} - J^{12}_{16,4} - J^{12}_{17,4} - J^{12}_{20,4} - J^{12}_{21,4} - J^{12}_{24,4} + 2K \\
(B_{\mathbf{k}})_{4,5} &= \left[(B_{\mathbf{k}})_{5,4}\right]^* = -J^2_{4,5}e^{i\mathbf{k}\cdot\delta_3},\ (B_{\mathbf{k}})_{4,7} = \left[(B_{\mathbf{k}})_{7,4}\right]^* = -J^2_{4,7},\ (B_{\mathbf{k}})_{4,8} = \left[(B_{\mathbf{k}})_{8,4}\right]^* = -J^2_{4,8} \\
(B_{\mathbf{k}})_{4,21} &= \left[(B_{\mathbf{k}})_{21,4}\right]^* = -J^2_{4,21}e^{i\mathbf{k}\cdot\delta_8},\ (B_{\mathbf{k}})_{4,23} = \left[(B_{\mathbf{k}})_{23,4}\right]^* = -J^2_{4,23}e^{i\mathbf{k}\cdot\delta_2} \\
(B_{\mathbf{k}})_{4,24} &= \left[(B_{\mathbf{k}})_{24,4}\right]^* = -J^2_{4,24}e^{i\mathbf{k}\cdot\delta_2}
\end{aligned}
$$

$$
\begin{aligned}
(B_{\mathbf{k}})_{5,5} &= J^2_{5,1} + J^2_{5,2} + J^2_{5,4} + J^2_{5,6} + J^2_{5,8} + J^2_{5,9} + J^2_{5,10} + J^2_{5,12} \\
&- J^{12}_{1,5} - J^{12}_{2,5} - J^{12}_{4,5} - J^{12}_{5,5} - J^{12}_{17,5} - J^{12}_{21,5} - J^{12}_{22,5} - J^{12}_{24,5} + 2K \\
(B_{\mathbf{k}})_{5,6} &= \Big[(B_{\mathbf{k}})_{6,5}\Big]^* = -J^2_{5,6},\ (B_{\mathbf{k}})_{5,8} = \Big[(B_{\mathbf{k}})_{8,5}\Big]^* = -J^2_{5,8}e^{i\mathbf{k}\cdot\delta_4},\ (B_{\mathbf{k}})_{5,9} = \Big[(B_{\mathbf{k}})_{9,5}\Big]^* = -J^2_{5,9} \\
(B_{\mathbf{k}})_{5,10} &= \Big[(B_{\mathbf{k}})_{10,5}\Big]^* = -J^2_{5,10},\ (B_{\mathbf{k}})_{5,12} = \Big[(B_{\mathbf{k}})_{12,5}\Big]^* = -J^2_{5,12}e^{i\mathbf{k}\cdot\delta_4} \\
(B_{\mathbf{k}})_{6,6} &= J^2_{6,1} + J^2_{6,2} + J^2_{6,3} + J^2_{6,5} + J^2_{6,7} + J^2_{6,9} + J^2_{6,10} + J^2_{6,11} \\
&- J^{12}_{1,6} - J^{12}_{2,6} - J^{12}_{4,6} - J^{12}_{5,6} - J^{12}_{6,6} - J^{12}_{8,6} - J^{12}_{9,6} - J^{12}_{10,6} - J^{12}_{12,6} + 2K \\
(B_{\mathbf{k}})_{6,7} &= \Big[(B_{\mathbf{k}})_{7,6}\Big]^* = -J^2_{6,7},\ (B_{\mathbf{k}})_{6,9} = \Big[(B_{\mathbf{k}})_{9,6}\Big]^* = -J^2_{6,9} \\
(B_{\mathbf{k}})_{6,10} &= \Big[(B_{\mathbf{k}})_{10,6}\Big]^* = -J^2_{6,10},\ (B_{\mathbf{k}})_{6,11} = \Big[(B_{\mathbf{k}})_{11,6}\Big]^* = -J^2_{6,11} \\
(B_{\mathbf{k}})_{7,7} &= J^2_{7,2} + J^2_{7,3} + J^2_{7,4} + J^2_{7,6} + J^2_{7,8} + J^2_{7,10} + J^2_{7,11} + J^2_{7,12} \\
&- J^{12}_{5,7} - J^{12}_{9,7} - J^{12}_{10,7} - J^{12}_{12,7} - J^{12}_{13,7} - J^{12}_{14,7} - J^{12}_{16,7} - J^{12}_{17,7} + 2K \\
(B_{\mathbf{k}})_{7,8} &= \Big[(B_{\mathbf{k}})_{8,7}\Big]^* = -J^2_{7,8},\ (B_{\mathbf{k}})_{7,10} = \Big[(B_{\mathbf{k}})_{10,7}\Big]^* = -J^2_{7,10} \\
(B_{\mathbf{k}})_{7,11} &= \Big[(B_{\mathbf{k}})_{11,7}\Big]^* = -J^2_{7,11},\ (B_{\mathbf{k}})_{7,12} = \Big[(B_{\mathbf{k}})_{12,7}\Big]^* = -J^2_{7,12} \\
(B_{\mathbf{k}})_{8,8} &= J^2_{8,1} + J^2_{8,3} + J^2_{8,4} + J^2_{8,5} + J^2_{8,7} + J^2_{8,9} + J^2_{8,11} + J^2_{8,12} \\
&- J^{12}_{13,8} - J^{12}_{14,8} - J^{12}_{16,8} - J^{12}_{17,8} - J^{12}_{18,8} - J^{12}_{20,8} - J^{12}_{21,8} - J^{12}_{22,8} - J^{12}_{24,8} + 2K \\
(B_{\mathbf{k}})_{8,9} &= \Big[(B_{\mathbf{k}})_{9,8}\Big]^* = -J^2_{8,9}e^{i\mathbf{k}\cdot\delta_3},\ (B_{\mathbf{k}})_{8,11} = \Big[(B_{\mathbf{k}})_{11,8}\Big]^* = -J^2_{8,11},\ (B_{\mathbf{k}})_{8,12} = \Big[(B_{\mathbf{k}})_{12,8}\Big]^* = -J^2_{8,12} \\
(B_{\mathbf{k}})_{9,9} &= J^2_{9,5} + J^1_{9,6} + J^1_{9,8} + J^1_{9,10} + J^1_{9,12} + J^1_{9,13} + J^1_{9,14} + J^1_{9,16} \\
&- J^{12}_{1,9} - J^{12}_{2,9} - J^{12}_{5,9} - J^{12}_{6,9} - J^{12}_{17,9} - J^{12}_{18,9} - J^{12}_{21,9} - J^{12}_{22,9} + 2K \\
(B_{\mathbf{k}})_{9,10} &= \Big[(B_{\mathbf{k}})_{10,9}\Big]^* = -J^2_{9,10},\ (B_{\mathbf{k}})_{9,12} = \Big[(B_{\mathbf{k}})_{12,9}\Big]^* = -J^2_{9,12}e^{i\mathbf{k}\cdot\delta_4} \\
(B_{\mathbf{k}})_{9,13} &= \Big[(B_{\mathbf{k}})_{13,9}\Big]^* = -J^2_{9,13},\ (B_{\mathbf{k}})_{9,14} = \Big[(B_{\mathbf{k}})_{14,9}\Big]^* = -J^2_{9,14}, (B_{\mathbf{k}})_{9,16} = \Big[(B_{\mathbf{k}})_{16,9}\Big]^* = -J^2_{9,16}e^{i\mathbf{k}\cdot\delta_4} \\
(B_{\mathbf{k}})_{10,10} &= J^2_{10,5} + J^2_{10,6} + J^2_{10,7} + J^2_{10,9} + J^2_{10,11} + J^2_{10,13} + J^2_{10,14} + J^2_{10,15} \\
&- J^{12}_{1,10} - J^{12}_{2,10} - J^{12}_{5,10} - J^{12}_{6,10} - J^{12}_{9,10} - J^{12}_{10,10} + 2K \\
(B_{\mathbf{k}})_{10,11} &= \Big[(B_{\mathbf{k}})_{11,10}\Big]^* = -J^2_{10,11},\ (B_{\mathbf{k}})_{10,13} = \Big[(B_{\mathbf{k}})_{13,10}\Big]^* = -J^2_{10,13} \\
(B_{\mathbf{k}})_{10,14} &= \Big[(B_{\mathbf{k}})_{14,10}\Big]^* = -J^2_{10,14},\ (B_{\mathbf{k}})_{10,15} = \Big[(B_{\mathbf{k}})_{15,10}\Big]^* = -J^2_{10,15} \\
(B_{\mathbf{k}})_{11,11} &= J^2_{11,6} + J^2_{11,7} + J^2_{11,8} + J^2_{11,10} + J^2_{11,12} + J^2_{11,14} + J^2_{11,15} + J^2_{11,16} \\
&- J^{12}_{5,11} - J^{12}_{6,11} - J^{12}_{9,11} - J^{12}_{10,11} - J^{12}_{13,11} - J^{12}_{14,11} - J^{12}_{17,11} - J^{12}_{18,11} + 2K \\
(B_{\mathbf{k}})_{11,12} &= \Big[(B_{\mathbf{k}})_{12,11}\Big]^* = -J^2_{11,12},\ (B_{\mathbf{k}})_{11,14} = \Big[(B_{\mathbf{k}})_{14,11}\Big]^* = -J^2_{11,14} \\
(B_{\mathbf{k}})_{11,15} &= \Big[(B_{\mathbf{k}})_{15,11}\Big]^* = -J^2_{11,15},\ (B_{\mathbf{k}})_{11,16} = \Big[(B_{\mathbf{k}})_{16,11}\Big]^* = -J^2_{11,16} \\
(B_{\mathbf{k}})_{12,12} &= J^2_{12,5} + J^2_{12,7} + J^2_{12,8} + J^2_{12,9} + J^2_{12,11} + J^2_{12,13} + J^2_{12,15} + J^2_{12,16} \\
&- J^{12}_{13,12} - J^{12}_{14,12} - J^{12}_{17,12} - J^{12}_{18,12} - J^{12}_{21,12} - J^{12}_{22,12} + 2K \\
(B_{\mathbf{k}})_{12,13} &= \Big[(B_{\mathbf{k}})_{13,12}\Big]^* = -J^2_{12,13}e^{i\mathbf{k}\cdot\delta_3},\ (B_{\mathbf{k}})_{12,15} = \Big[(B_{\mathbf{k}})_{15,12}\Big]^* = -J^2_{12,15}
\end{aligned}
$$

$$
\begin{aligned}
(B_{\mathbf{k}})_{12,16} &= \Big[(B_{\mathbf{k}})_{16,12}\Big]^* = -J^2_{12,16} \\
(B_{\mathbf{k}})_{13,13} &= J^2_{13,9} + J^2_{13,10} + J^2_{13,12} + J^2_{13,14} + J^2_{13,16} + J^2_{13,17} + J^2_{13,18} + J^2_{13,20} \\
&- J^{12}_{2,13} - J^{12}_{3,13} - J^{12}_{6,13} - J^{12}_{7,13} - J^{12}_{18,13} - J^{12}_{19,13} - J^{12}_{22,13} - J^{12}_{23,13} + 2K \\
(B_{\mathbf{k}})_{13,14} &= \Big[(B_{\mathbf{k}})_{14,13}\Big]^* = -J^2_{13,14},\ (B_{\mathbf{k}})_{13,16} = \Big[(B_{\mathbf{k}})_{16,13}\Big]^* = -J^2_{13,16}e^{i\mathbf{k}\cdot\delta_4} \\
(B_{\mathbf{k}})_{13,17} &= \Big[(B_{\mathbf{k}})_{17,13}\Big]^* = -J^2_{13,17},\ (B_{\mathbf{k}})_{13,18} = \Big[(B_{\mathbf{k}})_{18,13}\Big]^* = -J^2_{13,18} \\
(B_{\mathbf{k}})_{13,20} &= \Big[(B_{\mathbf{k}})_{20,13}\Big]^* = -J^2_{13,20}e^{i\mathbf{k}\cdot\delta_4} \\
(B_{\mathbf{k}})_{14,14} &= J^2_{14,9} + J^2_{14,10} + J^2_{14,11} + J^2_{14,13} + J^2_{14,15} + J^2_{14,17} + J^2_{14,18} + J^2_{14,19} \\
&- J^{12}_{2,14} - J^{12}_{3,14} - J^{12}_{6,14} - J^{12}_{7,14} - J^{12}_{10,14} - J^{12}_{11,14} + 2K \\
(B_{\mathbf{k}})_{14,15} &= \Big[(B_{\mathbf{k}})_{15,14}\Big]^* = -J^2_{14,15},\ (B_{\mathbf{k}})_{14,17} = \Big[(B_{\mathbf{k}})_{17,14}\Big]^* = -J^2_{14,17} \\
(B_{\mathbf{k}})_{14,18} &= \Big[(B_{\mathbf{k}})_{18,14}\Big]^* = -J^2_{14,18},\ (B_{\mathbf{k}})_{14,19} = \Big[(B_{\mathbf{k}})_{19,14}\Big]^* = -J^2_{14,19} \\
(B_{\mathbf{k}})_{15,15} &= J^2_{15,10} + J^2_{15,11} + J^2_{15,12} + J^2_{15,14} + J^2_{15,16} + J^2_{15,18} + J^2_{15,19} + J^2_{15,20} \\
&- J^{12}_{6,15} - J^{12}_{7,15} - J^{12}_{10,15} - J^{12}_{11,15} - J^{12}_{14,15} - J^{12}_{15,15} - J^{12}_{18,15} - J^{12}_{19,15} + 2K \\
(B_{\mathbf{k}})_{15,16} &= \Big[(B_{\mathbf{k}})_{16,15}\Big]^* = -J^2_{15,16},\ (B_{\mathbf{k}})_{15,18} = \Big[(B_{\mathbf{k}})_{18,15}\Big]^* = -J^2_{15,18} \\
(B_{\mathbf{k}})_{15,19} &= \Big[(B_{\mathbf{k}})_{19,15}\Big]^* = -J^2_{15,19},\ (B_{\mathbf{k}})_{15,20} = \Big[(B_{\mathbf{k}})_{20,15}\Big]^* = -J^2_{15,20} \\
(B_{\mathbf{k}})_{16,16} &= J^2_{16,9} + J^2_{16,11} + J^2_{16,12} + J^2_{16,13} + J^2_{16,15} + J^2_{16,17} + J^2_{16,19} + J^2_{16,20} \\
&- J^{12}_{14,16} - J^{12}_{15,16} - J^{12}_{18,16} - J^{12}_{19,16} - J^{12}_{22,16} - J^{12}_{23,16} + 2K \\
(B_{\mathbf{k}})_{16,17} &= \Big[(B_{\mathbf{k}})_{17,16}\Big]^* = -J^2_{16,17}e^{i\mathbf{k}\cdot\delta_3},\ (B_{\mathbf{k}})_{16,19} = \Big[(B_{\mathbf{k}})_{19,16}\Big]^* = -J^2_{16,19} \\
(B_{\mathbf{k}})_{16,20} &= \Big[(B_{\mathbf{k}})_{20,16}\Big]^* = -J^2_{16,20} \\
(B_{\mathbf{k}})_{17,17} &= J^2_{17,13} + J^2_{17,14} + J^2_{17,16} + J^2_{17,18} + J^2_{17,20} + J^2_{17,21} + J^2_{17,22} + J^2_{17,24} \\
&- J^{12}_{2,17} - J^{12}_{3,17} + -J^{12}_{4,17} - J^{12}_{7,17} - J^{12}_{19,17} - J^{12}_{22,17} - J^{12}_{23,17} - J^{12}_{24,17} + 2K \\
(B_{\mathbf{k}})_{17,18} &= \Big[(B_{\mathbf{k}})_{18,17}\Big]^* = -J^2_{17,18},\ (B_{\mathbf{k}})_{17,20} = \Big[(B_{\mathbf{k}})_{20,17}\Big]^* = -J^2_{17,20}e^{i\mathbf{k}\cdot\delta_4} \\
(B_{\mathbf{k}})_{17,21} &= \Big[(B_{\mathbf{k}})_{21,17}\Big]^* = -J^2_{17,21},\ (B_{\mathbf{k}})_{17,22} = \Big[(B_{\mathbf{k}})_{22,17}\Big]^* = -J^2_{17,22} \\
(B_{\mathbf{k}})_{17,24} &= \Big[(B_{\mathbf{k}})_{24,17}\Big]^* = -J^2_{17,24}e^{i\mathbf{k}\cdot\delta_4} \\
(B_{\mathbf{k}})_{18,18} &= J^2_{18,13} + J^2_{18,14} + J^2_{18,15} + J^2_{18,17} + J^2_{18,19} + J^2_{18,21} + J^2_{18,22} + J^2_{18,23} \\
&- J^{12}_{2,18} - J^{12}_{3,18} - J^{12}_{4,18} - J^{12}_{6,18} - J^{12}_{7,18} - J^{12}_{8,18} - J^{12}_{10,18} - J^{12}_{11,18} - J^{12}_{12,18} + 2K \\
(B_{\mathbf{k}})_{18,19} &= \Big[(B_{\mathbf{k}})_{19,18}\Big]^* = -J^2_{18,19},\ (B_{\mathbf{k}})_{18,21} = \Big[(B_{\mathbf{k}})_{21,18}\Big]^* = -J^2_{18,21} \\
(B_{\mathbf{k}})_{18,22} &= \Big[(B_{\mathbf{k}})_{22,18}\Big]^* = -J^2_{18,22},\ (B_{\mathbf{k}})_{18,23} = \Big[(B_{\mathbf{k}})_{23,18}\Big]^* = -J^2_{18,23} \\
(B_{\mathbf{k}})_{19,19} &= J^2_{19,14} + J^2_{19,15} + J^2_{19,16} + J^2_{19,18} + J^2_{19,20} + J^2_{19,22} + J^2_{19,23} + J^2_{19,24} \\
&- J^{12}_{7,19} - J^{12}_{10,19} - J^{12}_{11,19} - J^{12}_{12,19} - J^{12}_{14,19} - J^{12}_{15,19} - J^{12}_{16,19} - J^{12}_{19,19} + 2K
\end{aligned}
$$

$$
\begin{aligned}
(B_{\mathbf{k}})_{19,20} &= \Big[(B_{\mathbf{k}})_{20,19}\Big]^* = -J^2_{19,20},\ (B_{\mathbf{k}})_{19,22} = \Big[(B_{\mathbf{k}})_{22,19}\Big]^* = -J^2_{19,22} \\
(B_{\mathbf{k}})_{19,23} &= \Big[(B_{\mathbf{k}})_{23,19}\Big]^* = -J^2_{19,23},\ (B_{\mathbf{k}})_{19,24} = \Big[(B_{\mathbf{k}})_{24,19}\Big]^* = -J^2_{19,24} \\
(B_{\mathbf{k}})_{20,20} &= J^2_{20,13} + J^2_{20,15} + J^2_{20,16} + J^2_{20,17} + J^2_{20,19} + J^2_{20,21} + J^2_{20,23} + J^2_{20,24} \\
&- J^{12}_{14,20} - J^{12}_{15,20} - J^{12}_{16,20} - J^{12}_{18,20} - J^{12}_{19,20} - J^{12}_{20,20} - J^{12}_{22,20} - J^{12}_{23,20} - J^{12}_{24,20} + 2K \\
(B_{\mathbf{k}})_{20,21} &= \Big[(B_{\mathbf{k}})_{21,20}\Big]^* = -J^2_{20,21}e^{i\mathbf{k}\cdot\delta_3},\ (B_{\mathbf{k}})_{20,23} = \Big[(B_{\mathbf{k}})_{23,20}\Big]^* = -J^2_{20,23} \\
(B_{\mathbf{k}})_{20,24} &= \Big[(B_{\mathbf{k}})_{24,20}\Big]^* = -J^2_{20,24} \\
(B_{\mathbf{k}})_{21,21} &= J^2_{21,1} + J^2_{21,2} + J^2_{21,4} + J^2_{21,17} + J^2_{21,18} + J^2_{21,20} + J^2_{21,22} + J^2_{21,24} \\
&- J^{12}_{3,21} - J^{12}_{4,21} - J^{12}_{7,21} - J^{12}_{8,21} - J^{12}_{19,21} - J^{12}_{20,21} - J^{12}_{23,21} - J^{12}_{24,21} + 2K \\
(B_{\mathbf{k}})_{21,22} &= \Big[(B_{\mathbf{k}})_{22,21}\Big]^* = -J^2_{21,22},\ (B_{\mathbf{k}})_{21,24} = \Big[(B_{\mathbf{k}})_{24,21}\Big]^* = -J^2_{21,24}e^{i\mathbf{k}\cdot\delta_4} \\
(B_{\mathbf{k}})_{22,22} &= J^2_{22,1} + J^2_{22,2} + J^2_{22,3} + J^2_{22,17} + J^2_{22,18} + J^2_{22,19} + J^2_{22,21} + J^2_{22,23} \\
&- J^{12}_{3,22} - J^{12}_{4,22} - J^{12}_{7,22} - J^{12}_{8,22} - J^{12}_{11,22} - J^{12}_{12,22} + 2K \\
(B_{\mathbf{k}})_{22,23} &= \Big[(B_{\mathbf{k}})_{23,22}\Big]^* = -J^2_{22,23} \\
(B_{\mathbf{k}})_{23,23} &= J^2_{23,2} + J^2_{23,3} + J^2_{23,4} + J^2_{23,18} + J^2_{23,19} + J^2_{23,20} + J^2_{23,22} + J^2_{23,24} \\
&- J^{12}_{7,23} - J^{12}_{8,23} - J^{12}_{11,23} - J^{12}_{12,23} - J^{12}_{15,23} - J^{12}_{16,23} - J^{12}_{19,23} - J^{12}_{20,23} + 2K \\
(B_{\mathbf{k}})_{23,24} &= \Big[(B_{\mathbf{k}})_{24,23}\Big]^* = -J^2_{23,24} \\
(B_{\mathbf{k}})_{24,24} &= J^2_{24,1} + J^2_{24,3} + J^2_{24,4} + J^2_{24,17} + J^2_{24,19} + J^2_{24,20} + J^2_{24,21} + J^2_{24,23} \\
&- J^{12}_{15,24} - J^{12}_{16,24} - J^{12}_{19,24} - J^{12}_{20,24} - J^{12}_{23,24} - J^{12}_{24,24} + 2K \\
(C_{\mathbf{k}})_{1,1} &= -J^{12}_{1,1},\ (C_{\mathbf{k}})_{1,2} = -J^{12}_{1,2},\ (C_{\mathbf{k}})_{1,5} = -J^{12}_{1,5}, \\
(C_{\mathbf{k}})_{1,6} &= -J^{12}_{1,6},\ (C_{\mathbf{k}})_{1,9} = -J^{12}_{1,9},\ (C_{\mathbf{k}})_{1,10} = -J^{12}_{1,10}, \\
(C_{\mathbf{k}})_{2,5} &= -J^{12}_{2,5},\ (C_{\mathbf{k}})_{2,6} = -J^{12}_{2,6},\ (C_{\mathbf{k}})_{2,9} = -J^{12}_{2,9},\ (C_{\mathbf{k}})_{2,10} = -J^{12}_{2,10}, \\
(C_{\mathbf{k}})_{2,13} &= -J^{12}_{2,13},\ (C_{\mathbf{k}})_{2,14} = -J^{12}_{2,14},\ (C_{\mathbf{k}})_{2,17} = -J^{12}_{2,17},\ (C_{\mathbf{k}})_{2,18} = -J^{12}_{2,18}, \\
(C_{\mathbf{k}})_{3,13} &= -J^{12}_{3,13},\ (C_{\mathbf{k}})_{3,14} = -J^{12}_{3,14},\ (C_{\mathbf{k}})_{3,17} = -J^{12}_{3,17}, \\
(C_{\mathbf{k}})_{3,18} &= -J^{12}_{3,18},\ (C_{\mathbf{k}})_{3,21} = -J^{12}_{3,21},\ (C_{\mathbf{k}})_{3,22} = -J^{12}_{3,22}, \\
(C_{\mathbf{k}})_{4,1} &= -J^{12}_{4,1}e^{i\mathbf{k}\cdot\delta_1},\ (C_{\mathbf{k}})_{4,2} = -J^{12}_{4,2}e^{i\mathbf{k}\cdot\delta_1},\ (C_{\mathbf{k}})_{4,5} = -J^{12}_{4,5}e^{i\mathbf{k}\cdot\delta_1},\ (C_{\mathbf{k}})_{4,6} = -J^{12}_{4,6}e^{i\mathbf{k}\cdot\delta_1}, \\
(C_{\mathbf{k}})_{4,17} &= -J^{12}_{4,17},\ (C_{\mathbf{k}})_{4,18} = -J^{12}_{4,18},\ (C_{\mathbf{k}})_{4,21} = -J^{12}_{4,21},\ (C_{\mathbf{k}})_{4,22} = -J^{12}_{4,22}, \\
(C_{\mathbf{k}})_{5,1} &= -J^{12}_{5,1},\ (C_{\mathbf{k}})_{5,2} = -J^{12}_{5,2},\ (C_{\mathbf{k}})_{5,3} = -J^{12}_{5,3}, \\
(C_{\mathbf{k}})_{5,5} &= -J^{12}_{5,5},\ (C_{\mathbf{k}})_{5,6} = -J^{12}_{5,6},\ (C_{\mathbf{k}})_{5,7} = -J^{12}_{5,7}, \\
(C_{\mathbf{k}})_{5,9} &= -J^{12}_{5,9},\ (C_{\mathbf{k}})_{5,10} = -J^{12}_{5,10},\ (C_{\mathbf{k}})_{5,11} = -J^{12}_{5,11}, \\
(C_{\mathbf{k}})_{6,6} &= -J^{12}_{6,6},\ (C_{\mathbf{k}})_{6,9} = -J^{12}_{6,9},\ (C_{\mathbf{k}})_{6,10} = -J^{12}_{6,10},\ (C_{\mathbf{k}})_{6,11} = -J^{12}_{6,11}, \\
(C_{\mathbf{k}})_{6,13} &= -J^{12}_{6,13},\ (C_{\mathbf{k}})_{6,14} = -J^{12}_{6,14},\ (C_{\mathbf{k}})_{6,15} = -J^{12}_{6,15},\ (C_{\mathbf{k}})_{6,18} = -J^{12}_{6,18},
\end{aligned}
$$

$$
\begin{aligned}
(C_{\mathbf{k}})_{7,13} &= -J^{12}_{7,13},\ (C_{\mathbf{k}})_{7,14} = -J^{12}_{7,14},\ (C_{\mathbf{k}})_{7,15} = -J^{12}_{7,15},\\
(C_{\mathbf{k}})_{7,17} &= -J^{12}_{7,17},\ (C_{\mathbf{k}})_{7,18} = -J^{12}_{7,18},\ (C_{\mathbf{k}})_{7,19} = -J^{12}_{7,19},\\
(C_{\mathbf{k}})_{7,21} &= -J^{12}_{7,21},\ (C_{\mathbf{k}})_{7,22} = -J^{12}_{7,22},\ (C_{\mathbf{k}})_{7,23} = -J^{12}_{7,23},\\
(C_{\mathbf{k}})_{8,1} &= -J^{12}_{8,1}e^{i\mathbf{k}\cdot\delta_1},\ (C_{\mathbf{k}})_{8,2} = -J^{12}_{8,2}e^{i\mathbf{k}\cdot\delta_1},\ (C_{\mathbf{k}})_{8,3} = -J^{12}_{8,3}e^{i\mathbf{k}\cdot\delta_1},\ (C_{\mathbf{k}})_{8,6} = -J^{12}_{8,6}e^{i\mathbf{k}\cdot\delta_1},\\
(C_{\mathbf{k}})_{8,18} &= -J^{12}_{8,18},\ (C_{\mathbf{k}})_{8,21} = -J^{12}_{8,21},\ (C_{\mathbf{k}})_{8,22} = -J^{12}_{8,22},\ (C_{\mathbf{k}})_{8,23} = -J^{12}_{8,23},\\
(C_{\mathbf{k}})_{9,2} &= -J^{12}_{9,2},\ (C_{\mathbf{k}})_{9,3} = -J^{12}_{9,3},\ (C_{\mathbf{k}})_{9,6} = -J^{12}_{9,6},\\
(C_{\mathbf{k}})_{9,7} &= -J^{12}_{9,7},\ (C_{\mathbf{k}})_{9,10} = -J^{12}_{9,10},\ (C_{\mathbf{k}})_{9,11} = -J^{12}_{9,11},\\
(C_{\mathbf{k}})_{10,6} &= -J^{12}_{10,6},\ (C_{\mathbf{k}})_{10,7} = -J^{12}_{10,7},\ (C_{\mathbf{k}})_{10,10} = -J^{12}_{10,10},\ (C_{\mathbf{k}})_{10,11} = -J^{12}_{10,11},\\
(C_{\mathbf{k}})_{10,14} &= -J^{12}_{10,14},\ (C_{\mathbf{k}})_{10,15} = -J^{12}_{10,15},\ (C_{\mathbf{k}})_{10,18} = -J^{12}_{10,18},\ (C_{\mathbf{k}})_{10,19} = -J^{12}_{10,19},\\
(C_{\mathbf{k}})_{11,14} &= -J^{12}_{11,14},\ (C_{\mathbf{k}})_{11,15} = -J^{12}_{11,15},\ (C_{\mathbf{k}})_{11,18} = -J^{12}_{11,18},\\
(C_{\mathbf{k}})_{11,19} &= -J^{12}_{11,19},\ (C_{\mathbf{k}})_{11,22} = -J^{12}_{11,22},\ (C_{\mathbf{k}})_{11,23} = -J^{12}_{11,23},\\
(C_{\mathbf{k}})_{12,2} &= -J^{12}_{12,2}e^{i\mathbf{k}\cdot\delta_1},\ (C_{\mathbf{k}})_{12,3} = -J^{12}_{12,3}e^{i\mathbf{k}\cdot\delta_1},\ (C_{\mathbf{k}})_{12,6} = -J^{12}_{12,6}e^{i\mathbf{k}\cdot\delta_1},\ (C_{\mathbf{k}})_{12,7} = -J^{12}_{12,7}e^{i\mathbf{k}\cdot\delta_1},\\
(C_{\mathbf{k}})_{12,18} &= -J^{12}_{12,18},\ (C_{\mathbf{k}})_{12,19} = -J^{12}_{12,19},\ (C_{\mathbf{k}})_{12,22} = -J^{12}_{12,22},\ (C_{\mathbf{k}})_{12,23} = -J^{12}_{12,23},\\
(C_{\mathbf{k}})_{13,3} &= -J^{12}_{13,3},\ (C_{\mathbf{k}})_{13,4} = -J^{12}_{13,4},\ (C_{\mathbf{k}})_{13,7} = -J^{12}_{13,7},\\
(C_{\mathbf{k}})_{13,8} &= -J^{12}_{13,8},\ (C_{\mathbf{k}})_{13,11} = -J^{12}_{13,11},\ (C_{\mathbf{k}})_{13,12} = -J^{12}_{13,12},\\
(C_{\mathbf{k}})_{14,7} &= -J^{12}_{14,7},\ (C_{\mathbf{k}})_{14,8} = -J^{12}_{14,8},\ (C_{\mathbf{k}})_{14,11} = -J^{12}_{14,11},\ (C_{\mathbf{k}})_{14,12} = -J^{12}_{14,12},\\
(C_{\mathbf{k}})_{14,15} &= -J^{12}_{14,15},\ (C_{\mathbf{k}})_{14,16} = -J^{12}_{14,16},\ (C_{\mathbf{k}})_{14,19} = -J^{12}_{14,19},\ (C_{\mathbf{k}})_{14,20} = -J^{12}_{14,20},\\
(C_{\mathbf{k}})_{15,15} &= -J^{12}_{15,15},\ (C_{\mathbf{k}})_{15,16} = -J^{12}_{15,16},\ (C_{\mathbf{k}})_{15,19} = -J^{12}_{15,19},\\
(C_{\mathbf{k}})_{15,20} &= -J^{12}_{15,20},\ (C_{\mathbf{k}})_{15,23} = -J^{12}_{15,23},\ (C_{\mathbf{k}})_{15,24} = -J^{12}_{15,24},\\
(C_{\mathbf{k}})_{16,3} &= -J^{12}_{16,3}e^{i\mathbf{k}\cdot\delta_1},\ (C_{\mathbf{k}})_{16,4} = -J^{12}_{16,4}e^{i\mathbf{k}\cdot\delta_1},\ (C_{\mathbf{k}})_{16,7} = -J^{12}_{16,7}e^{i\mathbf{k}\cdot\delta_1},\ (C_{\mathbf{k}})_{16,8} = -J^{12}_{16,8}e^{i\mathbf{k}\cdot\delta_1},\\
(C_{\mathbf{k}})_{16,19} &= -J^{12}_{16,19},\ (C_{\mathbf{k}})_{16,20} = -J^{12}_{16,20},\ (C_{\mathbf{k}})_{16,23} = -J^{12}_{16,23},\ (C_{\mathbf{k}})_{16,24} = -J^{12}_{16,24},\\
(C_{\mathbf{k}})_{17,1} &= -J^{12}_{17,1}e^{i\mathbf{k}\cdot\delta_3},\ (C_{\mathbf{k}})_{17,3} = -J^{12}_{17,3},\ (C_{\mathbf{k}})_{17,4} = -J^{12}_{17,4},\\
(C_{\mathbf{k}})_{17,5} &= -J^{12}_{17,5}e^{i\mathbf{k}\cdot\delta_3},\ (C_{\mathbf{k}})_{17,7} = -J^{12}_{17,7},\ (C_{\mathbf{k}})_{17,8} = -J^{12}_{17,8},\\
(C_{\mathbf{k}})_{17,9} &= -J^{12}_{17,9}e^{i\mathbf{k}\cdot\delta_3},\ (C_{\mathbf{k}})_{17,11} = -J^{12}_{17,11},\ (C_{\mathbf{k}})_{17,12} = -J^{12}_{17,12},\\
(C_{\mathbf{k}})_{18,8} &= -J^{12}_{18,8},\ (C_{\mathbf{k}})_{18,9} = -J^{12}_{18,9}e^{i\mathbf{k}\cdot\delta_3},\ (C_{\mathbf{k}})_{18,11} = -J^{12}_{18,11},\ (C_{\mathbf{k}})_{18,12} = -J^{12}_{18,12},\\
(C_{\mathbf{k}})_{18,13} &= -J^{12}_{18,13}e^{i\mathbf{k}\cdot\delta_3},\ (C_{\mathbf{k}})_{18,15} = -J^{12}_{18,15},\ (C_{\mathbf{k}})_{18,16} = -J^{12}_{18,16},\ (C_{\mathbf{k}})_{18,20} = -J^{12}_{18,20},\\
(C_{\mathbf{k}})_{19,13} &= -J^{12}_{19,13}e^{i\mathbf{k}\cdot\delta_3},\ (C_{\mathbf{k}})_{19,15} = -J^{12}_{19,15},\ (C_{\mathbf{k}})_{19,16} = -J^{12}_{19,16},\\
(C_{\mathbf{k}})_{19,17} &= -J^{12}_{19,17}e^{i\mathbf{k}\cdot\delta_3},\ (C_{\mathbf{k}})_{19,19} = -J^{12}_{19,19},\ (C_{\mathbf{k}})_{19,20} = -J^{12}_{19,20},\\
(C_{\mathbf{k}})_{19,21} &= -J^{12}_{19,21}e^{i\mathbf{k}\cdot\delta_3},\ (C_{\mathbf{k}})_{19,23} = -J^{12}_{19,23},\ (C_{\mathbf{k}})_{19,24} = -J^{12}_{19,24},\\
(C_{\mathbf{k}})_{20,1} &= -J^{12}_{20,1}e^{i\mathbf{k}\cdot\delta_5}\ (C_{\mathbf{k}})_{20,3} = -J^{12}_{20,3}e^{i\mathbf{k}\cdot\delta_1},\ (C_{\mathbf{k}})_{20,4} = -J^{12}_{20,4}e^{i\mathbf{k}\cdot\delta_1},\ (C_{\mathbf{k}})_{20,8} = -J^{12}_{20,8}e^{i\mathbf{k}\cdot\delta_1},
\end{aligned}
$$

$$(C_{\mathbf{k}})_{20,20} = -J^{12}_{20,20},\ (C_{\mathbf{k}})_{20,21} = -J^{12}_{20,21}e^{i\mathbf{k}\cdot\delta_3},\ (C_{\mathbf{k}})_{20,23} = -J^{12}_{20,23},\ (C_{\mathbf{k}})_{20,24} = -J^{12}_{20,24},$$

$$(C_{\mathbf{k}})_{21,1} = -J^{12}_{21,1}e^{i\mathbf{k}\cdot\delta_3},\ (C_{\mathbf{k}})_{21,4} = -J^{12}_{21,4},\ (C_{\mathbf{k}})_{21,5} = -J^{12}_{21,5}e^{i\mathbf{k}\cdot\delta_3},$$

$$(C_{\mathbf{k}})_{21,8} = -J^{12}_{21,8},\ (C_{\mathbf{k}})_{21,9} = -J^{12}_{21,9}e^{i\mathbf{k}\cdot\delta_3},\ (C_{\mathbf{k}})_{21,12} = -J^{12}_{21,12},$$

$$(C_{\mathbf{k}})_{22,5} = -J^{12}_{22,5}e^{i\mathbf{k}\cdot\delta_3},\ (C_{\mathbf{k}})_{22,8} = -J^{12}_{22,8},\ (C_{\mathbf{k}})_{22,9} = -J^{12}_{22,9}e^{i\mathbf{k}\cdot\delta_3},\ (C_{\mathbf{k}})_{22,12} = -J^{12}_{22,12},$$

$$(C_{\mathbf{k}})_{22,13} = -J^{12}_{22,13}e^{i\mathbf{k}\cdot\delta_3},\ (C_{\mathbf{k}})_{22,16} = -J^{12}_{22,16},\ (C_{\mathbf{k}})_{22,17} = -J^{12}_{22,17}e^{i\mathbf{k}\cdot\delta_3},\ (C_{\mathbf{k}})_{22,20} = -J^{12}_{22,20},$$

$$(C_{\mathbf{k}})_{23,13} = -J^{12}_{23,13}e^{i\mathbf{k}\cdot\delta_3},\ (C_{\mathbf{k}})_{23,16} = -J^{12}_{23,16},\ (C_{\mathbf{k}})_{23,17} = -J^{12}_{23,17}e^{i\mathbf{k}\cdot\delta_3},$$

$$(C_{\mathbf{k}})_{23,20} = -J^{12}_{23,20},\ (C_{\mathbf{k}})_{23,21} = -J^{12}_{23,21}e^{i\mathbf{k}\cdot\delta_3},\ (C_{\mathbf{k}})_{23,24} = -J^{12}_{23,24},$$

$$(C_{\mathbf{k}})_{24,1} = -J^{12}_{24,1}e^{i\mathbf{k}\cdot\delta_5},\ (C_{\mathbf{k}})_{24,4} = -J^{12}_{24,4}e^{i\mathbf{k}\cdot\delta_1},\ (C_{\mathbf{k}})_{24,5} = -J^{12}_{24,5}e^{i\mathbf{k}\cdot\delta_5},\ (C_{\mathbf{k}})_{24,8} = -J^{12}_{24,8}e^{i\mathbf{k}\cdot\delta_1},$$

$$(C_{\mathbf{k}})_{24,17} = -J^{12}_{24,17}e^{i\mathbf{k}\cdot\delta_3},\ (C_{\mathbf{k}})_{24,20} = -J^{12}_{24,20},\ (C_{\mathbf{k}})_{24,21} = -J^{12}_{24,21}e^{i\mathbf{k}\cdot\delta_3},\ (C_{\mathbf{k}})_{24,24} = -J^{12}_{24,24},$$

We note that $\delta_1 = \mathbf{a},\ \delta_2 = -\mathbf{a},\ \delta_3 = \mathbf{b},\ \delta_4 = -\mathbf{b},\ \delta_5 = \mathbf{a}+\mathbf{b},\ \delta_6 = -(\mathbf{a}+\mathbf{b}),\ \delta_7 = \mathbf{a}-\mathbf{b}$ and $\delta_8 = -(\mathbf{a}-\mathbf{b})$.

$$
\begin{aligned}
(A_{\mathbf{k}})_{1,1} &= J^{1}_{1,2}+J^{1}_{1,3}+J^{1}_{1,4}+J^{1}_{1,5}+J^{1}_{1,6}+J^{1}_{1,7}+J^{1}_{1,8}+J^{1}_{1,12}+J^{1}_{1,14}\\
&- J^{12}_{1,2}-J^{12}_{1,3}-J^{12}_{1,7}-J^{12}_{1,9}-J^{12}_{1,10}+2K\\
(A_{\mathbf{k}})_{1,2} &= -J^{1}_{1,2}, (A_{\mathbf{k}})_{1,3} = -J^{1}_{1,3}e^{i\mathbf{k}\cdot\delta_2}, (A_{\mathbf{k}})_{1,4} = -J^{1}_{1,4}e^{i\mathbf{k}\cdot\delta_8}, (A_{\mathbf{k}})_{1,5} = -J^{1}_{1,5}, (A_{\mathbf{k}})_{1,6} = -J^{1}_{1,6}\\
(A_{\mathbf{k}})_{1,7} &= -J^{1}_{1,7}e^{i\mathbf{k}\cdot\delta_2}, (A_{\mathbf{k}})_{1,8} = -J^{1}_{1,8}, (A_{\mathbf{k}})_{1,12} = -J^{1}_{1,12}, (A_{\mathbf{k}})_{1,14} = -J^{1}_{1,14}e^{i\mathbf{k}\cdot\delta_2}\\
(A_{\mathbf{k}})_{2,2} &= J^{1}_{2,1}+J^{1}_{2,3}+J^{1}_{2,4}+J^{1}_{2,5}+J^{1}_{2,6}+J^{1}_{2,7}+J^{1}_{2,8}+J^{1}_{2,9}+J^{1}_{2,13}\\
&- J^{12}_{2,3}-J^{12}_{2,4}-J^{12}_{2,6}-J^{12}_{2,10}-J^{12}_{2,12}-J^{12}_{2,13}+2K\\
(A_{\mathbf{k}})_{2,3} &= -J^{1}_{2,3}, (A_{\mathbf{k}})_{2,4} = -J^{1}_{2,4}e^{i\mathbf{k}\cdot\delta_8}, (A_{\mathbf{k}})_{2,5} = -J^{1}_{2,5}e^{i\mathbf{k}\cdot\delta_3}, (A_{\mathbf{k}})_{2,6} = -J^{1}_{2,6}\\
(A_{\mathbf{k}})_{2,7} &= -J^{1}_{2,7}, (A_{\mathbf{k}})_{2,8} = -J^{1}_{2,8}, (A_{\mathbf{k}})_{2,9} = -J^{1}_{2,9}, (A_{\mathbf{k}})_{2,13} = -J^{1}_{2,13}\\
(A_{\mathbf{k}})_{3,3} &= J^{1}_{3,1}+J^{1}_{3,2}+J^{1}_{3,4}+J^{1}_{3,5}+J^{1}_{3,6}+J^{1}_{3,7}+J^{1}_{3,9}+J^{1}_{3,10}+J^{1}_{3,14}\\
&- J^{12}_{3,4}-J^{12}_{3,5}-J^{12}_{3,7}-J^{12}_{3,11}-J^{12}_{3,13}-J^{12}_{3,14}+2K\\
(A_{\mathbf{k}})_{3,4} &= -J^{1}_{3,4}e^{i\mathbf{k}\cdot\delta_3}, (A_{\mathbf{k}})_{3,5} = -J^{1}_{3,5}e^{i\mathbf{k}\cdot\delta_3}, (A_{\mathbf{k}})_{3,6} = -J^{1}_{3,6}e^{i\mathbf{k}\cdot\delta_3}, (A_{\mathbf{k}})_{3,7} = -J^{1}_{3,7}\\
(A_{\mathbf{k}})_{3,9} &= -J^{1}_{3,9}, (A_{\mathbf{k}})_{3,10} = -J^{1}_{3,10}e^{i\mathbf{k}\cdot\delta_3}, (A_{\mathbf{k}})_{3,14} = -J^{1}_{3,14}\\
(A_{\mathbf{k}})_{4,4} &= J^{1}_{4,1}+J^{1}_{4,2}+J^{1}_{4,3}+J^{1}_{4,5}+J^{1}_{4,6}+J^{1}_{4,7}+J^{1}_{4,8}+J^{1}_{4,10}+J^{1}_{4,11}-J^{12}_{4,1}-J^{12}_{4,8}-J^{12}_{4,10}-J^{12}_{4,14}+2K\\
(A_{\mathbf{k}})_{4,5} &= -J^{1}_{4,5}e^{i\mathbf{k}\cdot\delta_1}, (A_{\mathbf{k}})_{4,6} = -J^{1}_{4,6}, (A_{\mathbf{k}})_{4,7} = -J^{1}_{4,7}\\
(A_{\mathbf{k}})_{4,8} &= -J^{1}_{4,8}e^{i\mathbf{k}\cdot\delta_7}, (A_{\mathbf{k}})_{4,10} = -J^{1}_{4,10}, (A_{\mathbf{k}})_{4,11} = -J^{1}_{4,11}e^{i\mathbf{k}\cdot\delta_1}\\
(A_{\mathbf{k}})_{5,5} &= J^{1}_{5,1}+J^{1}_{5,2}+J^{1}_{5,3}+J^{1}_{5,4}+J^{1}_{5,6}+J^{1}_{5,7}+J^{1}_{5,9}+J^{1}_{5,11}+J^{1}_{5,12}\\
&- J^{12}_{5,2}-J^{12}_{5,4}-J^{12}_{5,5}-J^{12}_{5,8}-J^{12}_{5,11}+2K\\
(A_{\mathbf{k}})_{5,6} &= -J^{1}_{5,6}, (A_{\mathbf{k}})_{5,7} = -J^{1}_{5,7}e^{i\mathbf{k}\cdot\delta_2}, (A_{\mathbf{k}})_{5,9} = -J^{1}_{5,9}e^{i\mathbf{k}\cdot\delta_4}, (A_{\mathbf{k}})_{5,11} = -J^{1}_{5,11}, (A_{\mathbf{k}})_{5,12} = -J^{1}_{5,12}\\
(A_{\mathbf{k}})_{6,6} &= J^{1}_{6,1}+J^{1}_{6,2}+J^{1}_{6,3}+J^{1}_{6,4}+J^{1}_{6,5}+J^{1}_{6,7}+J^{1}_{6,10}+J^{1}_{6,12}+J^{1}_{6,13}\\
&- J^{12}_{6,3}-J^{12}_{6,5}-J^{12}_{6,6}-J^{12}_{6,12}-J^{12}_{6,14}+2K\\
(A_{\mathbf{k}})_{6,7} &= -J^{1}_{6,7}, (A_{\mathbf{k}})_{6,10} = -J^{1}_{6,10}, (A_{\mathbf{k}})_{6,12} = -J^{1}_{6,12}, (A_{\mathbf{k}})_{6,13} = -J^{1}_{6,13}\\
(A_{\mathbf{k}})_{7,7} &= J^{1}_{7,1}+J^{1}_{7,2}+J^{1}_{7,3}+J^{1}_{7,4}+J^{1}_{7,5}+J^{1}_{7,6}+J^{1}_{7,11}+J^{1}_{7,13}+J^{1}_{7,14}\\
&- J^{12}_{7,2}-J^{12}_{7,6}-J^{12}_{7,7}-J^{12}_{7,8}-J^{12}_{7,9}-J^{12}_{7,13}+2K\\
(A_{\mathbf{k}})_{7,11} &= -J^{1}_{7,11}e^{i\mathbf{k}\cdot\delta_1}, (A_{\mathbf{k}})_{7,13} = -J^{1}_{7,13}, (A_{\mathbf{k}})_{7,14} = -J^{1}_{7,14}\\
(A_{\mathbf{k}})_{8,8} &= J^{1}_{8,1}+J^{1}_{8,2}+J^{1}_{8,4}+J^{1}_{8,9}+J^{1}_{8,10}+J^{1}_{8,11}+J^{1}_{8,12}+J^{1}_{8,13}+J^{1}_{8,14}\\
&- J^{12}_{8,1}-J^{12}_{8,3}-J^{12}_{8,4}-J^{12}_{8,9}-J^{12}_{8,10}+2K\\
(A_{\mathbf{k}})_{8,9} &= -J^{1}_{8,9}, (A_{\mathbf{k}})_{8,10} = -J^{1}_{8,10}e^{i\mathbf{k}\cdot\delta_8}, (A_{\mathbf{k}})_{8,11} = -J^{1}_{8,11}e^{i\mathbf{k}\cdot\delta_3}\\
(A_{\mathbf{k}})_{8,12} &= -J^{1}_{8,12}, (A_{\mathbf{k}})_{8,13} = -J^{1}_{8,13}, (A_{\mathbf{k}})_{8,14} = -J^{1}_{8,14}e^{i\mathbf{k}\cdot\delta_2}\\
(A_{\mathbf{k}})_{9,9} &= J^{1}_{9,2}+J^{1}_{9,3}+J^{1}_{9,5}+J^{1}_{9,8}+J^{1}_{9,10}+J^{1}_{9,11}+J^{1}_{9,12}+J^{1}_{9,13}+J^{1}_{9,14}
\end{aligned}
$$

$$
\begin{aligned}
& - J^{12}_{9,4} - J^{12}_{9,5} - J^{12}_{9,10} - J^{12}_{9,11} - J^{12}_{9,13} + 2K \\
(A_{\mathbf{k}})_{9,10} &= -J^{1}_{9,10}e^{i\mathbf{k}\cdot\delta_3}, (A_{\mathbf{k}})_{9,11} = -J^{1}_{9,11}e^{i\mathbf{k}\cdot\delta_3}, (A_{\mathbf{k}})_{9,12} = -J^{1}_{9,12}e^{i\mathbf{k}\cdot\delta_3}, (A_{\mathbf{k}})_{9,13} = -J^{1}_{9,13}, (A_{\mathbf{k}})_{9,14} = -J^{1}_{9,14} \\
(A_{\mathbf{k}})_{10,10} &= J^{1}_{10,3} + J^{1}_{10,4} + J^{1}_{10,6} + J^{1}_{10,8} + J^{1}_{10,9} + J^{1}_{10,11} + J^{1}_{10,12} + J^{1}_{10,13} + J^{1}_{10,14} \\
& - J^{12}_{10,1} - J^{12}_{10,5} - J^{12}_{10,7} - J^{12}_{10,12} - J^{12}_{10,14} + 2K \\
(A_{\mathbf{k}})_{10,11} &= -J^{1}_{10,11}e^{i\mathbf{k}\cdot\delta_1}, (A_{\mathbf{k}})_{10,12} = -J^{1}_{10,12}, (A_{\mathbf{k}})_{10,13} = -J^{1}_{10,13}, (A_{\mathbf{k}})_{10,14} = -J^{1}_{10,14}e^{i\mathbf{k}\cdot\delta_4} \\
(A_{\mathbf{k}})_{11,11} &= J^{1}_{11,4} + J^{1}_{11,5} + J^{1}_{11,7} + J^{1}_{11,8} + J^{1}_{11,9} + J^{1}_{11,10} + J^{1}_{11,12} + J^{1}_{11,13} + J^{1}_{11,14} \\
& - J^{12}_{11,1} - J^{12}_{11,2} - J^{12}_{11,6} - J^{12}_{11,8} - J^{12}_{11,11} + 2K \\
(A_{\mathbf{k}})_{11,12} &= -J^{1}_{11,12}, (A_{\mathbf{k}})_{11,13} = -J^{1}_{11,13}e^{i\mathbf{k}\cdot\delta_2}, (A_{\mathbf{k}})_{11,14} = -J^{1}_{11,14}e^{i\mathbf{k}\cdot\delta_2} \\
(A_{\mathbf{k}})_{12,12} &= J^{1}_{12,1} + J^{1}_{12,5} + J^{1}_{12,6} + J^{1}_{12,8} + J^{1}_{12,9} + J^{1}_{12,10} + J^{1}_{12,11} + J^{1}_{12,13} + J^{1}_{12,14} \\
& - J^{12}_{12,2} - J^{12}_{12,3} - J^{12}_{12,5} - J^{12}_{12,9} - J^{12}_{12,11} - J^{12}_{12,12} + 2K \\
(A_{\mathbf{k}})_{12,13} &= -J^{1}_{12,13}, (A_{\mathbf{k}})_{12,14} = -J^{1}_{12,14}e^{i\mathbf{k}\cdot\delta_2} \\
(A_{\mathbf{k}})_{13,13} &= J^{1}_{13,2} + J^{1}_{13,6} + J^{1}_{13,7} + J^{1}_{13,8} + J^{1}_{13,9} + J^{1}_{13,10} + J^{1}_{13,11} + J^{1}_{13,12} + J^{1}_{13,14} \\
& - J^{12}_{13,3} - J^{12}_{13,6} - J^{12}_{13,8} - J^{12}_{13,12} - J^{12}_{13,13} + 2K \\
(A_{\mathbf{k}})_{13,14} &= -J^{1}_{13,14} \\
(A_{\mathbf{k}})_{14,14} &= J^{1}_{14,1} + J^{1}_{14,3} + J^{1}_{14,7} + J^{1}_{14,8} + J^{1}_{14,9} + J^{1}_{14,10} + J^{1}_{14,11} + J^{1}_{14,12} + J^{1}_{14,13} \\
& - J^{12}_{14,2} - J^{12}_{14,7} - J^{12}_{14,9} - J^{12}_{14,13} - J^{12}_{14,14} + 2K \\
(B_{\mathbf{k}})_{1,1} &= J^{2}_{1,2} + J^{2}_{1,3} + J^{2}_{1,4} + J^{2}_{1,5} + J^{2}_{1,6} + J^{2}_{1,7} + J^{2}_{1,8} + J^{2}_{1,10} + J^{2}_{1,14} \\
& - J^{12}_{4,1} - J^{12}_{8,1} - J^{12}_{10,1} - J^{12}_{11,1} + 2K \\
(B_{\mathbf{k}})_{1,2} &= -J^{1}_{1,2}e^{i\mathbf{k}\cdot\delta_1}, (B_{\mathbf{k}})_{1,3} = -J^{1}_{1,3}e^{i\mathbf{k}\cdot\delta_7}, (B_{\mathbf{k}})_{1,4} = -J^{1}_{1,4}e^{i\mathbf{k}\cdot\delta_7}, (B_{\mathbf{k}})_{1,5} = -J^{1}_{1,5}, (B_{\mathbf{k}})_{1,6} = -J^{1}_{1,6} \\
(B_{\mathbf{k}})_{1,7} &= -J^{1}_{1,7}e^{i\mathbf{k}\cdot\delta_4}, (B_{\mathbf{k}})_{1,8} = -J^{1}_{1,8}, (B_{\mathbf{k}})_{1,10} = -J^{1}_{1,10}e^{i\mathbf{k}\cdot\delta_7}, (B_{\mathbf{k}})_{1,14} = -J^{1}_{1,14}e^{i\mathbf{k}\cdot\delta_4} \\
(B_{\mathbf{k}})_{2,2} &= J^{2}_{2,1} + J^{2}_{2,3} + J^{2}_{2,4} + J^{2}_{2,5} + J^{2}_{2,6} + J^{2}_{2,7} + J^{2}_{2,8} + J^{2}_{2,9} + J^{2}_{2,11} \\
& - J^{12}_{1,2} - J^{12}_{5,2} - J^{12}_{7,2} - J^{12}_{11,2} - J^{12}_{12,2} - J^{12}_{14,2} + 2K \\
(B_{\mathbf{k}})_{2,3} &= -J^{1}_{2,3}, (B_{\mathbf{k}})_{2,4} = -J^{1}_{2,4}e^{i\mathbf{k}\cdot\delta_4}, (B_{\mathbf{k}})_{2,5} = -J^{1}_{2,5}, (B_{\mathbf{k}})_{2,6} = -J^{1}_{2,6}e^{i\mathbf{k}\cdot\delta_2}, \\
(B_{\mathbf{k}})_{2,7} &= -J^{1}_{2,7}e^{i\mathbf{k}\cdot\delta_2}, (B_{\mathbf{k}})_{2,8} = -J^{1}_{2,8}e^{i\mathbf{k}\cdot\delta_2}, (B_{\mathbf{k}})_{2,9} = -J^{1}_{2,9}, (B_{\mathbf{k}})_{2,11} = -J^{1}_{2,11} \\
(B_{\mathbf{k}})_{3,3} &= J^{2}_{3,1} + J^{2}_{3,2} + J^{2}_{3,4} + J^{2}_{3,5} + J^{2}_{3,6} + J^{2}_{3,7} + J^{2}_{3,9} + J^{2}_{3,10} + J^{2}_{3,12} \\
& - J^{12}_{1,3} - J^{12}_{2,3} - J^{12}_{6,3} - J^{12}_{8,3} - J^{12}_{12,3} - J^{12}_{13,3} + 2K \\
(B_{\mathbf{k}})_{3,4} &= -J^{1}_{3,4}, (B_{\mathbf{k}})_{3,5} = -J^{1}_{3,5}, (B_{\mathbf{k}})_{3,6} = -J^{1}_{3,6}, (B_{\mathbf{k}})_{3,7} = -J^{1}_{3,7}e^{i\mathbf{k}\cdot\delta_2} \\
(B_{\mathbf{k}})_{3,9} &= -J^{1}_{3,9}, (B_{\mathbf{k}})_{3,10} = -J^{1}_{3,10}, (B_{\mathbf{k}})_{3,12} = -J^{1}_{3,12} \\
(B_{\mathbf{k}})_{4,4} &= J^{2}_{4,1} + J^{2}_{4,2} + J^{2}_{4,3} + J^{2}_{4,5} + J^{2}_{4,6} + J^{2}_{4,7} + J^{2}_{4,10} + J^{2}_{4,11} + J^{2}_{4,13} \\
& - J^{12}_{2,4} - J^{12}_{3,4} - J^{12}_{5,4} - J^{12}_{8,4} - J^{12}_{9,4} + 2K
\end{aligned}
$$

$$
\begin{aligned}
(B_{\mathbf{k}})_{4,5} &= -J^1_{4,5}e^{i\mathbf{k}\cdot\delta_3}, (B_{\mathbf{k}})_{4,6} = -J^1_{4,6}, (B_{\mathbf{k}})_{4,7} = -J^1_{4,7} \\
(B_{\mathbf{k}})_{4,10} &= -J^1_{4,10}, (B_{\mathbf{k}})_{4,11} = -J^1_{4,11}e^{i\mathbf{k}\cdot\delta_3}, (B_{\mathbf{k}})_{4,13} = -J^1_{4,13} \\
(B_{\mathbf{k}})_{5,5} &= J^2_{5,1} + J^2_{5,2} + J^2_{5,3} + J^2_{5,4} + J^2_{5,6} + J^2_{5,7} + J^2_{5,11} + J^2_{5,12} + J^2_{5,14} \\
&- J^{12}_{3,5} - J^{12}_{5,5} - J^{12}_{6,5} - J^{12}_{9,5} - J^{12}_{10,5} - J^{12}_{12,5} + 2K \\
(B_{\mathbf{k}})_{5,6} &= -J^1_{5,6}, (B_{\mathbf{k}})_{5,7} = -J^1_{5,7}e^{i\mathbf{k}\cdot\delta_4}, (B_{\mathbf{k}})_{5,11} = -J^1_{5,11}, (B_{\mathbf{k}})_{5,12} = -J^1_{5,12}, (B_{\mathbf{k}})_{5,14} = -J^1_{5,14}e^{i\mathbf{k}\cdot\delta_4} \\
(B_{\mathbf{k}})_{6,6} &= J^2_{6,1} + J^2_{6,2} + J^2_{6,3} + J^2_{6,4} + J^2_{6,5} + J^2_{6,7} + J^2_{6,8} + J^2_{6,12} + J^2_{6,13} \\
&- J^{12}_{2,6} - J^{12}_{6,6} - J^{12}_{7,6} - J^{12}_{11,6} - J^{12}_{13,6} + 2K \\
(B_{\mathbf{k}})_{6,7} &= -J^1_{6,7}, (B_{\mathbf{k}})_{6,8} = -J^1_{6,8}, (B_{\mathbf{k}})_{6,12} = -J^1_{6,12}, (B_{\mathbf{k}})_{6,13} = -J^1_{6,13} \\
(B_{\mathbf{k}})_{7,7} &= J^2_{7,1} + J^2_{7,2} + J^2_{7,3} + J^2_{7,4} + J^2_{7,5} + J^2_{7,6} + J^2_{7,9} + J^2_{7,13} + J^2_{7,14} \\
&- J^{12}_{1,7} - J^{12}_{3,7} - J^{12}_{7,7} - J^{12}_{10,7} - J^{12}_{14,7} + 2K \\
(B_{\mathbf{k}})_{7,9} &= -J^1_{7,9}e^{i\mathbf{k}\cdot\delta_1}, (B_{\mathbf{k}})_{7,13} = -J^1_{7,13}, (B_{\mathbf{k}})_{7,14} = -J^1_{7,14} \\
(B_{\mathbf{k}})_{8,8} &= J^2_{8,1} + J^2_{8,2} + J^2_{8,6} + J^2_{8,9} + J^2_{8,10} + J^2_{8,11} + J^2_{8,12} + J^2_{8,13} + J^2_{8,14} \\
&- J^{12}_{4,8} - J^{12}_{5,8} - J^{12}_{7,8} - J^{12}_{11,8} - J^{12}_{13,8} + 2K \\
(B_{\mathbf{k}})_{8,9} &= -J^1_{8,9}e^{i\mathbf{k}\cdot\delta_1}, (B_{\mathbf{k}})_{8,10} = -J^1_{8,10}e^{i\mathbf{k}\cdot\delta_7}, (B_{\mathbf{k}})_{8,11} = -J^1_{8,11}e^{i\mathbf{k}\cdot\delta_1} \\
(B_{\mathbf{k}})_{8,12} &= -J^1_{8,12}, (B_{\mathbf{k}})_{8,13} = -J^1_{8,13}, (B_{\mathbf{k}})_{8,14} = -J^1_{8,14}e^{i\mathbf{k}\cdot\delta_4} \\
(B_{\mathbf{k}})_{9,9} &= J^2_{9,2} + J^2_{9,3} + J^2_{9,7} + J^2_{9,8} + J^2_{9,10} + J^2_{9,11} + J^2_{9,12} + J^2_{9,13} + J^2_{9,14} \\
&- J^{12}_{1,9} - J^{12}_{7,9} - J^{12}_{8,9} - J^{12}_{12,9} - J^{12}_{14,9} + 2K \\
(B_{\mathbf{k}})_{9,10} &= -J^1_{9,10}, (B_{\mathbf{k}})_{9,11} = -J^1_{9,11}, (B_{\mathbf{k}})_{9,12} = -J^1_{9,12}, (B_{\mathbf{k}})_{9,13} = -J^1_{9,13}e^{i\mathbf{k}\cdot\delta_2}, (B_{\mathbf{k}})_{9,14} = -J^1_{9,14}e^{i\mathbf{k}\cdot\delta_2} \\
(B_{\mathbf{k}})_{10,10} &= J^2_{10,1} + J^2_{10,3} + J^2_{10,4} + J^2_{10,8} + J^2_{10,9} + J^2_{10,11} + J^2_{10,12} + J^2_{10,13} + J^2_{10,14} \\
&- J^{12}_{1,10} - J^{12}_{2,10} - J^{12}_{4,10} - J^{12}_{8,10} - J^{12}_{9,10} + 2K \\
(B_{\mathbf{k}})_{10,11} &= -J^1_{10,11}e^{i\mathbf{k}\cdot\delta_3}, (B_{\mathbf{k}})_{10,12} = -J^1_{10,12}, (B_{\mathbf{k}})_{10,13} = -J^1_{10,13}, (B_{\mathbf{k}})_{10,14} = -J^1_{10,14}e^{i\mathbf{k}\cdot\delta_2} \\
(B_{\mathbf{k}})_{11,11} &= J^2_{11,2} + J^2_{11,4} + J^2_{11,5} + J^2_{11,8} + J^2_{11,9} + J^2_{11,10} + J^2_{11,12} + J^2_{11,13} + J^2_{11,14} \\
&- J^{12}_{3,11} - J^{12}_{5,11} - J^{12}_{9,11} - J^{12}_{11,11} - J^{12}_{12,11} + 2K \\
(B_{\mathbf{k}})_{11,12} &= -J^1_{11,12}, (B_{\mathbf{k}})_{11,13} = -J^1_{11,13}e^{i\mathbf{k}\cdot\delta_4}, (B_{\mathbf{k}})_{11,14} = -J^1_{11,14}e^{i\mathbf{k}\cdot\delta_4} \\
(B_{\mathbf{k}})_{12,12} &= J^2_{12,3} + J^2_{12,5} + J^2_{12,6} + J^2_{12,8} + J^2_{12,9} + J^2_{12,10} + J^2_{12,11} + J^2_{12,13} + J^2_{12,14} \\
&- J^{12}_{2,12} - J^{12}_{6,12} - J^{12}_{10,12} - J^{12}_{12,12} - J^{12}_{13,12} + 2K \\
(B_{\mathbf{k}})_{12,13} &= -J^1_{12,13}, (B_{\mathbf{k}})_{12,14} = -J^1_{12,14}e^{i\mathbf{k}\cdot\delta_4} \\
(B_{\mathbf{k}})_{13,13} &= J^2_{13,4} + J^2_{13,6} + J^2_{13,7} + J^2_{13,8} + J^2_{13,9} + J^2_{13,10} + J^2_{13,11} + J^2_{13,12} + J^2_{13,14} \\
&- J^{12}_{2,13} - J^{12}_{3,13} - J^{12}_{7,13} - J^{12}_{9,13} - J^{12}_{13,13} - J^{12}_{14,13} + 2K \\
(B_{\mathbf{k}})_{13,14} &= -J^1_{13,14}
\end{aligned}
$$

$$
\begin{aligned}
(B_{\mathbf{k}})_{14,14} &= J^2_{14,1} + J^2_{14,5} + J^2_{14,7} + J^2_{14,8} + J^2_{14,9} + J^2_{14,10} + J^2_{14,11} + J^2_{14,12} + J^2_{14,13} \\
&- J^{12}_{3,14} - J^{12}_{4,14} - J^{12}_{6,14} - J^{12}_{10,14} - J^{12}_{14,14} + 2K \\
(C_{\mathbf{k}})_{1,2} &= -J^{12}_{1,2}, (C_{\mathbf{k}})_{1,3} = -J^{12}_{1,3}, (C_{\mathbf{k}})_{1,7} = -J^{12}_{1,7}e^{i\mathbf{k}\cdot\delta_1}, (C_{\mathbf{k}})_{1,9} = -J^{12}_{1,9}, (C_{\mathbf{k}})_{1,11} = -J^{12}_{1,10}, \\
(C_{\mathbf{k}})_{2,3} &= -J^{12}_{2,3}, (C_{\mathbf{k}})_{2,4} = -J^{12}_{2,4}, (C_{\mathbf{k}})_{2,6} = -J^{12}_{2,6}, (C_{\mathbf{k}})_{2,10} = -J^{12}_{2,10}, (C_{\mathbf{k}})_{2,12} = -J^{12}_{2,12}, (C_{\mathbf{k}})_{2,13} = -J^{12}_{2,13}, \\
(C_{\mathbf{k}})_{3,4} &= -J^{12}_{3,4}, (C_{\mathbf{k}})_{3,5} = -J^{12}_{3,5}e^{i\mathbf{k}\cdot\delta_3}, (C_{\mathbf{k}})_{3,7} = -J^{12}_{3,7}, (C_{\mathbf{k}})_{3,11} = -J^{12}_{3,11}e^{i\mathbf{k}\cdot\delta_3}, (C_{\mathbf{k}})_{3,13} = -J^{12}_{3,13}, (C_{\mathbf{k}})_{3,14} = -J^{12}_{3,14}, \\
(C_{\mathbf{k}})_{4,1} &= -J^{12}_{4,1}, (C_{\mathbf{k}})_{4,8} = -J^{12}_{4,8}, (C_{\mathbf{k}})_{4,10} = -J^{12}_{4,10}e^{i\mathbf{k}\cdot\delta_7}, (C_{\mathbf{k}})_{4,14} = -J^{12}_{4,14}e^{i\mathbf{k}\cdot\delta_4}, \\
(C_{\mathbf{k}})_{5,2} &= -J^{12}_{5,2}, (C_{\mathbf{k}})_{5,4} = -J^{12}_{5,4}e^{i\mathbf{k}\cdot\delta_4}, (C_{\mathbf{k}})_{5,5} = -J^{12}_{5,5}, (C_{\mathbf{k}})_{5,8} = -J^{12}_{5,8}e^{i\mathbf{k}\cdot\delta_2}, (C_{\mathbf{k}})_{5,11} = -J^{12}_{5,11}, \\
(C_{\mathbf{k}})_{6,3} &= -J^{12}_{6,3}, (C_{\mathbf{k}})_{6,5} = -J^{12}_{6,5}, (C_{\mathbf{k}})_{6,6} = -J^{12}_{6,6}, (C_{\mathbf{k}})_{6,12} = -J^{12}_{6,12}, (C_{\mathbf{k}})_{6,14} = -J^{12}_{6,14}e^{i\mathbf{k}\cdot\delta_4}, \\
(C_{\mathbf{k}})_{7,2} &= -J^{12}_{7,2}e^{i\mathbf{k}\cdot\delta_1}, (C_{\mathbf{k}})_{7,6} = -J^{12}_{7,6}, (C_{\mathbf{k}})_{7,7} = -J^{12}_{7,7}, (C_{\mathbf{k}})_{7,8} = -J^{12}_{7,8}, (C_{\mathbf{k}})_{7,9} = -J^{12}_{7,9}e^{i\mathbf{k}\cdot\delta_1}, (C_{\mathbf{k}})_{7,13} = -J^{12}_{7,13}, \\
(C_{\mathbf{k}})_{8,1} &= -J^{12}_{8,1}e^{i\mathbf{k}\cdot\delta_8}, (C_{\mathbf{k}})_{8,3} = -J^{12}_{8,3}, (C_{\mathbf{k}})_{8,4} = -J^{12}_{8,4}, (C_{\mathbf{k}})_{8,9} = -J^{12}_{8,9}, (C_{\mathbf{k}})_{8,10} = -J^{12}_{8,10}, \\
(C_{\mathbf{k}})_{9,4} &= -J^{12}_{9,4}, (C_{\mathbf{k}})_{9,5} = -J^{12}_{9,5}e^{i\mathbf{k}\cdot\delta_3}, (C_{\mathbf{k}})_{9,10} = -J^{12}_{9,10}, (C_{\mathbf{k}})_{9,11} = -J^{12}_{9,11}e^{i\mathbf{k}\cdot\delta_3}, (C_{\mathbf{k}})_{9,13} = -J^{12}_{9,13}, \\
(C_{\mathbf{k}})_{10,1} &= -J^{12}_{10,1}, (C_{\mathbf{k}})_{10,5} = -J^{12}_{10,5}, (C_{\mathbf{k}})_{10,7} = -J^{12}_{10,7}e^{i\mathbf{k}\cdot\delta_4}, (C_{\mathbf{k}})_{10,12} = -J^{12}_{10,12}, (C_{\mathbf{k}})_{10,14} = -J^{12}_{10,14}e^{i\mathbf{k}\cdot\delta_4}, \\
(C_{\mathbf{k}})_{11,1} &= -J^{12}_{11,1}e^{i\mathbf{k}\cdot\delta_2}, (C_{\mathbf{k}})_{11,2} = -J^{12}_{11,2}, (C_{\mathbf{k}})_{11,6} = -J^{12}_{11,6}e^{i\mathbf{k}\cdot\delta_2}, (C_{\mathbf{k}})_{11,8} = -J^{12}_{11,8}e^{i\mathbf{k}\cdot\delta_2}, (C_{\mathbf{k}})_{11,11} = -J^{12}_{11,11}, \\
(C_{\mathbf{k}})_{12,2} &= -J^{12}_{12,2}, (C_{\mathbf{k}})_{12,3} = -J^{12}_{12,3}, (C_{\mathbf{k}})_{12,5} = -J^{12}_{12,5}, (C_{\mathbf{k}})_{12,9} = -J^{12}_{12,9}, (C_{\mathbf{k}})_{12,11} = -J^{12}_{12,11}, (C_{\mathbf{k}})_{12,12} = -J^{12}_{12,12}, \\
(C_{\mathbf{k}})_{13,3} &= -J^{12}_{13,3}, (C_{\mathbf{k}})_{13,6} = -J^{12}_{13,6}, (C_{\mathbf{k}})_{13,8} = -J^{12}_{13,8}, (C_{\mathbf{k}})_{13,12} = -J^{12}_{13,12}, (C_{\mathbf{k}})_{13,13} = -J^{12}_{13,13}, \\
(C_{\mathbf{k}})_{14,2} &= -J^{12}_{14,2}e^{i\mathbf{k}\cdot\delta_1}, (C_{\mathbf{k}})_{14,7} = -J^{12}_{14,7}, (C_{\mathbf{k}})_{14,9} = -J^{12}_{14,9}e^{i\mathbf{k}\cdot\delta_1}, (C_{\mathbf{k}})_{14,13} = -J^{12}_{14,13}, (C_{\mathbf{k}})_{14,14} = -J^{12}_{14,14}
\end{aligned}
$$

The relationship $(A_{\mathbf{k}})_{m,n} = \left[(A_{\mathbf{k}})_{n,m}\right]^*$ and $(B_{\mathbf{k}})_{m,n} = \left[(B_{\mathbf{k}})_{n,m}\right]^*$ is always hold for the Hermitian matrix, which is shown in *Appendix A*. Therefore, for the simplicity, only half of the non-diagonal elements of $A_{\mathbf{k}}$ and $B_{\mathbf{k}}$ elements are given in *Appendix B*. We note that $\delta_1 = \mathbf{a}$, $\delta_2 = -\mathbf{a}$, $\delta_3 = \mathbf{b}$, $\delta_4 = -\mathbf{b}$, $\delta_5 = \mathbf{a}+\mathbf{b}$, $\delta_6 = -(\mathbf{a}+\mathbf{b})$, $\delta_7 = \mathbf{a}-\mathbf{b}$ and $\delta_8 = -(\mathbf{a}-\mathbf{b})$.

References

[1] Liechtenstein, A. I.; Katsnelson, M. I.; Gubanov, V. A. Exchange interactions and spin-wave stiffness in ferromagnetic metals. *J. Phys. F* **1984**, *14*, L125.

[2] Szilva, A.; Kvashnin, Y.; Stepanov, E. A.; Nordström, L.; Eriksson, O.; Lichtenstein, A. I.; Katsnelson, M. I. Quantitative theory of magnetic interactions in solids. *Rev. Mod. Phys.* **2023**, *95*, 035004.

[3] Smidstrup, S.; Markussen, T.; Vancraeyveld, P.; Wellendorff, J.; Schneider, J.; Gunst, T.; Verstichel, B.; Stradi, D.; Khomyakov, P. A.; Vej-Hansen, U. G.; Gronbjerg, U. QuantumATK: an integrated platform of electronic and atomic-scale modelling tools. *J. Phys.: Condens. Matter* **2020**, *32*, 015901.

[4] Melander, M.; Jónsson, E.; Mortensen, J.; Vegge, T.; Lastra, J. Implementation of Constrained DFT for Computing Charge Transfer Rates within the Projector Augmented Wave Method. *J. Chem. Theory. Comput.* **2016**, *12*, 5367–5378.

[5] Seifert, G.; Porezag, D.; Frauenheim, T. Calculations of molecules, clusters, and solids with a simplified LCAO-DFT-LDA scheme. *Int. J. Quantum Chem.* **1996**, *58*, 185–192.

[6] Kresse, G.; Hafner, J. Ab initio molecular-dynamics simulation of the liquid-metal–amorphous-semiconductor transition in germanium. *Phys. Rev. B* **1994**, *49*, 14251.

[7] Kresse, G.; Furthmuller, J. Efficient iterative schemes for ab initio total-energy calculations using a plane-wave basis set. *Phys. Rev. B* **1996**, *54*, 11169.

[8] Kresse, G.; Joubert, D. From ultrasoft pseudopotentials to the projector augmented-wave method. *Phys. Rev. B* **1999**, *59*, 1758.

[9] Grimme, S.; Antony, J.; Ehrlich, S.; Krieg, H. A consistent and accurate ab initio parametrization of density functional dispersion correction (DFT-D) for the 94 elements H–Pu. *J. Chem. Phys.* **2010**, *132*, 154104.

[10] Cui, Q.; Bai, X.; Delin, A. Anisotropic magnon transport in van der Waals ferromagnetic insulators. *Adv. Funct. Mater.* **2025**, *35*, 2407469.

[11] Bo, X.; Li, F.; Yin, X.; Chen, Y.; Wan, X.; Pu, Y. Magnetic structure and exchange interactions of the van der Waals $CrPS_4$ monolayer under strain: a first-principles study. *Phys. Rev. B* **2023**, *108*, 024405.

[12] Sadhukhan, B.; Bergman, A.; Kvashnin, Y. O.; Hellsvik, J.; Delin, A. Spin-lattice couplings in two-dimensional CrI_3 from first-principles computations. *Phys. Rev. B* **2022**, *105*, 104418.

[13] Yang, B.; Li, Y.; Xiang, H.; Lin, H.; Huang, B. Moiré magnetic exchange interactions in twisted magnets. *Nat. Comput. Sci.* **2023**, *3*, 314–320.

[14] Colpa, J. H. P. Diagonalization of the quadratic boson Hamiltonian. *Physica A* **1978**, *93*, 327–353.

[15] Kubo, R. Statistical-mechanical theory of irreversible processes. I. General theory and simple applications to magnetic and conduction problems. *J. Phys. Soc. Jpn.* **1957**, *12*, 570–586.

[16] Bartram, F. M.; Li, M.; Liu, L.; Xu, Z.; Wang, Y.; Che, M.; Li, H.; Wu, Y.; Xu, Y.; Zhang, J.; Yang, S.; Yang, L. Real-time observation of magnetization and magnon dynamics in a two-dimensional antiferromagnet $MnBi_2Te_4$. *Sci. Bull.* **2023**, *68*, 2734–2742.

[17] Liao, M.; Wei, Z.; Du, L.; Wang, Q.; Tang, J.; Yu, H.; Wu, F.; Zhao, J.; Xu, X.; Han, B.; et al. Precise control of the interlayer twist angle in large scale MoS_2 homostructures. *Nat. Commun.* **2020**, *11*, 2153.

[18] Constant, C. B.; Jenkins, S.; Eiroa, R. R.; Santos, E. J. G.; Valero, S. M.; Coronado, E. Multistep magnetization switching in orthogonally twisted ferromagnetic monolayers. *Nat. Mater.* **2024**, *23*, 212–218.

[19] Wu, Y.; Deng, L.; Yin, X.; Tong, J.; Tian, F.; Zhang, X. Valley-related multipiezo effect and noncollinear spin current in an altermagnet Fe_2Se_2O monolayer. *Nano Lett.* **2024**, *24*, 10534–10539.

[20] Liu, C.; Li, X.; Li, X.; Yang, J. Realizing abundant two-dimensional altermagnets with anisotropic spin current via spatial inversion symmetry breaking. *Nano Lett.* **2025**, *25*, 9197–9203.

[21] Rezende, S. M.; Azevedo, A.; Rodríguez-Suárez, R. L. Introduction to antiferromagnetic magnons. *J. Appl. Phys.* **2019**, *126*, 151101.

[22] Šmejkal, L.; Marmodoro, A.; Ahn, K.-H.; G.-Hernández, R.; Turek, I.; Mankovsky, S.; Ebert, H.; D'Souza, S. W.; Šipr, O.; Sinova, J.; Jungwirth, T. Chiral magnons in altermagnetic RuO_2. *Phys. Rev. Lett.* **2023**, *131*, 256703.
